# Supplementary material for: Life histories predict genetic diversity and population structure within three species of octopus targeted by small-scale fisheries in Northwest Mexico
Source: PeerJ. 2018 Feb 15;6:e4295. doi: 10.7717/peerj.4295 (PMC5816968; doi:10.7717/peerj.4295)
Supplement: Table S1 — Months in which the samples from 20 localities were collected in northwest Mexico 2008-2013. [file peerj-06-4295-s001.docx]

|  | **Month** | | | | | | | | | | | |
| --- | --- | --- | --- | --- | --- | --- | --- | --- | --- | --- | --- | --- |
| **Location** | Jan | Feb | Mar | Apr | May | Jun | Jul | Aug | Sep | Oct | Nov | Dec |
| Ejido Erendira |  |  |  |  |  |  |  |  |  |  |  |  |
| San Quintin |  |  |  |  |  |  |  |  |  |  |  |  |
| Bahía Magdalena |  |  |  |  |  |  |  |  |  |  |  |  |
| Puerto Libertad |  |  |  |  |  |  |  |  |  |  |  |  |
| Isla San Lorenzo |  |  |  |  |  |  |  |  |  |  |  |  |
| Isla Tiburon |  |  |  |  |  |  |  |  |  |  |  |  |
| Bahia Kino |  |  |  |  |  |  |  |  |  |  |  |  |
| Santa Rosalia |  |  |  |  |  |  |  |  |  |  |  |  |
| El conejo |  |  |  |  |  |  |  |  |  |  |  |  |
| La Bocana |  |  |  |  |  |  |  |  |  |  |  |  |
| Las Barrancas |  |  |  |  |  |  |  |  |  |  |  |  |
| Malarrino |  |  |  |  |  |  |  |  |  |  |  |  |
| Puerto Peñasco |  |  |  |  |  |  |  |  |  |  |  |  |
| San Luis Gonzaga |  |  |  |  |  |  |  |  |  |  |  |  |
| Puerto Refugio |  |  |  |  |  |  |  |  |  |  |  |  |
| Isla Smith |  |  |  |  |  |  |  |  |  |  |  |  |
| Bahia de los Angeles |  |  |  |  |  |  |  |  |  |  |  |  |
| Puerto Lobos |  |  |  |  |  |  |  |  |  |  |  |  |
| Isla San Pedro Martir |  |  |  |  |  |  |  |  |  |  |  |  |
| Isla Datil |  |  |  |  |  |  |  |  |  |  |  |  |
