# Supplementary material for: Life histories predict genetic diversity and population structure within three species of octopus targeted by small-scale fisheries in Northwest Mexico
Source: PeerJ. 2018 Feb 15;6:e4295. doi: 10.7717/peerj.4295 (PMC5816968; doi:10.7717/peerj.4295)
Supplement: Table S2 — Tests of Hardy-Weinberg equilibrium in 20 populations of octopus genotyped at 7 loci. Estimation of exact P-values by the Markov chain method. Parameters: 10,000 dememorization, 1000 batches and 10,000 iterations per batch. [file peerj-06-4295-s002.docx]

|  |  |  |  |  |  |  |  |  |  | **by Population** | |
| --- | --- | --- | --- | --- | --- | --- | --- | --- | --- | --- | --- |
| **Species** | **Code** | **Location** | **Ocbi-25** | **Ocbi-35** | **Ocbi-39** | **Ocbi-41** | **Ocbi-47** | **Ocbi-48** | **Ocbi-50** | **Probability** | **Bonferroni** |
| *O. bimaculoides* |  | Ejido Erendira | 0.2439 | 0.6646 | 0.0127 | **0.0005** | 0.6564 | 1.0000 | 0.5384 | 0.0085 | 0.000357 |
|  |  | San Quintin | 1.0000 | 0.8532 | 0.0015 | 0.3832 | **0.0008** | 1.0000 | 1.0000 | 0.0090 |  |
|  |  | Bahía Magdalena | 0.0303 | 0.5407 | 0.0502 | 0.0105 | 0.4821 | 0.7591 | 0.4814 | 0.0205 |  |
| *O. bimaculatus* |  | La Bocana | 1.0000 | 1.0000 | 0.0474 | 1.0000 | 1.0000 | 0.4892 | 0.1747 | 0.6847 |  |
|  |  | Las Barrancas | 0.3644 | 1.0000 | 0.6173 | 1.0000 | 0.3110 | 0.3133 | 0.1114 | 0.6039 |  |
|  |  | El conejo | 0.4468 | 0.5114 | 0.4905 | 0.4872 | 0.0278 | 0.5107 | Monomorphic | 0.2804 |  |
|  |  | Malarrino | 0.2256 | 0.5619 | 0.0030 | 0.7531 | 0.3956 | 0.4446 | 0.0080 | 0.0091 |  |
|  |  | Pto. Peñasco | 0.4931 | 0.6963 | 0.4988 | 0.4480 | 0.9176 | 0.0417 | 0.8159 | 0.6009 |  |
|  |  | San Luis Gonzaga | 1.0000 | 0.9208 | 0.1569 | 0.3496 | 1.0000 | 0.6823 | Monomorphic | 0.8745 |  |
|  |  | Pto. Refugio | 0.2719 | 0.8499 | 0.1407 | 0.4298 | 0.7607 | **0.0001** | 0.1327 | 0.0033 |  |
|  |  | Isla Smith | 0.8337 | 0.9586 | 0.0240 | 0.9466 | 0.6572 | 0.2789 | 0.7712 | 0.6116 |  |
|  |  | Bahia de los Angeles | 0.4403 | 0.1968 | 0.0004 | 0.7654 | 0.9487 | 0.0045 | 0.0646 | 0.0007 |  |
|  |  | Puerto Lobos | 0.7367 | 0.0124 | 0.0973 | 0.3077 | 0.0057 | 0.4022 | **0.0001** | 0.0000 |  |
| *O. hubbsorum* |  | Puerto Libertad | 0.2450 | 0.1922 | 0.0180 | **0.0001** | 0.0024 | 0.0588 | 1.0000 | 0.0000 |  |
|  |  | Isla San Lorenzo | 0.8748 | 0.9997 | 0.3380 | 0.6941 | 0.0037 | 0.0117 | Monomorphic | 0.0256 |  |
|  |  | Isla Tiburon | 0.0534 | 0.8785 | 0.0090 | 0.0020 | 0.0323 | 0.0173 | **0.0001** | 0.0000 |  |
|  |  | Bahia Kino | 0.3703 | 0.8942 | 0.0011 | **0.0000** | 0.1612 | 0.4048 | Monomorphic | 0.0000 |  |
|  |  | Sta. Rosalia | 0.4369 | 1.0000 | 0.0769 | 0.1386 | 1.0000 | 0.8207 | Monomorphic | 0.5175 |  |
|  |  | ISPM | 0.2000 | 1.0000 | 1.0000 | Monomorphic | 0.3324 | 0.2002 | Monomorphic | 0.5667 |  |
|  |  | El datil | 0.5999 | 1.0000 | 1.0000 | 1.0000 | 0.5990 | 1.0000 | 0.4666 | 0.9975 |  |
|  |  |  |  |  |  |  |  |  |  |  |  |
|  | **By locus** | **Probability** | 0.4756 | 0.9909 | 0.0000 | 0.0000 | 0.0004 | 0.0004 | 0.0000 |  |  |
|  |  | **Bonferroni** | 0.0025 | | | | | | |  |  |
