# Supplementary material for: Life histories predict genetic diversity and population structure within three species of octopus targeted by small-scale fisheries in Northwest Mexico
Source: PeerJ. 2018 Feb 15;6:e4295. doi: 10.7717/peerj.4295 (PMC5816968; doi:10.7717/peerj.4295)
Supplement: Table S3 — Average null allele frequency obtained with FREENA using the Expectation Maximization algorithm of Dempster 1977 in 20 populations of octopus genotyped at 7 loci. [file peerj-06-4295-s003.docx]

|  |  |  | **Estimate of null allele frequency** | | | | | | | **Mean** | **Mean** |
| --- | --- | --- | --- | --- | --- | --- | --- | --- | --- | --- | --- |
| **Species** | **Code** | **Location** | **Ocbi- 25** | **Ocbi- 35** | **Ocbi- 39** | **Ocbi- 41** | **Ocbi- 47** | **Ocbi- 48** | **Ocbi- 50** | **population** | **species** |
| *O. bimaculoides* |  | Ejido Erendira | 0.000 | 0.000 | 0.000 | 0.000 | 0.000 | 0.000 | 0.000 | 0.000 |  |
|  |  | San Quintin | 0.000 | 0.000 | 0.172 | 0.070 | 0.265 | 0.000 | 0.000 | 0.072 |  |
|  |  | Bahía Magdalena | 0.038 | 0.000 | 0.000 | 0.000 | 0.000 | 0.000 | 0.000 | 0.005 | **0.026** |
| *O. bimaculatus* |  | La Bocana | 0.000 | 0.000 | 0.001 | 0.000 | 0.000 | 0.000 | 0.000 | 0.000 |  |
|  |  | Las Barrancas | 0.000 | 0.000 | 0.000 | 0.000 | 0.001 | 0.083 | 0.001 | 0.012 |  |
|  |  | El conejo | 0.054 | 0.000 | 0.072 | 0.000 | 0.000 | 0.000 | 0.001 | 0.018 |  |
|  |  | Malarrino | 0.033 | 0.000 | 0.102 | 0.000 | 0.000 | 0.032 | 0.045 | 0.030 |  |
|  |  | Pto. Peñasco | 0.000 | 0.000 | 0.000 | 0.000 | 0.000 | 0.000 | 0.000 | 0.000 |  |
|  |  | San Luis Gonzaga | 0.000 | 0.000 | 0.000 | 0.000 | 0.000 | 0.010 | 0.001 | 0.002 |  |
|  |  | Pto. Refugio | 0.005 | 0.000 | 0.127 | 0.000 | 0.000 | 0.220 | 0.103 | 0.065 |  |
|  |  | Isla Smith | 0.000 | 0.000 | 0.027 | 0.000 | 0.000 | 0.000 | 0.000 | 0.004 |  |
|  |  | Bahia de los Angeles | 0.000 | 0.000 | 0.255 | 0.000 | 0.000 | 0.158 | 0.000 | 0.059 |  |
|  |  | Puerto Lobos | 0.000 | 0.089 | 0.073 | 0.015 | 0.000 | 0.000 | 0.165 | 0.049 | **0.025** |
| *O. hubbsorum* |  | Puerto Libertad | 0.006 | 0.000 | 0.157 | 0.000 | 0.083 | 0.064 | 0.000 | 0.044 |  |
|  |  | Isla San Lorenzo | 0.000 | 0.000 | 0.000 | 0.000 | 0.165 | 0.167 | 0.001 | 0.048 |  |
|  |  | Isla Tiburon | 0.078 | 0.000 | 0.095 | 0.062 | 0.111 | 0.148 | 0.263 | 0.108 |  |
|  |  | Bahia Kino | 0.000 | 0.000 | 0.000 | 0.000 | 0.000 | 0.000 | 0.001 | 0.000 |  |
|  |  | Sta. Rosalia | 0.000 | 0.000 | 0.136 | 0.000 | 0.000 | 0.007 | 0.000 | 0.020 |  |
|  |  | ISPM | 0.315 | 0.000 | 0.000 | 0.001 | 0.001 | 0.176 | 0.001 | 0.071 |  |
|  |  | El datil | 0.000 | 0.000 | 0.111 | 0.000 | 0.000 | 0.000 | 0.000 | 0.016 | **0.041** |
|  |  |  |  |  |  |  |  |  |  |  |  |
|  |  | **Mean locus** | **0.026** | **0.004** | **0.066** | **0.007** | **0.031** | **0.053** | **0.029** |  |  |
