# Supplementary material for: Life histories predict genetic diversity and population structure within three species of octopus targeted by small-scale fisheries in Northwest Mexico
Source: PeerJ. 2018 Feb 15;6:e4295. doi: 10.7717/peerj.4295 (PMC5816968; doi:10.7717/peerj.4295)
Supplement: Table S4 — Individuals that could not be assigned under the criterion of at least 2/3 of assignment probability to a single species, and that show show assignment probabilities shared between two species. (*) Indicates the samples that were assigned with mtDNA (16s rDNA and COI) and a higher probability of assignment by microsatellites. [file peerj-06-4295-s004.docx]

| **Locality** | ***O. bimaculoides*** | ***O. hubbsorum*** | ***O. bimaculatus*** | **(%) sum** |
| --- | --- | --- | --- | --- |
| Malarrimo | 0.28 | 0.16 | 0.56 | 0.84 |
| Puerto Peñasco | 0.05 | 0.36 | 0.59* | 0.95 |
| Puerto Peñasco | 0.02 | 0.43 | 0.55 | 0.98 |
| Puerto Peñasco | 0.03 | 0.44 | 0.53 | 0.97 |
| Puerto Peñasco | 0.02 | 0.52 | 0.46 | 0.98 |
| Puerto Peñasco | 0.05 | 0.35 | 0.60 | 0.95 |
| Puerto Peñasco | 0.38 | 0.24 | 0.38 | 0.76 |
| Puerto Peñasco | 0.01 | 0.56 | 0.43 | 0.99 |
| Puerto Peñasco | 0.05 | 0.38 | 0.57 | 0.95 |
| Puerto Refugio | 0.15 | 0.29 | 0.56 | 0.85 |
| Puerto Refugio | 0.12 | 0.60 | 0.28 | 0.88 |
| Puerto Refugio | 0.33 | 0.25 | 0.42 | 0.75 |
| Puerto Refugio | 0.42 | 0.06 | 0.52 | 0.94 |
| Puerto Libertad | 0.38 | 0.08 | 0.54* | 0.92 |
| Puerto Libertad | 0.61 | 0.22 | 0.17 | 0.83 |
| Puerto Libertad | 0.30 | 0.62* | 0.08 | 0.92 |
| Puerto Libertad | 0.64 | 0.06 | 0.30 | 0.94 |
| Puerto Libertad | 0.11 | 0.28 | 0.61 | 0.89 |
| Isla Dátil | 0.56 | 0.24 | 0.20 | 0.80 |
