# Supplementary material for: Life histories predict genetic diversity and population structure within three species of octopus targeted by small-scale fisheries in Northwest Mexico
Source: PeerJ. 2018 Feb 15;6:e4295. doi: 10.7717/peerj.4295 (PMC5816968; doi:10.7717/peerj.4295)
Supplement: Table S5 — Probability of assignment for individuals identified in the limit of the geographic distribution of the species to which they belong. The values in bold indicate the highest observed assignment probability to a single species (microsatellites). (*) Possible hybrids identified by a mismatch between mtDNA (16s rDNA and COI) and microsatellite assignment. [file peerj-06-4295-s005.docx]

|  |  | **% ancestry observed** | | |
| --- | --- | --- | --- | --- |
| **Locality** | **species expected** | ***O. bimaculoides*** | ***O. hubbsorum*** | ***O. bimaculatus*** |
| La Bocana | *O. bimaculatus* | **0.87** | 0.01 | 0.12 |
| Las Barrancas | *O. bimaculatus* | 0.01 | **0.84** | 0.15 |
| Puerto Peñasco* | *O. bimaculatus* | 0.04 | **0.73*** | 0.23 |
| Puerto Peñasco* | *O. bimaculatus* | 0.03 | **0.92*** | 0.05 |
| Puerto Peñasco | *O. bimaculatus* | 0.16 | **0.73** | 0.10 |
| Puerto Refugio | *O. bimaculatus* | **0.74** | 0.18 | 0.08 |
| Puerto Libertad | *O. hubbsorum* | **0.68** | 0.19 | 0.13 |
| Isla Tiburón | *O. hubbsorum* | 0.02 | 0.17 | **0.80** |
| Isla Dátil | *O. hubbsorum* | **0.93** | 0.04 | 0.03 |
| Isla Dátil | *O. hubbsorum* | 0.07 | 0.07 | **0.86** |
| Isla S. Pedro Martir | *O. hubbsorum* | **0.97** | 0.02 | 0.01 |
