# Supplementary material for: Life histories predict genetic diversity and population structure within three species of octopus targeted by small-scale fisheries in Northwest Mexico
Source: PeerJ. 2018 Feb 15;6:e4295. doi: 10.7717/peerj.4295 (PMC5816968; doi:10.7717/peerj.4295)
Supplement: Table S6 — Estimates of FST values with and without considering null alleles according to the software FREENA. Confidence intervals (CI) calculated using 10,000 bootstraps. [file peerj-06-4295-s006.docx]

| **Species** | **Correction F_ST_** | **All Loci** | **10^3^ Bootstraps CI (95%)** | |
| --- | --- | --- | --- | --- |
|  |  |  | **Lower** | **Upper** |
|  |  |  |  |  |
| ***O. bimaculoides*** | **Not using ENA** | 0.214246 | 0.105172 | 0.344751 |
|  | **Using ENA** | 0.221643 | 0.114926 | 0.351331 |
|  |  |  |  |  |
| ***O. bimaculatus*** | **Not using ENA** | 0.092940 | 0.013590 | 0.232639 |
|  | **Using ENA** | 0.088880 | 0.014279 | 0.221188 |
|  |  |  |  |  |
| ***O. hubbsorum*** | **Not using ENA** | 0.102621 | 0.019955 | 0.252214 |
|  | **Using ENA** | 0.110793 | 0.022214 | 0.271400 |
