# Supplementary material for: Life histories predict genetic diversity and population structure within three species of octopus targeted by small-scale fisheries in Northwest Mexico
Source: PeerJ. 2018 Feb 15;6:e4295. doi: 10.7717/peerj.4295 (PMC5816968; doi:10.7717/peerj.4295)
Supplement: Figure S1 — (A) Delta K is the mean of the absolute values, (B) mean and standard deviation of Ln probability of data for No. of genetic cluster (K) = 1 to 20 and (C) bar plot showing the mean individual assignment probabilities among 10 independent replicates of both K = 2 and K = 3. [file peerj-06-4295-s007.pdf]

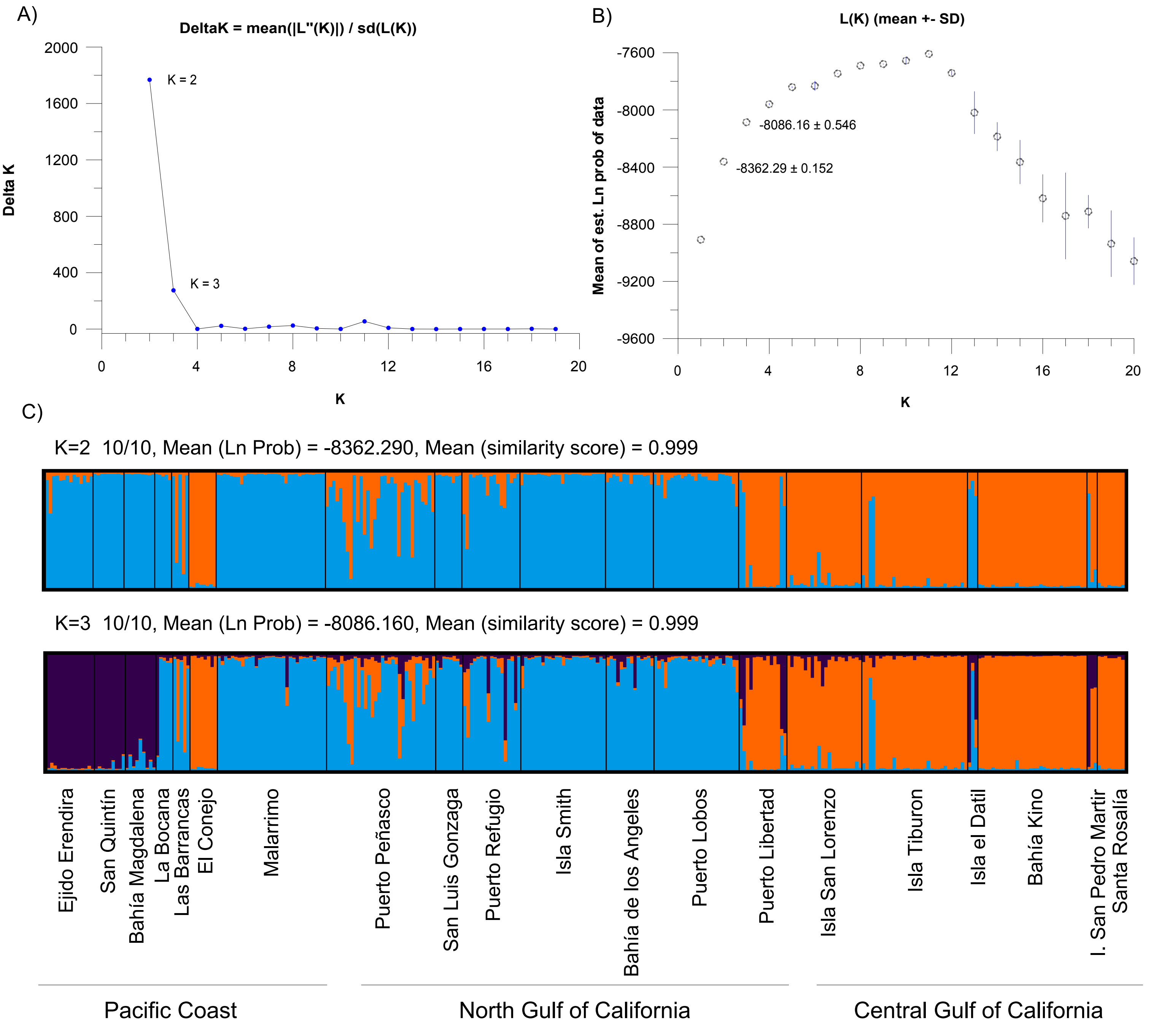

A)
DeltaK = mean(|L''(K)|) / sd(L(K))
Delta K
K
K = 2
K = 3
B)
L(K) (mean +- SD)
Mean of est. Ln prob of data
K
-8086.16 ± 0.546
-8362.29 ± 0.152
C)
K=2 10/10, Mean (Ln Prob) = -8362.290, Mean (similarity score) = 0.999
K=3 10/10, Mean (Ln Prob) = -8086.160, Mean (similarity score) = 0.999
Ejido Erendira
San Quintín
Bahía Magdalena
La Bocana
Las Barrancas
El Conejo
Malarrimo
Puerto Peñasco
San Luis Gonzaga
Puerto Refugio
Isla Smith
Bahía de los Angeles
Puerto Lobos
Puerto Libertad
Isla San Lorenzo
Isla Tiburon
Isla el Datil
Bahía Kino
I. San Pedro Martir
Santa Rosalía
Pacific Coast
North Gulf of California
Central Gulf of California
