# Supplementary material for: Life histories predict genetic diversity and population structure within three species of octopus targeted by small-scale fisheries in Northwest Mexico
Source: PeerJ. 2018 Feb 15;6:e4295. doi: 10.7717/peerj.4295 (PMC5816968; doi:10.7717/peerj.4295)
Supplement: Supplemental Information 1 [file peerj-06-4295-s008.pdf]

>STR184 COI
AGGTACCTCTCTAAGTTTAATAATTCGAACAGAATTAGGTCAACCAGGATCTCTCCTAAATGATGATCAACTATATAACGTAATTGTTACAGCACATGCATTT
GTAATAATTTTTTTTCTTGTTATACCTGTTATAATTGGAGGATTTGGTAATTGATTAGTTCCCTTAATACTAGGAGCTCCAGACATAGCATTCCCACGAATAA
ACAATATAAGTTTTTGACTTTTACCACCTTCTCTTACCCTATTATTATCTTCAGCTGCAGTAGAAAGAGGTGCAGGTACTGGATGGACTGTATATCCCCCTCT
TTCAAGAAATTTAGCTCATATAGGCCCTTCCGTTGATCTAGCTATTTTTTCTCTTCATTTAGCTGGTATTTCATCTATTCTTGGAGCCATTAACTTCATTACA
ACTATTATCAATATACGATGAGAAGGAATATTAATAGAACGACTTCCATTATTTGTATGATCAGTATTTATTACCGCAATTTTATTACTTCTTTCCTTACCAG
TCCTTGCTGGAGCAATTACAATACTCTTAACAGATCGAAATTTTAATACCACTTTTTTTGACCCAA
>STR183 COI
AGGTACCTCTCTAAGTTTAATAATTCGAACAGAATTAGGTCAACCAGGATCTCTCCTAAATGATGATCAACTATATAACGTAATTGTTACAGCACATGCATTT
GTAATAATTTTTTTTCTTGTTATACCTGTTATAATTGGAGGATTTGGTAATTGATTAGTTCCCTTAATACTAGGAGCTCCAGACATAGCATTCCCACGAATAA
ACAATATAAGTTTTTGACTTTTACCACCTTCTCTTACCCTATTATTATCTTCAGCTGCAGTAGAAAGAGGTGCAGGTACTGGATGGACTGTATATCCCCCTCT
TTCAAGAAATTTAGCTCATATAGGCCCTTCCGTTGATCTAGCTATTTTTTCTCTTCATTTAGCTGGTATTTCATCTATTCTTGGAGCCATTAACTTCATTACA
ACTATTATCAATATACGATGAGAAGGAATATTAATAGAACGACTTCCATTATTTGTATGATCAGTATTTATTACCGCAATTTTATTACTTCTTTCCTTACCAG
TCCTTGCTGGAGCAATTACAATACTCTTAACAGATCGAAATTTTAATACCACTTTTTTTGACCCAA
>STR182 COI
AGGTACCTCTCTAAGTTTAATAATTCGAACAGAATTAGGTCAACCAGGATCTCTCCTAAATGATGATCAACTATATAACGTAATTGTTACAGCACATGCATTT
GTAATAATTTTTTTTCTTGTTATACCTGTTATAATTGGAGGATTTGGTAATTGATTAGTTCCCTTAATACTAGGAGCTCCAGACATAGCATTCCCACGAATAA
ACAATATAAGTTTTTGACTTTTACCACCTTCTCTTACCCTATTATTATCTTCAGCTGCAGTAGAAAGAGGTGCAGGTACTGGATGGACTGTATATCCCCCTCT
TTCAAGAAATTTAGCTCATATAGGCCCTTCCGTTGATCTAGCTATTTTTTCTCTTCATTTAGCTGGTATTTCATCTATTCTTGGAGCCATTAACTTCATTACA
ACTATTATCAATATACGATGAGAAGGAATATTAATAGAACGACTTCCATTATTTGTATGATCAGTATTTATTACCGCAATTTTATTACTTCTTTCCTTACCAG
TCCTTGCTGGAGCAATTACAATACTCTTAACAGATCGAAATTTTAATACCACTTTTTTTGACCCAA
>STR181 COI
AGGTACCTCTCTAAGTTTAATAATTCGAACAGAATTAGGTCAACCAGGATCTCTCCTAAATGATGATCAACTATATAACGTAATTGTTACAGCACATGCATTT
GTAATAATTTTTTTTCTTGTTATACCTGTTATAATTGGAGGATTTGGTAATTGATTAGTTCCCTTAATACTAGGAGCTCCAGACATAGCATTCCCACGAATAA
ACAATATAAGTTTTTGACTTTTACCACCTTCTCTTACCCTATTATTATCTTCAGCTGCAGTAGAAAGAGGTGCAGGTACTGGATGGACTGTATATCCCCCTCT
TTCAAGAAATTTAGCTCATATAGGCCCTTCCGTTGATCTAGCTATTTTTTCTCTTCATTTAGCTGGTATTTCATCTATTCTTGGAGCCATTAACTTCATTACA
ACTATTATCAATATACGATGAGAAGGAATATTAATAGAACGACTTCCATTATTTGTATGATCAGTATTTATTACCGCAATTTTATTACTTCTTTCCTTACCAG
TCCTTGCTGGAGCAATTACAATACTCTTAACAGATCGAAATTTTAATACCACTTTTTTTGACCCAA
>STR180 COI
AGGTACCTCTCTAAGTTTAATAATTCGAACAGAATTAGGTCAACCAGGATCTCTCCTAAATGATGATCAACTATATAACGTAATTGTTACAGCACATGCATTT
GTAATAATTTTTTTTCTTGTTATACCTGTTATAATTGGAGGATTTGGTAATTGATTAGTTCCCTTAATACTAGGAGCTCCAGACATAGCATTCCCACGAATAA
ACAATATAAGTTTTTGACTTTTACCACCTTCTCTTACCCTATTATTATCTTCAGCTGCAGTAGAAAGAGGTGCAGGTACTGGATGGACTGTATATCCCCCTCT
TTCAAGAAATTTAGCTCATATAGGCCCTTCCGTTGATCTAGCTATTTTTTCTCTTCATTTAGCTGGTATTTCATCTATTCTTGGAGCCATTAACTTCATTACA
ACTATTATCAATATACGATGAGAAGGAATATTAATAGAACGACTTCCATTATTTGTATGATCAGTATTTATTACCGCAATTTTATTACTTCTTTCCTTACCAG
TCCTTGCTGGAGCAATTACAATACTCTTAACAGATCGAAATTTTAATACCACTTTTTTTGACCCAA
>STR179 COI
AGGTACCTCTCTAAGTTTAATAATTCGAACAGAATTAGGTCAACCAGGATCTCTCCTAAATGATGATCAACTATATAACGTAATTGTTACAGCACATGCATTT
GTAATAATTTTTTTTCTTGTTATACCTGTTATAATTGGAGGATTTGGTAATTGATTAGTTCCCTTAATACTAGGAGCTCCAGACATAGCATTCCCACGAATAA
ACAATATAAGTTTTTGACTTTTACCACCTTCTCTTACCCTATTATTATCTTCAGCTGCAGTAGAAAGAGGTGCAGGTACTGGATGGACTGTATATCCCCCTCT
TTCAAGAAATTTAGCTCATATAGGCCCTTCCGTTGACCTAGCTATTTTTTCTCTTCATTTAGCTGGTATTTCATCTATTCTTGGAGCCATTAACTTCATTACA
ACTATTATCAATATACGATGAGAAGGAATATTAATAGAACGACTTCCATTATTTGTATGATCAGTATTTATTACCGCAATTTTATTACTTCTTTCCTTACCAG
TCCTTGCTGGAGCAATTACAATACTCTTAACAGATCGAAATTTTAATACCACTTTTTTTGACCCAA
>STR178 COI
AGGTACCTCTCTAAGTTTAATAATTCGAACAGAATTAGGTCAACCAGGATCTCTCCTAAATGATGATCAACTATATAACGTAATTGTTACAGCACATGCATTT
GTAATAATTTTTTTTCTTGTTATACCTGTTATAATTGGAGGATTTGGTAATTGATTAGTTCCCTTAATACTAGGAGCTCCAGACATAGCATTCCCACGAATAA
ACAATATAAGTTTTTGACTTTTACCACCTTCTCTTACCCTATTATTATCTTCAGCTGCAGTAGAAAGAGGTGCAGGTACTGGATGGACTGTATATCCCCCTCT
TTCAAGAAATTTAGCTCATATAGGCCCTTCCGTTGATCTAGCTATTTTTTCTCTTCATTTAGCTGGTATTTCATCTATTCTTGGAGCCATTAACTTCATTACA
ACTATTATCAATATACGATGAGAAGGAATATTAATAGAACGACTTCCATTATTTGTATGATCAGTATTTATTACCGCAATTTTATTACTTCTTTCCTTACCAG
TCCTTGCTGGAGCAATTACAATACTCTTAACAGATCGAAATTTTAATACCACTTTTTTTGACCCAG
>STR177 COI
AGGTACCTCTCTAAGTTTAATAATTCGAACAGAATTAGGTCAACCAGGATCTCTCCTAAATGATGATCAACTATATAACGTAATTGTTACAGCACATGCATTT
GTAATAATTTTTTTTCTTGTTATACCTGTTATAATTGGAGGATTTGGTAATTGATTAGTTCCCTTAATACTAGGAGCTCCAGACATAGCATTCCCACGAATAA
ACAATATAAGTTTTTGACTTTTACCACCTTCTCTTACCCTATTATTATCTTCAGCTGCAGTAGAAAGAGGTGCAGGTACTGGATGGACTGTATATCCCCCTCT
TTCAAGAAATTTAGCTCATATAGGCCCTTCCGTTGACCTAGCTATTTTTTCTCTTCATTTAGCTGGTATTTCATCTATTCTTGGAGCCATTAACTTCATTACA
ACTATTATCAATATACGATGAGAAGGAATATTAATAGAACGACTTCCATTATTTGTATGATCAGTATTTATTACCGCAATTTTATTACTTCTTTCCTTACCAG
TCCTTGCTGGAGCAATTACAATACTCTTAACAGATCGAAATTTTAATACCACTTTTTTTGACCCAA
>SQ96 COI
GGGTACCTCTTTAAGTTTAATAATTCGAACAGAATTAGGTCAACCAGGATCTCTTCTCAATGATGATCAATTATATAATGTTATTGTCACAGCCCATGCATTT
GTAATAATCTTTTTCCTTGTTATGCCCGTTATAATTGGAGGATTCGGCAACTGATTAGTTCCCTTAATATTAGGAGCCCCAGACATAGCATTTCCACGAATAA
ATAATATAAGTTTTTGACTTCTACCACCCTCTCTTACTTTATTACTATCCTCAGCTGCAGTAGAAAGAGGTGTAGGAACCGGATGAACCGTATACCCTCCTCT
TTCAAGAAATTTAGCTCATATAGGTCCATCCGTTGATTTAGCTATTTTTTCACTACATCTAGCTGGTATCTCATCTATTCTTGGAGCTATTAATTTTATTACA
ACTATCATTAATATACGATGAGAAGGAATACTAATAGAACGACTCCCATTATTTGTATGATCAGTATTTATTACTGCAATTTTATTATTACTTTCCTTACCAG
TCCTTGCCGGAGCAATTACAATACTTTTAACTGATCGAAATTTTAATACCACTTTCTTTGACCCAA
>SQ95 COI
GGGTACCTCTTTAAGTTTAATAATTCGAACAGAATTAGGTCAACCAGGATCTCTTCTCAATGATGATCAATTATATAATGTTATTGTCACAGCCCATGCATTT
GTAATAATCTTTTTCCTTGTTATGCCCGTTATAATTGGAGGATTCGGCAACTGATTAGTTCCCTTAATATTAGGAGCCCCAGACATAGCATTTCCACGAATAA
ATAATATAAGTTTTTGACTTCTACCACCCTCTCTTACTTTATTACTATCCTCAGCTGCAGTAGAAAGAGGTGTAGGAACCGGATGAACCGTATACCCTCCTCT
TTCAAGAAATTTAGCTCATATAGGTCCATCCGTTGATTTAGCTATTTTTTCACTACATCTAGCTGGTATCTCATCTATTCTTGGAGCTATTAATTTTATTACA
ACTATCATTAATATACGATGAGAAGGAATACTAATAGAACGACTCCCATTATTTGTATGATCAGTATTTATTACTGCAATTTTATTATTACTTTCCTTACCAG
TCCTTGCCGGAGCAATTACAATACTTTTAACTGATCGAAATTTTAATACCACTTTCTTTGACCCAA
>SQ94 COI
GGGTACCTCTTTAAGTTTAATAATTCGAACAGAATTAGGTCAACCAGGATCTCTTCTCAATGATGATCAATTATATAATGTTATTGTCACAGCCCATGCATTT
GTAATAATCTTTTTCCTTGTTATGCCCGTTATAATTGGAGGATTCGGCAACTGATTAGTTCCCTTAATATTAGGAGCCCCAGACATAGCATTTCCACGAATAA
ATAATATAAGTTTTTGACTTCTACCACCCTCTCTTACTTTATTACTATCCTCAGCTGCAGTAGAAAGAGGTGTAGGAACCGGATGAACCGTATACCCTCCTCT
TTCAAGAAATTTAGCTCATATAGGTCCATCCGTTGATTTAGCTATTTTTTCACTACATCTAGCTGGTATCTCATCTATTCTTGGAGCTATTAATTTTATTACA
ACTATCATTAATATACGATGAGAAGGAATACTAATAGAACGACTCCCATTATTTGTATGATCAGTATTTATTACTGCAATTTTATTATTACTTTCCTTACCAG
TCCTTGCCGGAGCAATTACAATACTTTTAACTGATCGAAATTTTAATACCACTTTCTTTGACCCAA
>SQ93 COI
GGGTACCTCTTTAAGTTTAATAATTCGAACAGAATTAGGTCAACCAGGATCTCTTCTCAATGATGATCAATTATATAATGTTATTGTCACAGCCCATGCATTT

```
GTAATAATCTTTTTCCTTGTTATGCCCGTTATAATTGGAGGATTCGGCAACTGATTAGTTCCCTTAATATTAGGAGCCCCAGACATAGCATTTCCACGAATAA
ATAATATAAGTTTTTGACTTCTACCACCCTCTCTTACTTTATTACTATCCTCAGCTGCAGTAGAAAGAGGTGTAGGAACCGGATGAACCGTATACCCTCCTCT
TTCAAGAAATTTAGCTCATATAGGTCCATCCGTTGATTTAGCTATTTTTTCACTACATCTAGCTGGTATCTCATCTATTCTTGGAGCTATTAATTTTATTACA
ACTATCATTAATATACGATGAGAAGGAATACTAATAGAACGACTCCCATTATTTGTATGATCAGTATTTATTACTGCAATTTTATTATTACTTTCCTTACCAG
TCCTTGCCGGAGCAATTACAATACTTTTAACTGATCGAAATTTTAATACCACTTTCTTTGACCCAA
>SQ92 COI
GGGTACCTCTTTAAGTTTAATAATTCGAACAGAATTAGGTCAACCAGGATCTCTTCTCAATGATGATCAATTATATAATGTTATTGTCACAGCCCATGCATTT
GTAATAATCTTTTTCCTTGTTATGCCCGTTATAATTGGAGGATTCGGCAACTGATTAGTTCCCTTAATATTAGGAGCCCCAGACATAGCATTTCCACGAATAA
ATAATATAAGTTTTTGACTTCTACCACCCTCTCTTACTTTATTACTATCCTCAGCTGCAGTAGAAAGAGGTGTAGGAACCGGATGAACCGTATACCCTCCTCT
TTCAAGAAATTTAGCTCATATAGGTCCATCCGTTGATTTAGCTATTTTTTCACTACATCTAGCTGGTATCTCATCTATTCTTGGAGCTATTAATTTTATTACA
ACTATCATTAATATACGATGAGAAGGAATACTAATAGAACGACTCCCATTATTTGTATGATCAGTATTTATTACTGCAATTTTATTATTACTTTCCTTACCAG
TCCTTGCCGGAGCAATTACAATACTTTTAACTGATCGAAATTTTAATACCACTTTCTTTGACCCAA
>SQ91 COI
GGGTACCTCTTTAAGTTTAATAATTCGAACAGAATTAGGTCAACCAGGATCTCTTCTCAATGATGATCAATTATATAATGTTATTGTCACAGCCCATGCATTT
GTAATAATCTTTTTCCTTGTTATGCCCGTTATAATTGGAGGATTCGGCAACTGATTAGTTCCCTTAATATTAGGAGCCCCAGACATAGCATTTCCACGAATAA
ATAATATAAGTTTTTGACTTCTACCACCCTCTCTTACTTTATTACTATCCTCAGCTGCAGTAGAAAGAGGTGTAGGAACCGGATGAACCGTATACCCTCCTCT
TTCAAGAAATTTAGCTCATATAGGTCCATCCGTTGATTTAGCTATTTTTTCACTACATCTAGCTGGTATCTCATCTATTCTTGGAGCTATTAATTTTATTACA
ACTATCATTAATATACGATGAGAAGGAATACTAATAGAACGACTCCCATTATTTGTATGATCAGTATTTATTACTGCAATTTTATTATTACTTTCCTTACCAG
TCCTTGCCGGAGCAATTACAATACTTTTAACTGATCGAAATTTTAATACCACTTTCTTTGACCCAA
>SQ90 COI
GGGTACCTCTTTAAGTTTAATAATTCGAACAGAATTAGGTCAACCAGGATCTCTTCTCAATGATGATCAATTATATAATGTTATTGTCACAGCCCATGCATTT
GTAATAATCTTTTTCCTTGTTATGCCCGTTATAATTGGAGGATTCGGCAACTGATTAGTTCCCTTAATATTAGGAGCCCCAGACATAGCATTTCCACGAATAA
ATAATATAAGTTTTTGACTTCTACCACCCTCTCTTACTTTATTACTATCCTCAGCTGCAGTAGAAAGAGGTGTAGGAACCGGATGAACCGTATACCCTCCTCT
TTCAAGAAATTTAGCTCATATAGGTCCATCCGTTGATTTAGCTATTTTTTCACTACATCTAGCTGGTATCTCATCTATTCTTGGAGCTATTAATTTTATTACA
ACTATCATTAATATACGATGAGAAGGAATACTAATAGAACGACTCCCATTATTTGTATGATCAGTATTTATTACTGCAATTTTATTATTACTTTCCTTACCAG
TCCTTGCCGGAGCAATTACAATACTTTTAACTGATCGAAATTTTAATACCACTTTCTTTGACCCAA
>SQ89 COI
GGGTACCTCTTTAAGTTTAATAATTCGAACAGAATTAGGTCAACCAGGATCTCTTCTCAATGATGATCAATTATATAATGTTATTGTCACAGCCCATGCATTT
GTAATAATCTTTTTCCTTGTTATGCCCGTTATAATTGGAGGATTCGGCAACTGATTAGTTCCCTTAATATTAGGAGCCCCAGACATAGCATTTCCACGAATAA
ATAATATAAGTTTTTGACTTCTACCACCCTCTCTTACTTTATTACTATCCTCAGCTGCAGTAGAAAGAGGTGTAGGAACCGGATGAACCGTATACCCTCCTCT
TTCAAGAAATTTAGCTCATATAGGTCCATCCGTTGATTTAGCTATTTTTTCACTACATCTAGCTGGTATCTCATCTATTCTTGGAGCTATTAATTTTATTACA
ACTATCATTAATATACGATGAGAAGGAATACTAATAGAACGACTCCCATTATTTGTATGATCAGTATTTATTACTGCAATTTTATTATTACTTTCCTTACCAG
TCCTTGCCGGAGCAATTACAATACTTTTAACTGATCGAAATTTTAATACCACTTTCTTTGACCCAA
>PRE268 COI
AGGTACTTCTTTAAGTTTAATAATTCGAACAGAATTAGGTCAACCAGGATCTCTTCTCAATGATGATCAATTATATAATGTTATTGTTACAGCCCATGCATTT
GTAATAATCTTTTTTCTTGTTATACCTGTCATAATTGGAGGATTTGGCAACTGATTAGTTCCCTTAATATTGGGAGCCCCAGATATAGCATTCCCACGAATAA
ATAACATAAGTTTTTGACTTTTACCACCCTCTCTCACTTTATTATTATCCTCAGCCGCAGTAGAAAGAGGTGTAGGAACTGGATGAACCGTATATCCCCCTCT
CTCAAGAAACTTAGCTCATATAGGTCCATCCGTTGATTTAGCTATTTTTTCACTCCACTTAGCCGGTATCTCATCTATCCTTGGAGCTATTAATTTTATTACA
ACTATTATTAATATACGATGAGAAGGAATACTAATAGAACGACTCCCATTATTTGTATGATCAGTATTTATTACTGCAATTTTATTATTACTTTCTCTACCAG
TTCTTGCTGGAGCAATTACAATACTTTTAACTGATCGAAATTTTAATACCACTTTTTTTGATCCGA
>PPE304 COI
AGGTACTTCTTTAAGTTTAATAATTCGAACAGAATTAGGTCAACCAGGATCTCTTCTCAATGATGATCAATTATATAATGTTATTGTTACAGCCCATGCATTT
GTAATAATCTTTTTTCTTGTTATACCTGTCATAATTGGAGGATTTGGCAACTGATTAGTTCCCTTAATATTGGGAGCCCCAGATATAGCATTCCCACGAATAA
ATAACATAAGTTTTTGACTTTTACCACCCTCTCTCACTTTATTATTATCCTCAGCCGCAGTAGAAAGAGGTGTAGGAACTGGATGAACCGTATATCCCCCTCT
CTCAAGAAACTTAGCTCATATAGGTCCATCCGTTGATTTAGCTATTTTTTCACTCCACTTAGCCGGTATCTCATCTATCCTTGGAGCTATTAATTTTATTACA
ACTATTATTAATATACGATGAGAAGGAATACTAATAGAACGACTCCCATTATTTGTATGATCAGTATTTATTACTGCAATTTTATTATTACTTTCTCTACCAG
TTCTTGCTGGAGCAATTACAATACTTTTAACTGATCGAAATTTTAATACCACTTTTTTTGATCCGA
>PPE303 COI
AGGTACTTCTTTAAGTTTAATAATTCGAACAGAATTAGGTCAACCAGGATCTCTTCTCAATGATGATCAATTATATAATGTTATTGTTACAGCCCATGCATTT
GTAATAATCTTTTTTCTTGTTATACCTGTCATAATTGGAGGATTTGGCAACTGATTAGTTCCCTTAATATTGGGAGCCCCAGATATAGCATTCCCACGAATAA
ATAACATAAGTTTTTGACTTTTACCACCCTCTCTCACTTTATTATTATCCTCAGCCGCAGTAGAAAGAGGTGTAGGAACTGGATGAACCGTATATCCCCCTCT
CTCAAGAAACTTAGCTCATATAGGTCCATCCGTTGATTTAGCTATTTTTTCACTCCACTTAGCCGGTATCTCATCTATCCTTGGAGCTATTAATTTTATTACA
ACTATTATTAATATACGATGAGAAGGAATACTAATAGAACGACTCCCATTATTTGTATGATCAGTATTTATTACTGCAATTTTATTATTACTTTCTCTACCAG
TTCTTGCTGGAGCAATTACAATACTTTTAACTGATCGAAATTTTAATACCACTTTTTTTGATCCGA
>PPE302 COI
AGGTACTTCTTTAAGTTTAATAATTCGAACAGAATTAGGTCAACCAGGATCTCTTCTCAATGATGATCAATTATATAATGTTATTGTTACAGCCCATGCATTT
GTAATAATCTTTTTTCTTGTTATACCTGTCATAATTGGAGGATTTGGCAACTGATTAGTTCCCTTAATATTGGGAGCCCCAGATATAGCATTCCCACGAATAA
ATAACATAAGTTTTTGACTTTTACCACCCTCTCTCACTTTATTATTATCCTCAGCCGCAGTAGAAAGAGGTGTAGGAACTGGATGAACCGTATATCCCCCTCT
CTCAAGAAACTTAGCTCATATAGGTCCATCCGTTGATTTAGCTATTTTTTCACTCCACTTAGCCGGTATCTCATCTATCCTTGGAGCTATTAATTTTATTACA
ACTATTATTAATATACGATGAGAAGGAATACTAATAGAACGACTCCCATTATTTGTATGATCAGTATTTATTACTGCAATTTTATTATTACTTTCTCTACCAG
TTCTTGCTGGAGCAATTACAATACTTTTAACTGATCGAAATTTTAATACCACTTTTTTTGATCCGA
>PPE301 COI
AGGTACTTCTTTAAGTTTAATAATTCGAACAGAATTAGGTCAACCAGGATCTCTTCTCAATGATGATCAATTATATAATGTTATTGTTACAGCCCATGCATTT
GTAATAATCTTTTTTCTTGTTATACCTGTCATAATTGGAGGATTTGGCAACTGATTAGTTCCCTTAATATTGGGAGCCCCAGATATAGCATTCCCACGAATAA
ATAACATAAGTTTTTGACTTTTACCCCCCTCTCTCACTTTATTATTATCCTCAGCCGCAGTAGAAAGAGGTGTAGGAACTGGATGAACCGTATATCCCCCTCT
CTCAAGAAACTTAGCTCATATAGGTCCATCCGTTGATTTAGCTATTTTTTCACTCCACTTAGCCGGTATCTCATCTATCCTTGGAGCTATTAATTTTATTACA
ACTATTATTAATATACGATGAGAAGGAATACTAATAGAACGACTCCCATTATTTGTATGATCAGTATTTATTACTGCAATTTTATTATTACTTTCTCTACCAG
TTCTTGCTGGAGCAATTACAATACTTTTAACTGATCGAAATTTTAATACCACTTTTTTTGATCCGA
>PPE300 COI
AGGTACTTCTTTAAGTTTAATAATTCGAACAGAATTAGGTCAACCAGGATCTCTTCTCAATGATGATCAATTATATAATGTTATTGTTACAGCCCATGCATTT
GTAATAATCTTTTTTCTTGTTATACCTGTCATAATTGGAGGATTTGGCAACTGATTAGTTCCCTTAATATTGGGAGCCCCAGATATAGCATTCCCACGAATAA
ATAACATAAGTTTTTGACTTTTACCACCCTCTCTCACTTTATTATTATCCTCAGCCGCAGTAGAAAGAGGTGTAGGAACTGGATGAACCGTATATCCCCCTCT
CTCAAGAAACTTAGCTCATATAGGTCCATCCGTTGATTTAGCTATTTTTTCACTCCACTTAGCCGGTATCTCATCTATCCTTGGAGCTATTAATTTTATTACA
ACTATTATTAATATACGATGAGAAGGAATACTAATAGAACGACTCCCATTATTTGTATGATCAGTATTTATTACTGCAATTTTATTATTACTTTCTCTACCAG
TTCTTGCTGGAGCAATTACAATACTTTTAACTGATCGAAATTTTAATACCACTTTTTTTGATCCGA
>PPE299 COI
AGGTACTTCTTTAAGTTTAATAATTCGAACAGAATTAGGTCAACCAGGATCTCTTCTCAATGATGATCAATTATATAATGTTATTGTTACAGCCCATGCATTT
GTAATAATCTTTTTTCTTGTTATACCTGTCATAATTGGAGGATTTGGCAACTGATTAGTTCCCTTAATATTGGGAGCCCCAGATATAGCATTCCCACGAATAA
ATAACATAAGTTTTTGACTTTTACCACCCTCTCTCACTTTATTATTATCCTCAGCCGCAGTAGAAAGAGGTGTAGGAACTGGATGAACCGTATATCCCCCTCT
```

CTCAAGAAACTTAGCTCATATAGGTCCATCCGTTGATTTAGCTATTTTTTCACTCCACTTAGCCGGTATCTCATCTATCCTTGGAGCTATTAATTTTATTACA
ACTATTATTAATATACGATGAGAAGGAATACTAATAGAACGACTCCCATTATTTGTATGATCAGTATTTATTACTGCAATTTTATTATTACTTTCTCTACCAG
TTCTTGCTGGAGCAATTACAATACTTTTAACTGATCGAAATTTTAATACCACTTTTTTTGATCCGA
>PPE298 COI
AGGTACTTCTTTAAGTTTAATAATTCGAACAGAATTAGGTCAACCAGGATCTCTTCTCAATGATGATCAATTATATAATGTTATTGTTACAGCCCATGCATTT
GTAATAATCTTTTTTCTTGTTATACCTGTCATAATTGGAGGATTTGGCAACTGATTAGTTCCCTTAATATTGGGAGCCCCAGATATAGCATTCCCACGAATAA
ATAACATAAGTTTTTGACTTTTACCACCCTCTCTCACTTTATTATTATCCTCAGCCGCAGTAGAAAGAGGTGTAGGAACTGGATGAACCGTATATCCCCCTCT
CTCAAGAAACTTAGCTCATATAGGTCCATCCGTTGATTTAGCTATTTTTTCACTCCACTTAGCCGGTATCTCATCTATCCTTGGAGCTATTAATTTTATTACA
ACTATTATTAATATACGATGAGAAGGAATACTAATAGAACGACTCCCATTATTTGTATGATCAGTATTTATTACTGCAATTTTATTATTACTTTCTCTACCAG
TTCTTGCTGGAGCAATTACAATACTTTTAACTGATCGAAATTTTAATACCACTTTTTTTGATCCGA
>PPE297 COI
AGGTACTTCTTTAAGTTTAATAATTCGAACAGAATTAGGTCAACCAGGATCTCTTCTCAATGATGATCAATTATATAATGTTATTGTTACAGCCCATGCATTT
GTAATAATCTTTTTTCTTGTTATACCTGTCATAATTGGAGGATTTGGCAACTGATTAGTTCCCTTAATATTGGGAGCCCCAGATATAGCATTCCCACGAATAA
ATAACATAAGTTTTTGACTTTTACCACCCTCTCTCACTTTATTATTATCCTCAGCCGCAGTAGAAAGAGGTGTAGGAACTGGATGAACCGTATATCCCCCTCT
CTCAAGAAACTTAGCTCATATAGGTCCATCCGTTGATTTAGCTATTTTTTCACTCCACTTAGCCGGTATCTCATCTATCCTTGGAGCTATTAATTTTATTACA
ACTATTATTAATATACGATGAGAAGGAATACTAATAGAACGACTCCCATTATTTGTATGATCAGTATTTATTACTGCAATTTTATTATTACTTTCTCTACCAG
TTCTTGCTGGAGCAATTACAATACTTTTAACTGATCGAAATTTTAATACCACTTTTTTTGATCCGA
>PLO363 COI
AGGTACTTCTTTAAGTTTAATAATTCGAACAGAATTAGGTCAACCAGGATCTCTTCTCAATGATGATCAATTATATAATGTTATTGTTACAGCCCATGCATTT
GTAATAATCTTTTTTCTTGTTATACCTGTCATAATTGGAGGATTTGGCAACTGATTAGTTCCCTTAATATTGGGAGCCCCAGATATAGCATTCCCACGAATAA
ATAACATAAGTTTTTGACTTTTACCACCCTCTCTCACTTTATTATTATCCTCAGCCGCAGTAGAAAGAGGTGTAGGAACTGGATGAACCGTATATCCCCCTCT
CTCAAGAAACTTAGCTCATATAGGTCCATCCGTTGATTTAGCTATTTTTTCACTCCACTTAGCCGGTATCTCATCTATCCTTGGAGCTATTAATTTTATTACA
ACTATTATTAATATACGATGAGAAGGAATACTAATAGAACGACTCCCATTATTTGTATGATCAGTATTTATTACTGCAATTTTATTATTACTTTCTCTACCAG
TTCTTGCTGGAGCAATTACAATACTTTTAACTGATCGAAATTTTAATACCACTTTTTTTGATCCGA
>PLO362 COI
AGGTACTTCTTTAAGTTTAATAATTCGAACAGAATTAGGTCAACCAGGATCTCTTCTCAATGATGATCAATTATATAATGTTATTGTTACAGCCCATGCATTT
GTAATAATCTTTTTTCTTGTTATACCTGTCATAATTGGAGGATTTGGCAACTGATTAGTTCCCTTAATATTGGGAGCCCCAGATATAGCATTCCCACGAATAA
ATAACATAAGTTTTTGACTTTTACCACCCTCTCTCACTTTATTATTATCCTCAGCCGCAGTAGAAAGAGGTGTAGGAACTGGATGAACCGTATATCCCCCTCT
CTCAAGAAACTTAGCTCATATAGGTCCATCCGTTGATTTAGCTATTTTTTCACTCCACTTAGCCGGTATCTCATCTATCCTTGGAGCTATTAATTTTATTACA
ACTATTATTAATATACGATGAGAAGGAATACTAATAGAACGACTCCCATTATTTGTATGATCAGTATTTATTACTGCAATTTTATTATTACTTTCTCTACCAG
TTCTTGCTGGAGCAATTACAATACTTTTAACTGATCGAAATTTTAATACCACTTTTTTTGATCCGA
>PLO361 COI
AGGTACTTCTTTAAGTTTAATAATTCGAACAGAATTAGGTCAACCAGGATCTCTTCTCAATGATGATCAATTATATAATGTTATTGTTACAGCCCATGCATTT
GTAATAATCTTTTTTCTTGTTATACCTGTCATAATTGGAGGATTTGGCAACTGATTAGTTCCCTTAATATTGGGAGCCCCAGATATAGCATTCCCACGAATAA
ATAACATAAGTTTTTGACTTTTACCACCCTCTCTCACTTTATTATTATCCTCAGCCGCAGTAGAAAGAGGTGTAGGAACTGGATGAACCGTATATCCCCCTCT
CTCAAGAAACTTAGCTCATATAGGTCCATCCGTTGATTTAGCTATTTTTTCACTCCACTTAGCCGGTATCTCATCTATCCTTGGAGCTATTAATTTTATTACA
ACTATTATTAATATACGATGAGAAGGAATACTAATAGAACGACTCCCATTATTTGTATGATCAGTATTTATTACTGCAATTTTATTATTACTTTCTCTACCAG
TTCTTGCTGGAGCAATTACAATACTTTTAACTGATCGAAATTTTAATACCACTTTTTTTGATCCGA
>PLO360 COI
AGGTACTTCTTTAAGTTTAATAATTCGAACAGAATTAGGTCAACCAGGATCTCTTCTCAATGATGATCAATTATATAATGTTATTGTTACAGCCCATGCATTT
GTAATAATCTTTTTTCTTGTTATACCTGTCATAATTGGAGGATTTGGCAACTGATTAGTTCCCTTAATATTGGGAGCCCCAGATATAGCATTCCCACGAATAA
ATAACATAAGTTTTTGACTTTTACCACCCTCTCTCACTTTATTATTATCCTCAGCCGCAGTAGAAAGAGGTGTAGGAACTGGATGAACCGTATATCCCCCTCT
CTCAAGAAACTTAGCTCATATAGGTCCATCCGTTGATTTAGCTATTTTTTCACTCCACTTAGCCGGTATCTCATCTATCCTTGGAGCTATTAATTTTATTACA
ACTATTATTAATATACGATGAGAAGGAATACTAATAGAACGACTCCCATTATTTGTATGATCAGTATTTATTACTGCAATTTTATTATTACTTTCTCTACCAG
TTCTTGCTGGAGCAATTACAATACTTTTAACTGATCGAAATTTTAATACCACTTTTTTTGATCCGA
>PLO359 COI
AGGTACTTCTTTAAGTTTAATAATTCGAACAGAATTAGGTCAACCAGGATCTCTTCTCAATGATGATCAATTATATAATGTTATTGTTACAGCCCATGCATTT
GTAATAATCTTTTTTCTTGTTATACCTGTCATAATTGGAGGATTTGGCAACTGATTAGTTCCCTTAATATTGGGAGCCCCAGATATAGCATTCCCACGAATAA
ATAACATAAGTTTTTGACTTTTACCACCCTCTCTCACTTTATTATTATCCTCAGCCGCAGTAGAAAGAGGTGTAGGAACTGGATGAACCGTATATCCCCCTCT
CTCAAGAAACTTAGCTCATATAGGTCCATCCGTTGATTTAGCTATTTTTTCACTCCACTTAGCCGGTATCTCATCTATCCTTGGAGCTATTAATTTTATTACA
ACTATTATTAATATACGATGAGAAGGAATACTAATAGAACGACTCCCATTATTTGTATGATCAGTATTTATTACTGCAATTTTATTATTACTTTCTCTACCAG
TTCTTGCTGGAGCAATTACAATACTTTTAACTGATCGAAATTTTAATACCACTTTTTTTGATCCGA
>PLO358 COI
AGGTACTTCTTTAAGTTTAATAATTCGAACAGAATTAGGTCAACCAGGATCTCTTCTCAATGATGATCAATTATATAATGTTATTGTTACAGCCCATGCATTT
GTAATAATCTTTTTTCTTGTTATACCTGTCATAATTGGAGGATTTGGCAACTGATTAGTTCCCTTAATATTGGGAGCCCCAGATATAGCATTCCCACGAATAA
ATAACATAAGTTTTTGACTTTTACCACCCTCTCTCACTTTATTATTATCCTCAGCCGCAGTAGAAAGAGGTGTAGGAACTGGATGAACCGTATATCCCCCTCT
CTCAAGAAACTTAGCTCATATAGGTCCATCCGTTGATTTAGCTATTTTTTCACTCCACTTAGCCGGTATCTCATCTATCCTTGGAGCTATTAATTTTATTACA
ACTATTATTAATATACGATGAGAAGGAATACTAATAGAACGACTCCCATTATTTGTATGATCAGTATTTATTACTGCAATTTTATTATTACTTTCTCTACCAG
TTCTTGCTGGAGCAATTACAATACTTTTAACTGATCGAAATTTTAATACCACTTTTTTTGATCCAA
>PLO357 COI
AGGTACTTCTTTAAGTTTAATAATTCGAACAGAATTAGGTCAACCAGGATCTCTTCTCAATGATGATCAATTATATAATGTTATTGTTACAGCCCATGCATTT
GTAATAATCTTTTTTCTTGTTATACCTGTCATAATTGGAGGATTTGGCAACTGATTAGTTCCCTTAATATTGGGAGCCCCAGATATAGCATTCCCACGAATAA
ATAACATAAGTTTTTGACTTTTACCACCCTCTCTCACTTTATTATTATCCTCAGCCGCAGTAGAAAGAGGTGTAGGAACTGGATGAACCGTATATCCCCCTCT
CTCAAGAAACTTAGCTCATATAGGTCCATCCGTTGATTTAGCTATTTTTTCACTCCACTTAGCCGGTATCTCATCTATCCTTGGAGCTATTAATTTTATTACA
ACTATTATTAATATACGATGAGAAGGAATACTAATAGAACGACTCCCATTATTTGTATGATCAGTATTTATTACTGCAATTTTATTATTACTTTCTCTACCAG
TTCTTGCTGGAGCAATTACAATACTTTTAACTGATCGAAATTTTAATACCACTTTTTTTGATCCGA
>PLO356 COI
AGGTACTTCTTTAAGTTTAATAATTCGAACAGAATTAGGTCAACCAGGATCTCTTCTCAATGATGATCAATTATATAATGTTATTGTTACAGCCCATGCATTT
GTAATAATCTTTTTTCTTGTTATACCTGTCATAATTGGAGGATTTGGCAACTGATTAGTTCCCTTAATATTGGGAGCCCCAGATATAGCATTCCCACGAATAA
ATAACATAAGTTTTTGACTTTTACCACCCTCTCTCACTTTATTATTATCCTCAGCCGCAGTAGAAAGAGGTGTAGGAACTGGATGAACCGTATATCCCCCTCT
CTCAAGAAACTTAGCTCATATAGGTCCATCCGTTGATTTAGCTATTTTTTCACTCCACTTAGCCGGTATCTCATCTATCCTTGGAGCTATTAATTTTATTACA
ACTATTATTAATATACGATGAGAAGGAATACTAATAGAACGACTCCCATTATTTGTATGATCAGTATTTATTACTGCAATTTTATTATTACTTTCTCTACCAG
TTCTTGCTGGAGCAATTACAATACTTTTAACTGATCGAAATTTTAATACCACTTTTTTTGATCCGA
>PLI450 COI
AGGTACCTCTCTAAGTTTAATAATTCGAACAGAATTAGGTCAACCAGGATCTCTCCTAAATGATGATCAACTATATAACGTAATTGTTACAGCACATGCATTT
GTAATAATTTTTTTTCTTGTTATACCTGTTATAATTGGAGGATTTGGTAATTGATTAGTTCCCTTAATACTAGGAGCTCCAGACATAGCATTCCCACGAATAA
ACAATATAAGTTTTTGACTTTTACCACCTTCTCTTACCCTATTATTATCTTCAGCTGCAGTAGAAAGAGGTGCAGGTACTGGATGGACTGTATATCCCCCTCT
TTCAAGAAATTTAGCTCATATAGGCCCTTCCGTTGATCTAGCTATTTTTTCTCTTCATTTAGCTGGTATTTCATCTATTCTTGGAGCCATTAACTTCATTACA
ACTATTATCAATATACGATGAGAAGGAATATTAATAGAACGACTTCCATTATTTGTATGATCAGTATTTATTACCGCAATTTTATTACTTCTTTCCTTACCAG

TCCTTGCTGGAGCAATTACAATACTCTTAACAGATCGAAATTTTAATACCACTTTTTTTGACCCAA
>PLI449 COI
AGGTACCTCTCTAAGTTTAATAATTCGAACAGAATTAGGTCAACCAGGATCTCTCCTAAATGATGATCAACTATATAACGTAATTGTTACAGCACATGCATTT
GTAATAATTTTTTTTCTTGTTATACCTGTTATAATTGGAGGATTTGGTAATTGATTAGTTCCCTTAATACTAGGAGCTCCAGACATAGCATTCCCACGAATAA
ACAATATAAGTTTTTGACTTTTACCACCTTCTCTTACCCTATTATTATCTTCAGCTGCAGTAGAAAGAGGTGCAGGTACTGGATGGACTGTATATCCCCCTCT
TTCAAGAAATTTAGCTCATATAGGCCCTTCCGTTGATCTAGCTATTTTTTCTCTTCATTTAGCTGGTATTTCATCTATTCTTGGAGCCATTAACTTCATTACA
ACTATTATCAATATACGATGAGAAGGAATATTAATAGAACGACTTCCATTATTTGTATGATCAGTATTTATTACCGCAATTTTATTACTTCTTTCCTTACCAG
TCCTTGCTGGAGCAATTACAATACTCTTAACAGATCGAAATTTTAATACCACTTTTTTTGACCCAA
>PLI448 COI
AGGTACCTCTCTAAGTTTAATAATTCGAACAGAATTAGGTCAACCAGGATCTCTCCTAAATGATGATCAACTATATAACGTAATTGTTACAGCACATGCATTT
GTAATAATTTTTTTTCTTGTTATACCTGTTATAATTGGAGGATTTGGTAATTGATTAGTTCCCTTAATACTAGGAGCTCCAGACATAGCATTCCCACGAATAA
ACAATATAAGTTTTTGACTTTTACCACCTTCTCTTACCCTATTATTATCTTCAGCTGCAGTAGAAAGAGGTGCAGGTACTGGATGGACTGTATATCCCCCTCT
TTCAAGAAATTTAGCTCATATAGGCCCTTCCGTTGACCTAGCTATTTTTTCTCTTCATTTAGCTGGTATTTCATCTATTCTTGGAGCCATTAACTTCATTACA
ACTATTATCAATATACGATGAGAAGGAATATTAATAGAACGACTTCCATTATTTGTATGATCAGTATTTATTACCGCAATTTTATTACTTCTTTCCTTACCAG
TCCTTGCTGGAGCAATTACAATACTCTTAACAGATCGAAATTTTAATACCACTTTTTTTGACCCAA
>PLI447 COI
AGGTACCTCTCTAAGTTTAATAATTCGAACAGAATTAGGTCAACCAGGATCTCTCCTAAATGATGATCAACTATATAACGTAATTGTTACAGCACATGCATTT
GTAATAATTTTTTTTCTTGTTATACCTGTTATAATTGGAGGATTTGGTAATTGATTAGTTCCCTTAATACTAGGAGCTCCAGACATAGCATTCCCACGAATAA
ACAATATAAGTTTTTGACTTTTACCACCTTCTCTTACCCTATTATTATCTTCAGCTGCAGTAGAAAGAGGTGCAGGTACTGGATGGACTGTATATCCCCCTCT
TTCAAGAAATTTAGCTCATATAGGCCCTTCCGTTGATCTAGCTATTTTTTCTCTTCATTTAGCTGGTATTTCATCTATTCTTGGAGCCATTAACTTCATTACA
ACTATTATCAATATACGATGAGAAGGAATATTAATAGAACGACTTCCATTATTTGTATGATCAGTATTTATTACCGCAATTTTATTACTTCTTTCCTTACCAG
TCCTTGCTGGAGCAATTACAATACTCTTAACAGATCGAAATTTTAATACCACTTTTTTTGACCCAA
>PLI446 COI
AGGTACCTCTCTAAGTTTAATAATTCGAACAGAATTAGGTCAACCAGGATCTCTCCTAAATGATGATCAACTATATAACGTAATTGTTACAGCACATGCATTT
GTAATAATTTTTTTTCTTGTTATACCTGTTATAATTGGAGGATTTGGTAATTGATTAGTTCCCTTAATACTAGGAGCTCCAGACATAGCATTCCCACGAATAA
ACAATATAAGTTTTTGACTTTTACCACCTTCTCTTACCCTATTATTATCTTCAGCTGCAGTAGAAAGAGGTGCAGGTACTGGATGGACTGTATATCCCCCTCT
TTCAAGAAATTTAGCTCATATAGGCCCTTCCGTTGATCTAGCTATTTTTTCTCTTCATTTAGCTGGTATTTCATCTATTCTTGGAGCCATTAACTTCATTACA
ACTATTATCAATATACGATGAGAAGGAATATTAATAGAACGACTTCCATTATTTGTATGATCAGTATTTATTACCGCAATTTTATTACTTCTTTCCTTACCAG
TCCTTGCTGGAGCAATTACAATACTCTTAACAGATCGAAATTTTAATACCACTTTTTTTGACCCAA
>PLI444 COI
AGGTACCTCTCTAAGTTTAATAATTCGAACAGAATTAGGTCAACCAGGATCTCTCCTAAATGATGATCAACTATATAACGTAATTGTTACAGCACATGCATTT
GTAATAATTTTTTTTCTTGTTATACCTGTTATAATTGGAGGATTTGGTAATTGATTAGTTCCCTTAATACTAGGAGCTCCAGACATAGCATTCCCACGAATAA
ACAATATAAGTTTTTGACTTTTACCACCTTCTCTTACCCTATTATTATCTTCAGCTGCAGTAGAAAGAGGTGCAGGTACTGGATGGACTGTATATCCCCCTCT
TTCAAGAAATTTAGCTCATATAGGCCCTTCCGTTGATCTAGCTATTTTTTCTCTTCATTTAGCTGGTATTTCATCTATTCTTGGAGCCATTAACTTCATTACA
ACTATTATCAATATACGATGAGAAGGAATATTAATAGAACGACTTCCATTATTTGTATGATCAGTATTTATTACCGCAATTTTATTACTTCTTTCCTTACCAG
TCCTTGCTGGAGCAATTACAATACTCTTAACAGATCGAAATTTTAATACCACTTTTTTTGACCCAA
>PLI443 COI
AGGTACCTCTCTAAGTTTAATAATTCGAACAGAATTAGGTCAACCAGGATCTCTCCTAAATGATGATCAACTATATAACGTAATTGTTACAGCACATGCATTT
GTAATAATTTTTTTTCTTGTTATACCTGTTATAATTGGAGGATTTGGTAATTGATTAGTTCCCTTAATACTAGGAGCTCCAGACATAGCATTCCCACGAATAA
ACAATATAAGTTTTTGACTTTTACCACCTTCTCTTACCCTATTATTATCTTCAGCTGCAGTAGAAAGAGGTGCAGGTACTGGATGGACTGTATATCCCCCTCT
TTCAAGAAATTTAGCTCATATAGGCCCTTCCGTTGACCTAGCTATTTTTTCTCTTCATTTAGCTGGTATTTCATCTATTCTTGGAGCCATTAACTTCATTACA
ACTATTATCAATATACGATGAGAAGGAATATTAATAGAACGACTTCCATTATTTGTATGATCAGTATTTATTACCGCAATTTTATTACTTCTTTCCTTACCAG
TCCTTGCTGGAGCAATTACAATACTCTTAACAGATCGAAATTTTAATACCACTTTTTTTGACCCAA
>MAL126 COI
AGGTACTTCTTTAAGTTTAATAATTCGAACAGAATTAGGTCAACCAGGATCTCTTCTCAATGATGATCAATTATATAATGTTATTGTTACAGCCCATGCATTT
GTAATAATCTTTTTTCTTGTTATACCTGTCATAATTGGAGGATTTGGCAACTGATTAGTTCCCTTAATATTGGGAGCCCCAGATATAGCATTCCCACGAATAA
ATAACATAAGTTTTTGACTTTTACCACCCTCTCTCACTTTATTATTATCCTCAGCCGCAGTAGAAAGAGGTGTAGGAACTGGATGAACCGTATATCCCCCTCT
CTCAAGAAACTTAGCTCATATAGGTCCATCCGTTGATTTAGCTATTTTTTCACTCCACTTAGCCGGTATCTCATCTATCCTTGGAGCTATTAATTTTATTACA
ACTATTATTAATATACGATGAGAAGGAATACTAATAGAACGACTCCCATTATTTGTATGATCAGTATTTATTACTGCAATTTTATTATTACTTTCTCTACCAG
TTCTTGCTGGAGCAATTACAATACTTTTAACTGATCGAAATTTTAATACCACTTTTTTTGATCCGA
>MAL125 COI
AGGTACTTCTTTAAGTTTAATAATTCGAACAGAATTAGGTCAACCAGGATCTCTTCTCAATGATGATCAATTATATAATGTTATTGTTACAGCCCATGCATTT
GTAATAATCTTTTTTCTTGTTATACCTGTCATAATTGGAGGATTTGGCAACTGATTAGTTCCCTTAATATTGGGAGCCCCAGATATAGCATTCCCACGAATAA
ATAACATAAGTTTTTGACTTTTACCACCCTCTCTCACTTTATTATTATCCTCAGCCGCAGTAGAAAGAGGTGTAGGAACTGGATGAACCGTATATCCCCCTCT
CTCAAGAAACTTAGCTCATATAGGTCCATCCGTTGATTTAGCTATTTTTTCACTCCACTTAGCCGGTATCTCATCTATCCTTGGAGCTATTAATTTTATTACA
ACTATTATTAATATACGATGAGAAGGAATACTAATAGAACGACTCCCATTATTTGTATGATCAGTATTTATTACTGCAATTTTATTATTACTTTCTCTACCAG
TTCTTGCTGGAGCAATTACAATACTTTTAACTGATCGAAATTTTAATACCACTTTTTTTGATCCGA
>MAL124 COI
AGGTACTTCTTTAAGTTTAATAATTCGAACAGAATTAGGTCAACCAGGATCTCTTCTCAATGATGATCAATTATATAATGTTATTGTTACAGCCCATGCATTT
GTAATAATCTTTTTTCTTGTTATACCTGTCATAATTGGAGGATTTGGCAACTGATTAGTTCCCTTAATATTGGGAGCCCCAGATATAGCATTCCCACGAATAA
ATAACATAAGTTTTTGACTTTTACCACCCTCTCTCACTTTATTATTATCCTCAGCCGCAGTAGAAAGAGGTGTAGGAACTGGATGAACCGTATATCCCCCTCT
CTCAAGAAACTTAGCTCATATAGGTCCATCCGTTGATTTAGCTATTTTTTCACTCCACTTAGCCGGTATCTCATCTATCCTTGGAGCTATTAATTTTATTACA
ACTATTATTAATATACGATGAGAAGGAATACTAATAGAACGACTCCCATTATTTGTATGATCAGTATTTATTACTGCAATTTTATTATTACTTTCTCTACCAG
TTCTTGCTGGAGCAATTACAATACTTTTAACTGATCGAAATTTTAATACCACTTTTTTTGATCCGA
>MAL123 COI
AGGTACTTCTTTAAGTTTAATAATTCGAACAGAATTAGGTCAACCAGGATCTCTTCTCAATGATGATCAATTATATAATGTTATTGTTACAGCCCATGCATTT
GTAATAATCTTTTTTCTTGTTATACCTGTCATAATTGGAGGATTTGGCAACTGATTAGTTCCCTTAATATTGGGAGCCCCAGATATAGCATTCCCACGAATAA
ATAACATAAGTTTTTGACTTTTACCACCCTCTCTCACTTTATTATTATCCTCAGCCGCAGTAGAAAGAGGTGTAGGAACTGGATGAACCGTATATCCCCCTCT
CTCAAGAAACTTAGCTCATATAGGTCCATCCGTTGATTTAGCTATTTTTTCACTCCACTTAGCCGGTATCTCATCTATCCTTGGAGCTATTAATTTTATTACA
ACTATTATTAATATACGATGAGAAGGAATACTAATAGAACGACTCCCATTATTTGTATGATCAGTATTTATTACTGCAATTTTATTATTACTTTCTCTACCAG
TTCTTGCTGGAGCAATTACAATACTTTTAACTGATCGAAATTTTAATACCACTTTTTTTGATCCGA
>MAL121 COI
AGGTACTTCTTTAAGTTTAATAATTCGAACAGAATTAGGTCAACCAGGATCTCTTCTCAATGATGATCAATTATATAATGTTATTGTTACAGCCCATGCATTT
GTAATAATCTTTTTTCTTGTTATACCTGTCATAATTGGAGGATTTGGCAACTGATTAGTTCCCTTAATATTGGGAGCCCCAGATATAGCATTCCCACGAATAA
ATAACATAAGTTTTTGACTTTTACCACCCTCTCTCACTTTATTATTATCCTCAGCCGCAGTAGAAAGAGGTGTAGGAACTGGATGAACCGTATATCCCCCTCT
CTCAAGAAACTTAGCTCATATAGGTCCATCCGTTGATTTAGCTATTTTTTCACTCCACTTAGCCGGTATCTCATCTATCCTTGGAGCTATTAATTTTATTACA
ACTATTATTAATATACGATGAGAAGGAATACTAATAGAACGACTCCCATTATTTGTATGATCAGTATTTATTACTGCAATTTTATTATTACTTTCTCTACCAG
TTCTTGCTGGAGCAATTACAATACTTTTAACTGATCGAAATTTTAATACCACTTTTTTTGATCCGA
>MAL120 COI

AGGTACTTCTTTAAGTTTAATAATTCGAACAGAATTAGGTCAACCAGGATCTCTTCTCAATGATGATCAATTATATAATGTTATTGTTACAGCCCATGCATTT
GTAATAATCTTTTTTCTTGTTATACCTGTCATAATTGGAGGATTTGGCAACTGATTAGTTCCCTTAATATTGGGAGCCCCAGATATAGCATTCCCACGAATAA
ATAACATAAGTTTTTGACTTTTACCACCCTCTCTCACTTTATTATTATCCTCAGCCGCAGTAGAAAGAGGTGTAGGAACTGGATGAACCGTATATCCCCCTCT
CTCAAGAAACTTAGCTCATATAGGTCCATCCGTTGATTTAGCTATTTTTTCACTCCACTTAGCCGGTATCTCATCTATCCTTGGAGCTATTAATTTTATTACA
ACTATTATTAATATACGATGAGAAGGAATACTAATAGAACGACTCCCATTATTTGTATGATCAGTATTTATTACTGCAATTTTATTATTACTTTCTCTACCAG
TTCTTGCTGGAGCAATTACAATACTTTTAACTGATCGAAATTTTAATACCACTTTTTTTGATCCGA
>MAL119 COI
AGGTACTTCTTTAAGTTTAATAATTCGAACAGAATTAGGTCAACCAGGATCTCTTCTCAATGATGATCAATTATATAATGTTATTGTTACAGCCCATGCATTT
GTAATAATCTTTTTTCTTGTTATACCTGTCATAATTGGAGGATTTGGCAACTGATTAGTTCCCTTAATATTGGGAGCCCCAGATATAGCATTCCCACGAATAA
ATAACATAAGTTTTTGACTTTTACCACCCTCTCTCACTTTATTATTATCCTCAGCCGCAGTAGAAAGAGGTGTAGGAACTGGATGAACCGTATATCCCCCTCT
CTCAAGAAACTTAGCTCATATAGGTCCATCCGTTGATTTAGCTATTTTTTCACTCCACTTAGCCGGTATCTCATCTATCCTTGGAGCTATTAATTTTATTACA
ACTATTATTAATATACGATGAGAAGGAATACTAATAGAACGACTCCCATTATTTGTATGATCAGTATTTATTACTGCAATTTTATTATTACTTTCTCTACCAG
TTCTTGCTGGAGCAATTACAATACTTTTAACTGATCGAAATTTTAATACCACTTTTTTTGATCCGA
>ISLG490 COI
AGGTACTTCTTTAAGTTTAATAATTCGAACAGAATTAGGTCAACCAGGATCTCTTCTCAATGATGATCAATTATATAATGTTATTGTTACAGCCCATGCATTT
GTAATAATCTTTTTTCTTGTTATACCTGTCATAATTGGAGGATTTGGCAACTGATTAGTTCCCTTAATATTGGGAGCCCCAGATATAGCATTCCCACGAATAA
ATAACATAAGTTTTTGACTTTTACCACCCTCTCTCACTTTATTATTATCCTCAGCCGCAGTAGAAAGAGGTGTAGGAACTGGATGAACCGTATATCCCCCTCT
CTCAAGAAACTTAGCTCATATAGGTCCATCCGTTGATTTAGCTATTTTTTCACTCCACTTAGCCGGTATCTCATCTATCCTTGGAGCTATTAATTTTATTACA
ACTATTATTAATATACGATGAGAAGGAATACTAATAGAACGACTCCCATTATTTGTATGATCAGTATTTATTACTGCAATTTTATTATTACTTTCTCTACCAG
TTCTTGCTGGAGCAATTACAATACTTTTAACTGATCGAAATTTTAATACCACTTTTTTTGATCCGA
>ISLG489 COI
AGGTACTTCTTTAAGTTTAATAATTCGAACAGAATTAGGTCAACCAGGATCTCTTCTCAATGATGATCAATTATATAATGTTATTGTTACAGCCCATGCATTT
GTAATAATCTTTTTTCTTGTTATACCTGTCATAATTGGAGGATTTGGCAACTGATTAGTTCCCTTAATATTGGGAGCCCCAGATATAGCATTCCCACGAATAA
ATAACATAAGTTTTTGACTTTTACCACCCTCTCTCACTTTATTATTATCCTCAGCCGCAGTAGAAAGAGGTGTAGGAACTGGATGAACCGTATATCCCCCTCT
CTCAAGAAACTTAGCTCATATAGGTCCATCCGTTGATTTAGCTATTTTTTCACTCCACTTAGCCGGTATCTCATCTATCCTTGGAGCTATTAATTTTATTACA
ACTATTATTAATATACGATGAGAAGGAATACTAATAGAACGACTCCCATTATTTGTATGATCAGTATTTATTACTGCAATTTTATTATTACTTTCTCTACCAG
TTCTTGCTGGAGCAATTACAATACTTTTAACTGATCGAAATTTTAATACCACTTTTTTTGATCCGA
>ISLG487 CO
AGGTACTTCTTTAAGTTTAATAATTCGAACAGAATTAGGTCAACCAGGATCTCTTCTCAATGATGATCAATTATATAATGTTATTGTTACAGCCCATGCATTT
GTAATAATCTTTTTTCTTGTTATACCTGTCATAATTGGAGGATTTGGCAACTGATTAGTTCCCTTAATATTGGGAGCCCCAGATATAGCATTCCCACGAATAA
ATAACATAAGTTTTTGACTTTTACCACCCTCTCTCACTTTATTATTATCCTCAGCCGCAGTAGAAAGAGGTGTAGGAACTGGATGAACCGTATATCCCCCTCT
CTCAAGAAACTTAGCTCATATAGGTCCATCCGTTGATTTAGCTATTTTTTCACTCCACTTAGCCGGTATCTCATCTATCCTTGGAGCTATTAATTTTATTACA
ACTATTATTAATATACGATGAGAAGGAATACTAATAGAACGACTCCCATTATTTGTATGATCAGTATTTATTACTGCAATTTTATTATTACTTTCTCTACCAG
TTCTTGCTGGAGCAATTACAATACTTTTAACTGATCGAAATTTTAATACCACTTTTTTTGATCCGA
>ISLG486 COI
AGGTACTTCTTTAAGTTTAATAATTCGAACAGAATTAGGTCAACCAGGATCTCTTCTCAATGATGATCAATTATATAATGTTATTGTTACAGCCCATGCATTT
GTAATAATCTTTTTTCTTGTTATACCTGTCATAATTGGAGGATTTGGCAACTGATTAGTTCCCTTAATATTGGGAGCCCCAGATATAGCATTCCCACGAATAA
ATAACATAAGTTTTTGACTTTTACCACCCTCTCTCACTTTATTATTATCCTCAGCCGCAGTAGAAAGAGGTGTAGGAACTGGATGAACCGTATATCCCCCTCT
CTCAAGAAACTTAGCTCATATAGGTCCATCCGTTGATTTAGCTATTTTTTCACTCCACTTAGCCGGTATCTCATCTATCCTTGGAGCTATTAATTTTATTACA
ACTATTATTAATATACGATGAGAAGGAATACTAATAGAACGACTCCCATTATTTGTATGATCAGTATTTATTACTGCAATTTTATTATTACTTTCTCTACCAG
TTCTTGCTGGAGCAATTACAATACTTTTAACTGATCGAAATTTTAATACCACTTTTTTTGATCCGA
>ISLG485 COI
AGGTACTTCTTTAAGTTTAATAATTCGAACAGAATTAGGTCAACCAGGATCTCTTCTCAATGATGATCAATTATATAATGTTATTGTTACAGCCCATGCATTT
GTAATAATCTTTTTTCTTGTTATACCTGTCATAATTGGAGGATTTGGCAACTGATTAGTTCCCTTAATATTGGGAGCCCCAGATATAGCATTCCCACGAATAA
ATAACATAAGTTTTTGACTTTTACCACCCTCTCTCACTTTATTATTATCCTCAGCCGCAGTAGAAAGAGGTGTAGGAACTGGATGAACCGTATATCCCCCTCT
CTCAAGAAACTTAGCTCATATAGGTCCATCCGTTGATTTAGCTATTTTTTCACTCCACTTAGCCGGTATCTCATCTATCCTTGGAGCTATTAATTTTATTACA
ACTATTATTAATATACGATGAGAAGGAATACTAATAGAACGACTCCCATTATTTGTATGATCAGTATTTATTACTGCAATTTTATTATTACTTTCTCTACCAG
TTCTTGCTGGAGCAATTACAATACTTTTAACTGATCGAAATTTTAATACCACTTTTTTTGATCCGA
>ISLG484 COI
AGGTACTTCTTTAAGTTTAATAATTCGAACAGAATTAGGTCAACCAGGATCTCTTCTCAATGATGATCAATTATATAATGTTATTGTTACAGCCCATGCATTT
GTAATAATCTTTTTTCTTGTTATACCTGTCATAATTGGAGGATTTGGCAACTGATTAGTTCCCTTAATATTGGGAGCCCCAGATATAGCATTCCCACGAATAA
ATAACATAAGTTTTTGACTTTTACCACCCTCTCTCACTTTATTATTATCCTCAGCCGCAGTAGAAAGAGGTGTAGGAACTGGATGAACCGTATATCCCCCTCT
CTCAAGAAACTTAGCTCATATAGGTCCATCCGTTGATTTAGCTATTTTTTCACTCCACTTAGCCGGTATCTCATCTATCCTTGGAGCTATTAATTTTATTACA
ACTATTATTAATATACGATGAGAAGGAATACTAATAGAACGACTCCCATTATTTGTATGATCAGTATTTATTACTGCAATTTTATTATTACTTTCTCTACCAG
TTCTTGCTGGAGCAATTACAATACTTTTAACTGATCGAAATTTTAATACCACTTTTTTTGATCCGA
>ISLG483 COI
AGGTACTTCTTTAAGTTTAATAATTCGAACAGAATTAGGTCAACCAGGATCTCTTCTCAATGATGATCAATTATATAATGTTATTGTTACAGCCCATGCATTT
GTAATAATCTTTTTTCTTGTTATACCTGTCATAATTGGAGGATTTGGCAACTGATTAGTTCCCTTAATATTGGGAGCCCCAGATATAGCATTCCCACGAATAA
ATAACATAAGTTTTTGACTTTTACCACCCTCTCTCACTTTATTATTATCCTCAGCCGCAGTAGAAAGAGGTGTAGGAACTGGATGAACCGTATATCCCCCTCT
CTCAAGAAACTTAGCTCATATAGGTCCATCCGTTGATTTAGCTATTTTTTCACTCCACTTAGCCGGTATCTCATCTATCCTTGGAGCTATTAATTTTATTACA
ACTATTATTAATATACGATGAGAAGGAATACTAATAGAACGACTCCCATTATTTGTATGATCAGTATTTATTACTGCAATTTTATTATTACTTTCTCTACCAG
TTCTTGCTGGAGCAATTACAATACTTTTAACTGATCGAAATTTTAATACCACTTTTTTTGATCCGA
>ISL427 COI
AGGTACCTCTCTAAGTTTAATAATTCGAACAGAATTAGGTCAACCAGGATCTCTCCTAAATGATGATCAACTATATAACGTAATTGTTACAGCACATGCATTT
GTAATAATTTTTTTTCTTGTTATACCTGTTATAATTGGAGGATTTGGTAATTGATTAGTTCCCTTAATACTAGGAGCTCCAGACATAGCATTCCCACGAATAA
ACAATATAAGTTTTTGACTTTTACCACCTTCTCTTACCCTATTATTATCTTCAGCTGCAGTAGAAAGAGGTGCAGGTACTGGATGGACTGTATATCCCCCTCT
TTCAAGAAATTTAGCTCATATAGGCCCTTCCGTTGATCTAGCTATTTTTTCTCTTCATTTAGCTGGTATTTCATCTATTCTTGGAGCCATTAACTTCATTACA
ACTATTATCAATATACGATGAGAAGGAATATTAATAGAACGACTTCCATTATTTGTATGATCAGTATTTATTACCGCAATTTTATTACTTCTTTCCTTACCAG
TCCTTGCTGGAGCAATTACAATACTCTTAACAGATCGAAATTTTAATACCACTTTTTTTGACCCAA
>ISL426 COI
AGGTACCTCTCTAAGTTTAATAATTCGAACAGAATTAGGTCAACCAGGATCTCTCCTAAATGATGATCAACTATATAACGTAATTGTTACAGCACATGCATTT
GTAATAATTTTTTTTCTTGTTATACCTGTTATAATTGGAGGATTTGGTAATTGATTAGTTCCCTTAATACTAGGAGCTCCAGACATAGCATTCCCACGAATAA
ACAATATAAGTTTTTGACTTTTACCACCTTCTCTTACCCTATTATTATCTTCAGCTGCAGTAGAAAGAGGTGCAGGTACTGGATGGACTGTATATCCCCCTCT
TTCAAGAAATTTAGCTCATATAGGTCCTTCCGTTGATCTAGCTATTTTTTCTCTTCATTTAGCTGGTATTTCATCTATTCTTGGAGCCATTAACTTCATTACA
ACTATTATCAATATACGATGAGAAGGAATATTAATAGAACGACTTCCATTATTTGTATGATCAGTATTTATTACCGCAATTTTATTACTTCTTTCCTTACCAG
TCCTTGCTGGAGCAATTACAATACTCTTAACAGATCGAAATTTTAATACCACTTTTTTTGACCCAG
>ISL425 COI
AGGTACCTCTCTAAGTTTAATAATTCGAACAGAATTAGGTCAACCAGGATCTCTCCTAAATGATGATCAACTATATAACGTAATTGTTACAGCACATGCATTT
GTAATAATTTTTTTTCTTGTTATACCTGTTATAATTGGAGGATTTGGTAATTGATTAGTTCCCTTAATACTAGGAGCTCCAGACATAGCATTCCCACGAATAA

```
ACAATATAAGTTTTTGACTTTTACCACCTTCTCTTACCCTATTATTATCTTCAGCTGCAGTAGAAAGAGGTGCAGGTACTGGATGGACTGTATATCCCCCTCT
TTCAAGAAATTTAGCTCATATAGGCCCTTCCGTTGATCTAGCTATTTTTTCTCTTCATTTAGCTGGTATTTCATCTATTCTTGGAGCCATTAACTTCATTACA
ACTATTATCAATATACGATGAGAAGGAATATTAATAGAACGACTTCCATTATTTGTATGATCAGTATTTATTACCGCAATTTTATTACTTCTTTCCTTACCAG
TCCTTGCTGGAGCAATTACAATACTCTTAACAGATCGAAATTTTAATACCACTTTTTTTGACCCAA
>ISL424 COI
AGGTACCTCTCTAAGTTTAATAATTCGAACAGAATTAGGTCAACCAGGATCTCTCCTAAATGATGATCAACTATATAACGTAATTGTTACAGCACATGCATTT
GTAATAATTTTTTTTCTTGTTATACCTGTTATAATTGGAGGATTTGGTAATTGATTAGTTCCCTTAATACTAGGAGCTCCAGACATAGCATTCCCACGAATAA
ACAATATAAGTTTTTGACTTTTACCACCTTCTCTTACCCTATTATTATCTTCAGCTGCAGTAGAAAGAGGTGCAGGTACTGGATGGACTGTATATCCCCCTCT
TTCAAGAAATTTAGCTCATATAGGCCCTTCCGTTGACCTAGCTATTTTTTCTCTTCATTTAGCTGGTATTTCATCTATTCTTGGAGCCATTAACTTCATTACA
ACTATTATCAATATACGATGAGAAGGAATATTAATAGAACGACTTCCATTATTTGTATGATCAGTATTTATTACCGCAATTTTATTACTTCTTTCCTTACCAG
TCCTTGCTGGAGCAATTACAATACTCTTAACAGATCGAAATTTTAATACCACTTTTTTTGACCCAA
>ISL423 COI
AGGTACCTCTCTAAGTTTAATAATTCGAACAGAATTAGGTCAACCAGGATCTCTCCTAAATGATGATCAACTATATAACGTAATTGTTACAGCACATGCATTT
GTAATAATTTTTTTTCTTGTTATACCTGTTATAATTGGAGGATTTGGTAATTGATTAGTTCCCTTAATACTAGGAGCTCCAGACATAGCATTCCCACGAATAA
ACAATATAAGTTTTTGACTTTTACCACCTTCTCTTACCCTATTATTATCTTCAGCTGCAGTAGAAAGAGGTGCAGGTACTGGATGGACTGTATATCCCCCTCT
TTCAAGAAATTTAGCTCATATAGGCCCTTCCGTTGATCTAGCTATTTTTTCTCTTCATTTAGCTGGTATTTCATCTATTCTTGGAGCCATTAACTTCATTACA
ACTATTATCAATATACGATGAGAAGGAATATTAATAGAACGACTTCCATTATTTGTATGATCAGTATTTATTACCGCAATTTTATTACTTCTTTCCTTACCAG
TCCTTGCTGGAGCAATTACAATACTCTTAACAGATCGAAATTTTAATACCACTTTTTTTGACCCAA
>ISL422 COI
AGGTACCTCTCTAAGTTTAATAATTCGAACAGAATTAGGTCAACCAGGATCTCTCCTAAATGATGATCAACTATATAACGTAATTGTTACAGCACATGCATTT
GTAATAATTTTTTTTCTTGTTATACCTGTTATAATTGGAGGATTTGGTAATTGATTAGTTCCCTTAATACTAGGAGCTCCAGATATAGCATTCCCACGAATAA
ACAATATAAGTTTTTGACTTTTACCACCTTCTCTTACCCTATTATTATCTTCAGCTGCAGTAGAAAGAGGTGCAGGTACTGGATGGACTGTATATCCCCCTCT
TTCAAGAAATTTAGCTCATATAGGCCCTTCCGTTGATCTAGCTATTTTTTCTCTTCATTTAGCTGGTATTTCATCTATTCTTGGAGCCATTAACTTCATTACA
ACTATTATCAATATACGATGAGAAGGAATATTAATAGAACGACTTCCATTATTTGTATGATCAGTATTTATTACCGCAATTTTATTACTTCTTTCCTTACCAG
TCCTTGCTGGAGCAATTACAATACTCTTAACAGATCGAAATTTTAATACCACTTTTTTTGACCCAA
>ISL421 COI
AGGTACCTCTCTAAGTTTAATAATTCGAACAGAATTAGGTCAACCAGGATCTCTCCTAAATGATGATCAACTATATAACGTAATTGTTACAGCACATGCATTT
GTAATAATTTTTTTTCTTGTTATACCTGTTATAATTGGAGGATTTGGTAATTGATTAGTTCCCTTAATACTAGGAGCTCCAGACATAGCATTCCCACGAATAA
ACAATATAAGTTTTTGACTTTTACCACCTTCTCTTACCCTATTATTATCTTCAGCTGCAGTAGAAAGAGGTGCAGGTACTGGATGGACTGTATATCCCCCTCT
TTCAAGAAATTTAGCTCATATAGGCCCTTCCGTTGATCTAGCTATTTTTTCTCTTCATTTAGCTGGTATTTCATCTATTCTTGGAGCCATTAACTTCATTACA
ACTATTATCAATATACGATGAGAAGGAATATTAATAGAACGACTTCCATTATTTGTATGATCAGTATTTATTACCGCAATTTTATTACTTCTTTCCTTACCAG
TCCTTGCTGGAGCAATTACAATACTCTTAACAGATCGAAATTTTAATACCACTTTTTTTGACCCAA
>ISL420 COI
AGGTACCTCTCTAAGTTTAATAATTCGAACAGAATTAGGTCAACCAGGATCTCTCCTAAATGATGATCAACTATATAACGTAATTGTTACAGCACATGCATTT
GTAATAATTTTTTTTCTTGTTATACCTGTTATAATTGGAGGATTTGGTAATTGATTAGTTCCCTTAATACTAGGAGCTCCAGACATAGCATTCCCACGAATAA
ACAATATAAGTTTTTGACTTTTACCACCTTCTCTTACCCTATTATTATCTTCAGCTGCAGTAGAAAGAGGTGCAGGTACTGGATGGACTGTATATCCCCCTCT
TTCAAGAAATTTAGCTCATATAGGCCCTTCCGTTGACCTAGCTATTTTTTCTCTTCATTTAGCTGGTATTTCATCTATTCTTGGAGCCATTAACTTCATTACA
ACTATTATCAATATACGATGAGAAGGAATATTAATAGAACGACTTCCATTATTTGTATGATCAGTATTTATTACCGCAATTTTATTACTTCTTTCCTTACCAG
TCCTTGCTGGAGCAATTACAATACTCTTAACAGATCGAAATTTTAATACCACTTTTTTTGACCCAA
>EE82 COI
GGGTACCTCTTTAAGTTTAATAATTCGAACAGAATTAGGTCAACCAGGATCTCTTCTCAATGATGATCAATTATATAATGTTATTGTCACAGCCCATGCATTT
GTAATAATCTTTTTCCTTGTTATGCCCGTTATAATTGGAGGATTCGGCAACTGATTAGTTCCCTTAATATTAGGAGCCCCAGACATAGCATTTCCACGAATAA
ATAATATAAGTTTTTGACTTCTACCACCCTCTCTTACTTTATTACTATCCTCAGCTGCAGTAGAAAGAGGTGTAGGAACCGGATGAACCGTATACCCTCCTCT
TTCAAGAAATTTAGCTCATATAGGTCCATCCGTTGATTTAGCTATTTTTTCACTACATCTAGCTGGTATCTCATCTATTCTTGGAGCTATTAATTTTATTACA
ACTATCATTAATATACGATGAGAAGGAATACTAATAGAACGACTCCCATTATTTGTATGATCAGTATTTATTACTGCAATTTTATTATTACTTTCCTTACCAG
TCCTTGCCGGAGCAATTACAATACTTTTAACTGATCGAAATTTTAATACCACTTTCTTTGACCCAA
>EE81 COI
GGGTACCTCTTTAAGTTTAATAATTCGAACAGAATTAGGTCAACCAGGATCTCTTCTCAATGATGATCAATTATATAATGTTATTGTCACAGCCCATGCATTT
GTAATAATCTTTTTCCTTGTTATGCCCGTTATAATTGGAGGATTCGGCAACTGATTAGTTCCCTTAATATTAGGAGCCCCAGACATAGCATTTCCACGAATAA
ATAATATAAGTTTTTGACTTCTACCACCCTCTCTTACTTTATTACTATCCTCAGCTGCAGTAGAAAGAGGTGTAGGAACCGGATGAACCGTATACCCTCCTCT
TTCAAGAAATTTAGCTCATATAGGTCCATCCGTTGATTTAGCTATTTTTTCACTACATCTAGCTGGTATCTCATCTATTCTTGGAGCTATTAATTTTATTACA
ACTATCATTAATATACGATGAGAAGGAATACTAATAGAACGACTCCCATTATTTGTATGATCAGTATTTATTACTGCAATTTTATTATTACTTTCCTTACCAG
TCCTTGCCGGAGCAATTACAATACTTTTAACTGATCGAAATTTTAATACCACTTTCTTTGACCCAA
>EE80 COI
GGGTACCTCTTTAAGTTTAATAATTCGAACAGAATTAGGTCAACCAGGATCTCTTCTCAATGATGATCAATTATATAATGTTATTGTCACAGCCCATGCATTT
GTAATAATCTTTTTCCTTGTTATGCCCGTTATAATTGGAGGATTCGGCAACTGATTAGTTCCCTTAATATTAGGAGCCCCAGACATAGCATTTCCACGAATAA
ATAATATAAGTTTTTGACTTCTACCACCCTCTCTTACTTTATTACTATCCTCAGCTGCAGTAGAAAGAGGTGTAGGAACCGGATGAACCGTATACCCTCCTCT
TTCAAGAAATTTAGCTCATATAGGTCCATCCGTTGATTTAGCTATTTTTTCGCTACATCTAGCTGGTATCTCATCTATTCTTGGAGCTATTAATTTTATTACA
ACTATCATTAATATACGATGAGAAGGAATACTAATAGAACGACTCCCATTATTTGTATGATCAGTATTTATTACTGCAATTTTATTATTACTTTCCTTACCAG
TCCTTGCCGGAGCAATTACAATACTTTTAACTGATCGAAATTTTAATACCACTTTCTTTGACCCAA
>EE79 COI
GGGTACCTCTTTAAGTTTAATAATTCGAACAGAATTAGGTCAACCAGGATCTCTTCTCAATGATGATCAATTATATAATGTTATTGTCACAGCCCATGCATTT
GTAATAATCTTTTTCCTTGTTATGCCCGTTATAATTGGAGGATTCGGCAACTGATTAGTTCCCTTAATATTAGGAGCCCCAGACATAGCATTTCCACGAATAA
ATAATATAAGTTTTTGACTTCTACCACCCTCTCTTACTTTATTACTATCCTCAGCTGCAGTAGAAAGAGGTGTAGGAACCGGATGAACCGTATACCCTCCTCT
TTCAAGAAATTTAGCTCATATAGGTCCATCCGTTGATTTAGCTATTTTTTCACTACATCTAGCTGGTATCTCATCTATTCTTGGAGCTATTAATTTTATTACA
ACTATCATTAATATACGATGAGAAGGAATACTAATAGAACGACTCCCATTATTTGTATGATCAGTATTTATTACTGCAATTTTATTATTACTTTCCTTACCAG
TCCTTGCCGGAGCAATTACAATACTTTTAACTGATCGAAATTTTAATACCACTTTCTTTGACCCAA
>EE77 COI
GGGTACCTCTTTAAGTTTAATAATTCGAACAGAATTAGGTCAACCAGGATCTCTTCTCAATGATGATCAATTATATAATGTTATTGTCACAGCCCATGCATTT
GTAATAATCTTTTTCCTTGTTATGCCCGTTATAATTGGAGGATTCGGCAACTGATTAGTTCCCTTAATATTAGGAGCCCCAGACATAGCATTTCCACGAATAA
ATAATATAAGTTTTTGACTTCTACCACCCTCTCTTACTTTATTACTATCCTCAGCTGCAGTAGAAAGAGGTGTAGGAACCGGATGAACCGTATACCCTCCTCT
TTCAAGAAATTTAGCTCATATAGGTCCATCCGTTGATTTAGCTATTTTTTCACTACATCTAGCTGGTATCTCATCTATTCTTGGAGCTATTAATTTTATTACA
ACTATCATTAATATACGATGAGAAGGAATACTAATAGAACGACTCCCATTATTTGTATGATCAGTATTTATTACTGCAATTTTATTATTACTTTCCTTACCAG
TCCTTGCCGGAGCAATTACAATACTTTTAACTGATCGAAATTTTAATACCACTTTCTTTGACCCAA
>EE76 COI
GGGTACCTCTTTAAGTTTAATAATTCGAACAGAATTAGGTCAACCAGGATCTCTTCTCAATGATGATCAATTATATAATGTTATTGTCACAGCCCATGCATTT
GTAATAATCTTTTTCCTTGTTATGCCCGTTATAATTGGAGGATTCGGCAACTGATTAGTTCCCTTAATATTAGGAGCCCCAGACATAGCATTTCCACGAATAA
ATAATATAAGTTTTTGACTTCTACCACCCTCTCTTACTTTATTACTATCCTCAGCTGCAGTAGAAAGAGGTGTAGGAACCGGATGAACCGTATACCCTCCTCT
TTCAAGAAATTTAGCTCATATAGGTCCATCCGTTGATTTAGCTATTTTTTCACTACATCTAGCTGGTATCTCATCTATTCTTGGAGCTATTAATTTTATTACA
```

ACTATCATTAATATACGATGAGAAGGAATACTAATAGAACGACTCCCATTATTTGTATGATCAGTATTTATTACTGCAATTTTATTATTACTTTCCTTACCAG
TCCTTGCCGGAGCAATTACAATACTTTTAACTGATCGAAATTTTAATACCACTTTCTTTGACCCAA
>EE75 COI
GGGTACCTCTTTAAGTTTAATAATTCGAACAGAATTAGGTCAACCAGGATCTCTTCTCAATGATGATCAATTATATAATGTTATTGTCACAGCCCATGCATTT
GTAATAATCTTTTTCCTTGTTATGCCCGTTATAATTGGAGGATTCGGCAACTGATTAGTTCCCTTAATATTAGGAGCCCCAGACATAGCATTTCCACGAATAA
ATAATATAAGTTTTTGACTTCTACCACCCTCTCTTACTTTATTACTATCCTCAGCTGCAGTAGAAAGAGGTGTAGGAACCGGATGAACCGTATACCCTCCTCT
TTCAAGAAATTTAGCTCATATAGGTCCATCCGTTGATTTAGCTATTTTTTCGCTACATCTAGCTGGTATTTCATCTATTCTTGGAGCTATTAATTTTATTACA
ACTATCATTAATATACGATGAGAAGGAATACTAATAGAACGACTCCCATTATTTGTATGATCAGTATTTATTACTGCAATTTTATTATTACTTTCCTTACCAG
TCCTTGCCGGAGCAATTACAATACTTTTAACTGATCGAAATTTTAATACCACTTTCTTTGACCCAA
>BM23 COI
GGGTACCTCTTTAAGTTTAATAATTCGAACAGAATTAGGTCAACCAGGATCTCTTCTCAATGATGATCAATTATATAATGTTATTGTCACAGCCCATGCATTT
GTAATAATCTTTTTCCTTGTTATGCCCGTTATAATTGGAGGATTCGGCAACTGATTAGTTCCCTTAATATTAGGAGCCCCAGACATAGCATTTCCACGAATAA
ATAATATAAGTTTTTGACTTCTACCACCCTCTCTTACTTTATTACTATCCTCAGCTGCAGTAGAAAGAGGTGTAGGAACTGGATGAACCGTATACCCTCCTCT
TTCAAGAAATTTAGCTCATATAGGTCCATCCGTTGATTTAGCTATTTTTTCACTACATCTAGCTGGTATCTCATCTATTCTTGGAGCTATTAATTTTATTACA
ACTATCATTAATATACGATGAGAAGGAATACTAATAGAACGACTCCCATTATTTGTATGATCAGTATTTATTACTGCAATTTTATTACTGCTTTCCTTACCAG
TCCTTGCCGGAGCAATTACAATACTTTTAACTGATCGAAATTTTAATACCACTTTCTTTGACCCAA
>BM22 COI
GGGTACCTCTTTAAGTTTAATAATTCGAACAGAATTAGGTCAACCAGGATCTCTTCTCAATGATGATCAATTATATAATGTTATTGTCACAGCCCATGCATTT
GTAATAATCTTTTTCCTTGTTATGCCCGTTATAATTGGAGGATTCGGCAACTGATTAGTTCCCTTAATATTAGGAGCCCCAGACATAGCATTTCCACGAATAA
ATAATATAAGTTTTTGACTTCTACCACCCTCTCTTACTTTATTACTATCCTCAGCTGCAGTAGAAAGAGGTGTAGGAACTGGATGAACCGTATACCCTCCTCT
TTCAAGAAATTTAGCTCATATAGGCCCATCCGTTGATTTAGCTATTTTTTCACTACATCTAGCTGGTATCTCATCTATTCTTGGAGCTATTAATTTTATTACA
ACTATCATTAATATACGATGAGAAGGAATACTAATAGAACGACTCCCATTATTTGTATGATCAGTATTTATTACTGCAATTTTATTACTGCTTTCCTTACCAG
TCCTTGCCGGAGCAATTACAATACTTTTAACTGATCGAAATTTTAATACCACTTTCTTTGACCCAA
>BM21 COI
GGGTACCTCTTTAAGTTTAATAATTCGAACAGAATTAGGTCAACCAGGATCTCTTCTCAATGATGATCAATTATATAATGTTATTGTCACAGCCCATGCATTT
GTAATAATCTTTTTCCTTGTTATGCCCGTTATAATTGGAGGATTCGGCAACTGATTAGTTCCCTTAATATTAGGAGCCCCAGACATAGCATTTCCACGAATAA
ATAATATAAGTTTTTGACTTCTACCACCCTCTCTTACTTTATTACTATCCTCAGCTGCAGTAGAAAGAGGTGTAGGAACTGGATGAACCGTATACCCTCCTCT
TTCAAGAAATTTAGCTCATATAGGTCCATCCGTTGATTTAGCTATTTTTTCACTACATCTAGCTGGTATCTCATCTATTCTTGGAGCTATTAATTTTATTACA
ACTATCATTAATATACGATGAGAAGGAATACTAATAGAACGACTCCCATTATTTGTATGATCAGTATTTATTACTGCAATTTTATTACTGCTTTCCTTACCAG
TCCTTGCCGGAGCAATTACAATACTTTTAACTGATCGAAATTTTAATACCACTTTCTTTGACCCAA
>BM20 COI
GGGTACCTCTTTAAGTTTAATAATTCGAACAGAATTAGGTCAACCAGGATCTCTTCTCAATGATGATCAATTATATAATGTTATTGTCACAGCCCATGCATTT
GTAATAATCTTTTTCCTTGTTATGCCCGTTATAATTGGAGGATTCGGCAACTGATTAGTTCCCTTAATATTAGGAGCCCCAGACATAGCATTTCCACGAATAA
ATAATATAAGTTTTTGACTTCTACCACCCTCTCTTACTTTATTACTATCCTCAGCTGCAGTAGAAAGAGGTGTAGGAACTGGATGAACCGTATACCCTCCTCT
TTCAAGAAATTTAGCTCATATAGGTCCATCCGTTGATTTAGCTATTTTTTCACTACATCTAGCTGGTATCTCATCTATTCTTGGAGCTATTAATTTTATTACA
ACTATCATTAATATACGATGAGAAGGAATACTAATAGAACGACTCCCATTATTTGTATGATCAGTATTTATTACTGCAATTTTATTACTGCTTTCCTTACCAG
TCCTTGCCGGAGCAATTACAATACTTTTAACTGATCGAAATTTTAATACCACTTTCTTTGACCCAA
>BM19 COI
GGGTACCTCTTTAAGTTTAATAATTCGAACAGAATTAGGTCAACCAGGATCTCTTCTCAATGATGATCAATTATATAATGTTATTGTCACAGCCCATGCATTT
GTAATAATCTTTTTCCTTGTTATGCCCGTTATAATTGGAGGATTCGGCAACTGATTAGTTCCCTTAATATTAGGAGCCCCAGACATAGCATTTCCACGAATAA
ATAATATAAGTTTTTGACTTCTACCACCCTCTCTTACTTTATTACTATCCTCAGCTGCAGTAGAAAGAGGTGTAGGAACTGGATGAACCGTATACCCTCCTCT
TTCAAGAAATTTAGCTCATATAGGTCCATCCGTTGATTTAGCTATTTTTTCACTACATCTAGCTGGTATCTCATCTATTCTTGGAGCTATTAATTTTATTACA
ACTATCATTAATATACGATGAGAAGGAATACTAATAGAACGACTCCCATTATTTGTATGATCAGTATTTATTACTGCAATTTTATTACTGCTTTCCTTACCAG
TCCTTGCCGGAGCAATTACAATACTTTTAACTGATCGAAATTTTAATACCACTTTCTTTGACCCAA
>BM18 COI
GGGTACCTCTTTAAGTTTAATAATTCGAACAGAATTAGGTCAACCAGGATCTCTTCTCAATGATGATCAATTATATAATGTTATTGTCACAGCCCATGCATTT
GTAATAATCTTTTTCCTTGTTATGCCCGTTATAATTGGAGGATTCGGCAACTGATTAGTTCCCTTAATATTAGGAGCCCCAGACATAGCATTTCCACGAATAA
ATAATATAAGTTTTTGACTTCTACCACCCTCTCTTACTTTATTACTATCCTCAGCTGCAGTAGAAAGAGGTGTAGGAACTGGATGAACCGTATACCCTCCTCT
TTCAAGAAATTTAGCTCATATAGGTCCATCCGTTGATTTAGCTATTTTTTCACTACATCTAGCTGGTATCTCATCTATTCTTGGAGCTATTAATTTTATTACA
ACTATCATTAATATACGATGAGAAGGAATACTAATAGAACGACTCCCATTATTTGTATGATCAGTATTTATTACTGCAATTTTATTACTGCTTTCCTTACCAG
TCCTTGCCGGAGCAATTACAATACTTTTAACTGATCGAAATTTTAATACCACTTTCTTTGACCCAA
>BM17 COI
GGGTACCTCTTTAAGTTTAATAATTCGAACAGAATTAGGTCAACCAGGATCTCTTCTCAATGATGATCAATTATATAATGTTATTGTCACAGCCCATGCATTT
GTAATAATCTTTTTCCTTGTTATGCCCGTTATAATTGGAGGATTCGGCAACTGATTAGTTCCCTTAATATTAGGAGCCCCAGACATAGCATTTCCACGAATAA
ATAATATAAGTTTTTGACTTCTACCACCCTCTCTTACTTTATTACTATCCTCAGCTGCAGTAGAAAGAGGTGTAGGAACTGGATGAACCGTATACCCTCCTCT
TTCAAGAAATTTAGCTCATATAGGTCCATCCGTTGATTTAGCTATTTTTTCACTACATCTAGCTGGTATCTCATCTATTCTTGGAGCTATTAATTTTATTACA
ACTATCATTAATATACGATGAGAAGGAATACTAATAGAACGACTCCCATTATTTGTATGATCAGTATTTATTACTGCAATTTTATTACTGCTTTCCTTACCAG
TCCTTGCCGGAGCAATTACAATACTTTTAACTGATCGAAATTTTAATACCACTTTCTTTGACCCAA
>BM16 COI
GGGTACCTCTTTAAGTTTAATAATTCGAACAGAATTAGGTCAACCAGGATCTCTTCTCAATGATGATCAATTATATAATGTTATTGTCACAGCCCATGCATTT
GTAATAATCTTTTTCCTTGTTATGCCCGTTATAATTGGAGGATTCGGCAACTGATTAGTTCCCTTAATATTAGGAGCCCCAGACATAGCATTTCCACGAATAA
ATAATATAAGTTTTTGACTTCTACCACCCTCTCTTACTTTATTACTATCCTCAGCTGCAGTAGAAAGAGGTGTAGGAACTGGATGAACCGTATACCCTCCTCT
TTCAAGAAATTTAGCTCATATAGGTCCATCCGTTGATTTAGCTATTTTTTCACTACATCTAGCTGGTATCTCATCTATTCTTGGAGCTATTAATTTTATTACA
ACTATCATTAATATACGATGAGAAGGAATACTAATAGAACGACTCCCATTATTTGTATGATCAGTATTTATTACTGCAATTTTATTACTGCTTTCCTTACCAG
TCCTTGCCGGAGCAATTACAATACTTTTAACTGATCGAAATTTTAATACCACTTTCTTTGACCCAA
>BLA290 COI
AGGTACTTCTTTAAGTTTAATAATTCGAACAGAATTAGGTCAACCAGGATCTCTTCTCAATGATGATCAATTATATAATGTTATTGTTACAGCCCATGCATTT
GTAATAATCTTTTTTCTTGTTATACCTGTCATAATTGGAGGATTTGGCAACTGATTAGTTCCCTTAATATTGGGAGCCCCAGATATAGCATTCCCACGAATAA
ATAACATAAGTTTTTGACTTTTACCACCCTCTCTCACTTTATTATTATCCTCAGCCGCAGTAGAAAGAGGTGTAGGAACTGGATGAACCGTATATCCCCCTCT
CTCAAGAAACTTAGCTCATATAGGTCCATCCGTTGATTTAGCTATTTTTTCACTCCACTTAGCCGGTATCTCATCTATCCTTGGAGCTATTAATTTTATTACA
ACTATTATTAATATACGATGAGAAGGAATACTAATAGAACGACTCCCATTATTTGTATGATCAGTATTTATTACTGCAATTTTATTATTACTTTCTCTACCAG
TTCTTGCTGGAGCAATTACAATACTTTTAACTGATCGAAATTTTAATACCACTTTTTTTGATCCGA
>BLA289 COI
AGGTACTTCTTTAAGTTTAATAATTCGAACAGAATTAGGTCAACCAGGATCTCTTCTCAATGATGATCAATTATATAATGTTATTGTTACAGCCCATGCATTT
GTAATAATCTTTTTTCTTGTTATACCTGTCATAATTGGAGGATTTGGCAACTGATTAGTTCCCTTAATATTGGGAGCCCCAGATATAGCATTCCCACGAATAA
ATAACATAAGTTTTTGACTTTTACCACCCTCTCTCACTTTATTATTATCCTCAGCCGCAGTAGAAAGAGGTGTAGGAACTGGATGAACCGTATATCCCCCTCT
CTCAAGAAACTTAGCTCATATAGGTCCATCCGTTGATTTAGCTATTTTTTCACTCCACTTAGCCGGTATCTCATCTATCCTTGGAGCTATTAATTTTATTACA
ACTATTATTAATATACGATGAGAAGGAATACTAATAGAACGACTCCCATTATTTGTATGATCAGTATTTATTACTGCAATTTTATTATTACTTTCTCTACCAG
TTCTTGCTGGAGCAATTACAATACTTTTAACTGATCGAAATTTTAATACCACTTTTTTTGATCCGA

>BLA288 COI
AGGTACTTCTTTAAGTTTAATAATTCGAACAGAATTAGGTCAACCAGGATCTCTTCTCAATGATGATCAATTATATAATGTTATTGTTACAGCCCATGCATTT
GTAATAATCTTTTTTCTTGTTATACCTGTCATAATTGGAGGATTTGGCAACTGATTAGTTCCCTTAATATTGGGAGCCCCAGATATAGCATTCCCACGAATAA
ATAACATAAGTTTTTGACTTTTACCACCCTCTCTCACTTTATTATTATCCTCAGCCGCAGTAGAAAGAGGTGTAGGAACTGGATGAACCGTATATCCCCCTCT
CTCAAGAAACTTAGCTCATATAGGTCCATCCGTTGATTTAGCTATTTTTTCACTCCACTTAGCCGGTATCTCATCTATCCTTGGAGCTATTAATTTTATTACA
ACTATTATTAATATACGATGAGAAGGAATACTAATAGAACGACTCCCATTATTTGTATGATCAGTATTTATTACTGCAATTTTATTATTACTTTCTCTACCAG
TTCTTGCTGGAGCAATTACAATACTTTTAACTGATCGAAATTTTAATACCACTTTTTTTGATCCGA
>BLA287 COI
AGGTACTTCTTTAAGTTTAATAATTCGAACAGAATTAGGTCAACCAGGATCTCTTCTCAATGATGATCAATTATATAATGTTATTGTTACAGCCCATGCATTT
GTAATAATCTTTTTTCTTGTTATACCTGTCATAATTGGAGGATTTGGCAACTGATTAGTTCCCTTAATATTGGGAGCCCCAGATATAGCATTCCCACGAATAA
ATAACATAAGTTTTTGACTTTTACCACCCTCTCTCACTTTATTATTATCCTCAGCCGCAGTAGAAAGAGGTGTAGGAACTGGATGAACCGTATATCCCCCTCT
CTCAAGAAACTTAGCTCATATAGGTCCATCCGTTGATTTAGCTATTTTTTCACTCCACTTAGCCGGTATCTCATCTATCCTTGGAGCTATTAATTTTATTACA
ACTATTATTAATATACGATGAGAAGGAATACTAATAGAACGACTCCCATTATTTGTATGATCAGTATTTATTACTGCAATTTTATTATTACTTTCTCTACCAG
TTCTTGCTGGAGCAATTACAATACTTTTAACTGATCGAAATTTTAATACCACTTTTTTTGATCCGA
>BLA286 COI
AGGTACTTCTTTAAGTTTAATAATTCGAACAGAATTAGGTCAACCAGGATCTCTTCTCAATGATGATCAATTATATAATGTTATTGTTACAGCCCATGCATTT
GTAATAATCTTTTTTCTTGTTATACCTGTCATAATTGGAGGATTTGGCAACTGATTAGTTCCCTTAATATTGGGAGCCCCAGATATAGCATTCCCACGAATAA
ATAACATAAGTTTTTGACTTTTACCACCCTCTCTCACTTTATTATTATCCTCAGCCGCAGTAGAAAGAGGTGTAGGAACTGGATGAACCGTATATCCCCCTCT
CTCAAGAAACTTAGCTCATATAGGTCCATCCGTTGATTTAGCTATTTTTTCACTCCACTTAGCCGGTATCTCATCTATCCTTGGAGCTATTAATTTTATTACA
ACTATTATTAATATACGATGAGAAGGAATACTAATAGAACGACTCCCATTATTTGTATGATCAGTATTTATTACTGCAATTTTATTATTACTTTCTCTACCAG
TTCTTGCTGGAGCAATTACAATACTTTTAACTGATCGAAATTTTAATACCACTTTTTTTGATCCGA
>BLA285 COI
AGGTACTTCTTTAAGTTTAATAATTCGAACAGAATTAGGTCAACCAGGATCTCTTCTCAATGATGATCAATTATATAATGTTATTGTTACAGCCCATGCATTT
GTAATAATCTTTTTTCTTGTTATACCTGTCATAATTGGAGGATTTGGCAACTGATTAGTTCCCTTAATATTGGGAGCCCCAGATATAGCATTCCCACGAATAA
ATAACATAAGTTTTTGACTTTTACCACCCTCTCTCACTTTATTATTATCCTCAGCCGCAGTAGAAAGAGGTGTAGGAACTGGATGAACCGTATATCCCCCTCT
CTCAAGAAACTTAGCTCATATAGGTCCATCCGTTGATTTAGCTATTTTTTCACTCCACTTAGCCGGTATCTCATCTATCCTTGGAGCTATTAATTTTATTACA
ACTATTATTAATATACGATGAGAAGGAATACTAATAGAACGACTCCCATTATTTGTATGATCAGTATTTATTACTGCAATTTTATTATTACTTTCTCTACCAG
TTCTTGCTGGAGCAATTACAATACTTTTAACTGATCGAAATTTTAATACCACTTTTTTTGATCCGA
>BLA284 COI
AGGTACTTCTTTAAGTTTAATAATTCGAACAGAATTAGGTCAACCAGGATCTCTTCTCAATGATGATCAATTATATAATGTTATTGTTACAGCCCATGCATTT
GTAATAATCTTTTTTCTTGTTATACCTGTCATAATTGGAGGATTTGGCAACTGATTAGTTCCCTTAATATTGGGAGCCCCAGATATAGCATTCCCACGAATAA
ATAACATAAGTTTTTGACTTTTACCACCCTCTCTCACTTTATTATTATCCTCAGCCGCAGTAGAAAGAGGTGTAGGAACTGGATGAACCGTATATCCCCCTCT
CTCAAGAAACTTAGCTCATATAGGTCCATCCGTTGATTTAGCTATTTTTTCACTCCACTTAGCCGGTATCTCATCTATCCTTGGAGCTATTAATTTTATTACA
ACTATTATTAATATACGATGAGAAGGAATACTAATAGAACGACTCCCATTATTCGTATGATCAGTATTTATTACTGCAATTTTATTATTACTTTCTCTACCAG
TTCTTGCTGGAGCAATTACAATACTTTTAACTGATCGAAATTTTAATACCACTTTTTTTGATCCGA
>BLA283 COI
AGGTACTTCTTTAAGTTTAATAATTCGAACAGAATTAGGTCAACCAGGATCTCTTCTCAATGATGATCAATTATATAATGTTATTGTTACAGCCCATGCATTT
GTAATAATCTTTTTTCTTGTTATACCTGTCATAATTGGAGGATTTGGCAACTGATTAGTTCCCTTAATATTGGGAGCCCCAGATATAGCATTCCCACGAATAA
ATAACATAAGTTTTTGACTTTTACCACCCTCTCTCACTTTATTATTATCCTCAGCCGCAGTAGAAAGAGGTGTAGGAACTGGATGAACCGTATATCCCCCTCT
CTCAAGAAACTTAGCTCATATAGGTCCATCCGTTGATTTAGCTATTTTTTCACTCCACTTAGCCGGTATCTCATCTATCCTTGGAGCTATTAATTTTATTACA
ACTATTATTAATATACGATGAGAAGGAATACTAATAGAACGACTCCCATTATTTGTATGATCAGTATTTATTACTGCAATTTTATTATTACTTTCTCTACCAG
TTCTTGCTGGAGCAATTACAATACTTTTAACTGATCGAAATTTTAATACCACTTTTTTTGATCCGA
>BKI395 COI
AGGTACCTCTCTAAGTTTAATAATTCGAACAGAATTAGGTCAACCAGGATCTCTCCTAAATGATGATCAACTATATAACGTAATTGTTACAGCACATGCATTT
GTAATAATTTTTTTTCTTGTTATACCTGTTATAATTGGAGGATTTGGTAATTGATTAGTTCCCTTAATACTAGGAGCTCCAGACATAGCATTCCCACGAATAA
ACAATATAAGTTTTTGACTTTTACCACCTTCTCTTACCCTATTATTATCTTCAGCTGCAGTAGAAAGAGGTGCAGGTACTGGATGGACTGTATATCCCCCTCT
TTCAAGAAATTTAGCTCATATAGGCCCTTCCGTTGATCTAGCTATTTTTTCTCTTCATTTAGCTGGTATTTCATCTATTCTTGGAGCCATTAACTTCATTACA
ACTATTATCAATATACGATGAGAAGGAATATTAATAGAACGACTTCCATTATTTGTATGATCAGTATTTATTACCGCAATTTTATTACTTCTTTCCTTACCAG
TCCTTGCTGGAGCAATTACAATACTCTTAACAGATCGAAATTTTAATACCACTTTTTTTGACCCAA
>BKI394 COI
AGGTACCTCTCTAAGTTTAATAATTCGAACAGAATTAGGTCAACCAGGATCTCTCCTAAATGATGATCAACTATATAACGTAATTGTTACAGCACATGCATTT
GTAATAATTTTTTTTCTTGTTATACCTGTTATAATTGGAGGATTTGGTAATTGATTAGTTCCCTTAATACTAGGAGCTCCAGACATAGCATTCCCACGAATAA
ACAATATAAGTTTTTGACTTTTACCACCTTCTCTTACCCTATTATTATCTTCAGCTGCAGTAGAAAGAGGTGCAGGTACTGGATGGACTGTATATCCCCCTCT
TTCAAGAAATTTAGCTCATATAGGCCCTTCCGTTGACCTAGCTATTTTTTCTCTTCATTTAGCTGGTATTTCATCTATTCTTGGAGCCATTAACTTCATTACA
ACTATTATCAATATACGATGAGAAGGAATATTAATAGAACGACTTCCATTATTTGTATGATCAGTATTTATTACCGCAATTTTATTACTTCTTTCCTTACCAG
TCCTTGCTGGAGCAATTACAATACTCTTAACAGATCGAAATTTTAATACCACTTTTTTTGACCCAA
>BKI393 COI
AGGTACCTCTCTAAGTTTAATAATTCGAACAGAATTAGGTCAACCAGGATCTCTCCTAAATGATGATCAACTATATAACGTAATTGTTACAGCACATGCATTT
GTAATAATTTTTTTTCTTGTTATACCTGTTATAATTGGAGGATTTGGTAATTGATTAGTTCCCTTAATACTAGGAGCTCCAGACATAGCATTCCCACGAATAA
ACAATATAAGTTTTTGACTTTTACCACCTTCTCTTACCCTATTATTATCTTCAGCTGCAGTAGAAAGAGGTGCAGGTACTGGATGGACTGTATATCCCCCTCT
TTCAAGAAATTTAGCTCATATAGGCCCTTCCGTTGACCTAGCTATTTTTTCTCTTCATTTAGCTGGTATTTCATCTATTCTTGGAGCCATTAACTTCATTACA
ACTATTATCAATATACGATGAGAAGGAATATTAATAGAACGACTTCCATTATTTGTATGATCAGTATTTATTACCGCAATTTTATTACTTCTTTCCTTACCAG
TCCTTGCTGGAGCAATTACAATACTCTTAACAGATCGAAATTTTAATACCACTTTTTTTGACCCAA
>BKI392 COI
AGGTACCTCTCTAAGTTTAATAATTCGAACAGAATTAGGTCAACCAGGATCTCTCCTAAATGATGATCAACTATATAACGTAATTGTTACAGCACATGCATTT
GTAATAATTTTTTTTCTTGTTATACCTGTTATAATTGGAGGATTTGGTAATTGATTAGTTCCCTTAATACTAGGAGCTCCAGACATAGCATTCCCACGAATAA
ACAATATAAGTTTTTGACTTTTACCACCTTCTCTTACCCTATTATTATCTTCAGCTGCAGTAGAAAGAGGTGCAGGTACTGGATGGACTGTATATCCCCCTCT
TTCAAGAAATTTAGCTCATATAGGCCCTTCCGTTGATCTAGCTATTTTTTCTCTTCATTTAGCTGGTATTTCATCTATTCTTGGAGCCATTAACTTCATTACA
ACTATTATCAATATACGATGAGAAGGAATATTAATAGAACGACTTCCATTATTTGTATGATCAGTATTTATTACCGCAATTTTATTACTTCTTTCCTTACCAG
TCCTTGCTGGAGCAATTACAATACTCTTAACAGATCGAAATTTTAATACCACTTTTTTTGACCCAA
>BKI391 COI
AGGTACCTCTCTAAGTTTAATAATTCGAACAGAATTAGGTCAACCAGGATCTCTCCTAAATGATGATCAACTATATAACGTAATTGTTACAGCACATGCATTT
GTAATAATTTTTTTTCTTGTTATACCTGTTATAATTGGAGGATTTGGTAATTGATTAGTTCCCTTAATACTAGGAGCTCCAGACATAGCATTCCCACGAATAA
ACAATATAAGTTTTTGACTTTTACCACCTTCTCTTACCCTATTATTATCTTCAGCTGCAGTAGAAAGAGGTGCAGGTACTGGATGGACTGTATATCCCCCTCT
TTCAAGAAATTTAGCTCATATAGGCCCTTCCGTTGATCTAGCTATTTTTTCTCTTCATTTAGCTGGTATTTCATCTATTCTTGGAGCCATTAACTTCATTACA
ACTATTATCAATATACGATGAGAAGGAATATTAATAGAACGACTTCCATTATTTGTATGATCAGTATTTATTACCGCAATTTTATTACTTCTTTCCTTACCAG
TCCTTGCTGGAGCAATTACAATACTCTTAACAGATCGAAATTTTAATACCACTTTTTTTGACCCAA
>BKI390 COI
AGGTACCTCTCTAAGTTTAATAATTCGAACAGAATTAGGTCAACCAGGATCTCTCCTAAATGATGATCAACTATATAACGTAATTGTTACAGCACATGCATTT

GTAATAATTTTTTTTCTTGTTATACCTGTTATAATTGGAGGATTTGGTAATTGATTAGTTCCCTTAATACTAGGAGCTCCAGACATAGCATTCCCACGAATAA
ACAATATAAGTTTTTGACTTTTACCACCTTCTCTTACCCTATTATTATCTTCAGCTGCAGTAGAAAGAGGTGCAGGTACTGGATGGACTGTATATCCCCCTCT
TTCAAGAAATTTAGCTCATATAGGCCCTTCCGTTGACCTAGCTATTTTTTCTCTTCATTTAGCTGGTATTTCATCTATTCTTGGAGCCATTAACTTCATTACA
ACTATTATCAATATACGATGAGAAGGAATATTAATAGAACGACTTCCATTATTTGTATGATCAGTATTTATTACCGCAATTTTATTACTTCTTTCCTTACCAG
TCCTTGCTGGAGCAATTACAATACTCTTAACAGATCGAAATTTTAATACCACTTTTTTTGACCCAA
>BKI389 COI
AGGTACCTCTCTAAGTTTAATAATTCGAACAGAATTAGGTCAACCAGGATCTCTCCTAAATGATGATCAACTATATAACGTAATTGTTACAGCACATGCATTT
GTAATAATTTTTTTTCTTGTTATACCTGTTATAATTGGAGGATTTGGTAATTGATTAGTTCCCTTAATACTAGGAGCTCCAGACATAGCATTCCCACGAATAA
ACAATATAAGTTTTTGACTTTTACCACCTTCTCTTACCCTATTATTATCTTCAGCTGCAGTAGAAAGAGGTGCAGGTACTGGATGGACTGTATATCCCCCTCT
TTCAAGAAATTTAGCTCATATAGGCCCTTCCGTTGACCTAGCTATTTTTTCTCTTCATTTAGCTGGTATTTCATCTATTCTTGGAGCCATTAACTTCATTACA
ACTATTATCAATATACGATGAGAAGGAATATTAATAGAACGACTTCCATTATTTGTATGATCAGTATTTATTACCGCAATTTTATTACTTCTTTCCTTACCAG
TCCTTGCTGGAGCAATTACAATACTCTTAACAGATCGAAATTTTAATACCACTTTTTTTGACCCAA
>BKI388 COI
AGGTACCTCTCTAAGTTTAATAATTCGAACAGAATTAGGTCAACCAGGATCTCTCCTAAATGATGATCAACTATATAACGTAATTGTTACAGCACATGCATTT
GTAATAATTTTTTTTCTTGTTATACCTGTTATAATTGGAGGATTTGGTAATTGATTAGTTCCCTTAATACTAGGAGCTCCAGACATAGCATTCCCACGAATAA
ACAATATAAGTTTTTGACTTTTACCACCTTCTCTTACCCTATTATTATCTTCAGCTGCAGTAGAAAGAGGTGCAGGTACTGGATGGACTGTATATCCCCCTCT
TTCAAGAAATTTAGCTCATATAGGCCCTTCCGTTGATCTAGCTATTTTTTCTCTTCATTTAGCTGGTATTTCATCTATTCTTGGAGCCATTAACTTCATTACA
ACTATTATCAATATACGATGAGAAGGAATATTAATAGAACGACTTCCATTATTTGTATGATCAGTATTTATTACCGCAATTTTATTACTTCTTTCCTTACCAG
TCCTTGCTGGAGCAATTACAATACTCTTAACAGATCGAAATTTTAATACCACTTTTTTTGACCCAA
