# Supplementary material for: Life histories predict genetic diversity and population structure within three species of octopus targeted by small-scale fisheries in Northwest Mexico
Source: PeerJ. 2018 Feb 15;6:e4295. doi: 10.7717/peerj.4295 (PMC5816968; doi:10.7717/peerj.4295)
Supplement: Supplemental Information 2 [file peerj-06-4295-s009.pdf]

```
>STR184 COI
GTSLSLMIRTELGQPGSLLNDDQLYNVIVTAHAFVMIFFLVMPVMIGGFGNWLVPLMLGAPDMAFPRMNNMSFWLLPPSLTLLLSSAAVESGAGTGWTVYPPL
SSNLAHMGPSVDLAIFSLHLAGISSILGAINFITTIINMRWEGMLMERLPLFVWSVFITAILLLLSLPVLAGAITMLLTDRNFNTTFFDP
>STR183 COI
GTSLSLMIRTELGQPGSLLNDDQLYNVIVTAHAFVMIFFLVMPVMIGGFGNWLVPLMLGAPDMAFPRMNNMSFWLLPPSLTLLLSSAAVESGAGTGWTVYPPL
SSNLAHMGPSVDLAIFSLHLAGISSILGAINFITTIINMRWEGMLMERLPLFVWSVFITAILLLLSLPVLAGAITMLLTDRNFNTTFFDP
>STR182 COI
GTSLSLMIRTELGQPGSLLNDDQLYNVIVTAHAFVMIFFLVMPVMIGGFGNWLVPLMLGAPDMAFPRMNNMSFWLLPPSLTLLLSSAAVESGAGTGWTVYPPL
SSNLAHMGPSVDLAIFSLHLAGISSILGAINFITTIINMRWEGMLMERLPLFVWSVFITAILLLLSLPVLAGAITMLLTDRNFNTTFFDP
>STR181 COI
GTSLSLMIRTELGQPGSLLNDDQLYNVIVTAHAFVMIFFLVMPVMIGGFGNWLVPLMLGAPDMAFPRMNNMSFWLLPPSLTLLLSSAAVESGAGTGWTVYPPL
SSNLAHMGPSVDLAIFSLHLAGISSILGAINFITTIINMRWEGMLMERLPLFVWSVFITAILLLLSLPVLAGAITMLLTDRNFNTTFFDP
>STR180 COI
GTSLSLMIRTELGQPGSLLNDDQLYNVIVTAHAFVMIFFLVMPVMIGGFGNWLVPLMLGAPDMAFPRMNNMSFWLLPPSLTLLLSSAAVESGAGTGWTVYPPL
SSNLAHMGPSVDLAIFSLHLAGISSILGAINFITTIINMRWEGMLMERLPLFVWSVFITAILLLLSLPVLAGAITMLLTDRNFNTTFFDP
>STR179 COI
GTSLSLMIRTELGQPGSLLNDDQLYNVIVTAHAFVMIFFLVMPVMIGGFGNWLVPLMLGAPDMAFPRMNNMSFWLLPPSLTLLLSSAAVESGAGTGWTVYPPL
SSNLAHMGPSVDLAIFSLHLAGISSILGAINFITTIINMRWEGMLMERLPLFVWSVFITAILLLLSLPVLAGAITMLLTDRNFNTTFFDP
>STR178 COI
GTSLSLMIRTELGQPGSLLNDDQLYNVIVTAHAFVMIFFLVMPVMIGGFGNWLVPLMLGAPDMAFPRMNNMSFWLLPPSLTLLLSSAAVESGAGTGWTVYPPL
SSNLAHMGPSVDLAIFSLHLAGISSILGAINFITTIINMRWEGMLMERLPLFVWSVFITAILLLLSLPVLAGAITMLLTDRNFNTTFFDP
>STR177 COI
GTSLSLMIRTELGQPGSLLNDDQLYNVIVTAHAFVMIFFLVMPVMIGGFGNWLVPLMLGAPDMAFPRMNNMSFWLLPPSLTLLLSSAAVESGAGTGWTVYPPL
SSNLAHMGPSVDLAIFSLHLAGISSILGAINFITTIINMRWEGMLMERLPLFVWSVFITAILLLLSLPVLAGAITMLLTDRNFNTTFFDP
>SQ96 COI
GTSLSLMIRTELGQPGSLLNDDQLYNVIVTAHAFVMIFFLVMPVMIGGFGNWLVPLMLGAPDMAFPRMNNMSFWLLPPSLTLLLSSAAVESGVGTGWTVYPPL
SSNLAHMGPSVDLAIFSLHLAGISSILGAINFITTIINMRWEGMLMERLPLFVWSVFITAILLLLSLPVLAGAITMLLTDRNFNTTFFDP
>SQ95 COI
GTSLSLMIRTELGQPGSLLNDDQLYNVIVTAHAFVMIFFLVMPVMIGGFGNWLVPLMLGAPDMAFPRMNNMSFWLLPPSLTLLLSSAAVESGVGTGWTVYPPL
SSNLAHMGPSVDLAIFSLHLAGISSILGAINFITTIINMRWEGMLMERLPLFVWSVFITAILLLLSLPVLAGAITMLLTDRNFNTTFFDP
>SQ94 COI
GTSLSLMIRTELGQPGSLLNDDQLYNVIVTAHAFVMIFFLVMPVMIGGFGNWLVPLMLGAPDMAFPRMNNMSFWLLPPSLTLLLSSAAVESGVGTGWTVYPPL
SSNLAHMGPSVDLAIFSLHLAGISSILGAINFITTIINMRWEGMLMERLPLFVWSVFITAILLLLSLPVLAGAITMLLTDRNFNTTFFDP
>SQ93 COI
GTSLSLMIRTELGQPGSLLNDDQLYNVIVTAHAFVMIFFLVMPVMIGGFGNWLVPLMLGAPDMAFPRMNNMSFWLLPPSLTLLLSSAAVESGVGTGWTVYPPL
SSNLAHMGPSVDLAIFSLHLAGISSILGAINFITTIINMRWEGMLMERLPLFVWSVFITAILLLLSLPVLAGAITMLLTDRNFNTTFFDP
>SQ92 COI
GTSLSLMIRTELGQPGSLLNDDQLYNVIVTAHAFVMIFFLVMPVMIGGFGNWLVPLMLGAPDMAFPRMNNMSFWLLPPSLTLLLSSAAVESGVGTGWTVYPPL
SSNLAHMGPSVDLAIFSLHLAGISSILGAINFITTIINMRWEGMLMERLPLFVWSVFITAILLLLSLPVLAGAITMLLTDRNFNTTFFDP
>SQ91 COI
GTSLSLMIRTELGQPGSLLNDDQLYNVIVTAHAFVMIFFLVMPVMIGGFGNWLVPLMLGAPDMAFPRMNNMSFWLLPPSLTLLLSSAAVESGVGTGWTVYPPL
SSNLAHMGPSVDLAIFSLHLAGISSILGAINFITTIINMRWEGMLMERLPLFVWSVFITAILLLLSLPVLAGAITMLLTDRNFNTTFFDP
>SQ90 COI
GTSLSLMIRTELGQPGSLLNDDQLYNVIVTAHAFVMIFFLVMPVMIGGFGNWLVPLMLGAPDMAFPRMNNMSFWLLPPSLTLLLSSAAVESGVGTGWTVYPPL
SSNLAHMGPSVDLAIFSLHLAGISSILGAINFITTIINMRWEGMLMERLPLFVWSVFITAILLLLSLPVLAGAITMLLTDRNFNTTFFDP
>SQ89 COI
GTSLSLMIRTELGQPGSLLNDDQLYNVIVTAHAFVMIFFLVMPVMIGGFGNWLVPLMLGAPDMAFPRMNNMSFWLLPPSLTLLLSSAAVESGVGTGWTVYPPL
SSNLAHMGPSVDLAIFSLHLAGISSILGAINFITTIINMRWEGMLMERLPLFVWSVFITAILLLLSLPVLAGAITMLLTDRNFNTTFFDP
>PRE268 COI
GTSLSLMIRTELGQPGSLLNDDQLYNVIVTAHAFVMIFFLVMPVMIGGFGNWLVPLMLGAPDMAFPRMNNMSFWLLPPSLTLLLSSAAVESGVGTGWTVYPPL
SSNLAHMGPSVDLAIFSLHLAGISSILGAINFITTIINMRWEGMLMERLPLFVWSVFITAILLLLSLPVLAGAITMLLTDRNFNTTFFDP
>PPE304 COI
GTSLSLMIRTELGQPGSLLNDDQLYNVIVTAHAFVMIFFLVMPVMIGGFGNWLVPLMLGAPDMAFPRMNNMSFWLLPPSLTLLLSSAAVESGVGTGWTVYPPL
SSNLAHMGPSVDLAIFSLHLAGISSILGAINFITTIINMRWEGMLMERLPLFVWSVFITAILLLLSLPVLAGAITMLLTDRNFNTTFFDP
>PPE303 COI
GTSLSLMIRTELGQPGSLLNDDQLYNVIVTAHAFVMIFFLVMPVMIGGFGNWLVPLMLGAPDMAFPRMNNMSFWLLPPSLTLLLSSAAVESGVGTGWTVYPPL
SSNLAHMGPSVDLAIFSLHLAGISSILGAINFITTIINMRWEGMLMERLPLFVWSVFITAILLLLSLPVLAGAITMLLTDRNFNTTFFDP
>PPE302 COI
GTSLSLMIRTELGQPGSLLNDDQLYNVIVTAHAFVMIFFLVMPVMIGGFGNWLVPLMLGAPDMAFPRMNNMSFWLLPPSLTLLLSSAAVESGVGTGWTVYPPL
SSNLAHMGPSVDLAIFSLHLAGISSILGAINFITTIINMRWEGMLMERLPLFVWSVFITAILLLLSLPVLAGAITMLLTDRNFNTTFFDP
>PPE301 COI
GTSLSLMIRTELGQPGSLLNDDQLYNVIVTAHAFVMIFFLVMPVMIGGFGNWLVPLMLGAPDMAFPRMNNMSFWLLPPSLTLLLSSAAVESGVGTGWTVYPPL
SSNLAHMGPSVDLAIFSLHLAGISSILGAINFITTIINMRWEGMLMERLPLFVWSVFITAILLLLSLPVLAGAITMLLTDRNFNTTFFDP
>PPE300 COI
GTSLSLMIRTELGQPGSLLNDDQLYNVIVTAHAFVMIFFLVMPVMIGGFGNWLVPLMLGAPDMAFPRMNNMSFWLLPPSLTLLLSSAAVESGVGTGWTVYPPL
SSNLAHMGPSVDLAIFSLHLAGISSILGAINFITTIINMRWEGMLMERLPLFVWSVFITAILLLLSLPVLAGAITMLLTDRNFNTTFFDP
>PPE299 COI
GTSLSLMIRTELGQPGSLLNDDQLYNVIVTAHAFVMIFFLVMPVMIGGFGNWLVPLMLGAPDMAFPRMNNMSFWLLPPSLTLLLSSAAVESGVGTGWTVYPPL
SSNLAHMGPSVDLAIFSLHLAGISSILGAINFITTIINMRWEGMLMERLPLFVWSVFITAILLLLSLPVLAGAITMLLTDRNFNTTFFDP
>PPE298 COI
GTSLSLMIRTELGQPGSLLNDDQLYNVIVTAHAFVMIFFLVMPVMIGGFGNWLVPLMLGAPDMAFPRMNNMSFWLLPPSLTLLLSSAAVESGVGTGWTVYPPL
SSNLAHMGPSVDLAIFSLHLAGISSILGAINFITTIINMRWEGMLMERLPLFVWSVFITAILLLLSLPVLAGAITMLLTDRNFNTTFFDP
>PPE297 COI
GTSLSLMIRTELGQPGSLLNDDQLYNVIVTAHAFVMIFFLVMPVMIGGFGNWLVPLMLGAPDMAFPRMNNMSFWLLPPSLTLLLSSAAVESGVGTGWTVYPPL
SSNLAHMGPSVDLAIFSLHLAGISSILGAINFITTIINMRWEGMLMERLPLFVWSVFITAILLLLSLPVLAGAITMLLTDRNFNTTFFDP
>PLO363 COI
GTSLSLMIRTELGQPGSLLNDDQLYNVIVTAHAFVMIFFLVMPVMIGGFGNWLVPLMLGAPDMAFPRMNNMSFWLLPPSLTLLLSSAAVESGVGTGWTVYPPL
SSNLAHMGPSVDLAIFSLHLAGISSILGAINFITTIINMRWEGMLMERLPLFVWSVFITAILLLLSLPVLAGAITMLLTDRNFNTTFFDP
>PLO362 COI
```

GTSLSLMIRTELGQPGSLLNDDQLYNVIVTAHAFVMIFFLVMPVMIGGFGNWLVPLMLGAPDMAFPRMNNMSFWLLPPSLTLLLSSAAVESGVGTGWTVYPPL
SSNLAHMGPSVDLAIFSLHLAGISSILGAINFITTIINMRWEGMLMERLPLFVWSVFITAILLLLSLPVLAGAITMLLTDRNFNTTFFDP
>PLO361 COI
GTSLSLMIRTELGQPGSLLNDDQLYNVIVTAHAFVMIFFLVMPVMIGGFGNWLVPLMLGAPDMAFPRMNNMSFWLLPPSLTLLLSSAAVESGVGTGWTVYPPL
SSNLAHMGPSVDLAIFSLHLAGISSILGAINFITTIINMRWEGMLMERLPLFVWSVFITAILLLLSLPVLAGAITMLLTDRNFNTTFFDP
>PLO360 COI
GTSLSLMIRTELGQPGSLLNDDQLYNVIVTAHAFVMIFFLVMPVMIGGFGNWLVPLMLGAPDMAFPRMNNMSFWLLPPSLTLLLSSAAVESGVGTGWTVYPPL
SSNLAHMGPSVDLAIFSLHLAGISSILGAINFITTIINMRWEGMLMERLPLFVWSVFITAILLLLSLPVLAGAITMLLTDRNFNTTFFDP
>PLO359 COI
GTSLSLMIRTELGQPGSLLNDDQLYNVIVTAHAFVMIFFLVMPVMIGGFGNWLVPLMLGAPDMAFPRMNNMSFWLLPPSLTLLLSSAAVESGVGTGWTVYPPL
SSNLAHMGPSVDLAIFSLHLAGISSILGAINFITTIINMRWEGMLMERLPLFVWSVFITAILLLLSLPVLAGAITMLLTDRNFNTTFFDP
>PLO358 COI
GTSLSLMIRTELGQPGSLLNDDQLYNVIVTAHAFVMIFFLVMPVMIGGFGNWLVPLMLGAPDMAFPRMNNMSFWLLPPSLTLLLSSAAVESGVGTGWTVYPPL
SSNLAHMGPSVDLAIFSLHLAGISSILGAINFITTIINMRWEGMLMERLPLFVWSVFITAILLLLSLPVLAGAITMLLTDRNFNTTFFDP
>PLO357 COI
GTSLSLMIRTELGQPGSLLNDDQLYNVIVTAHAFVMIFFLVMPVMIGGFGNWLVPLMLGAPDMAFPRMNNMSFWLLPPSLTLLLSSAAVESGVGTGWTVYPPL
SSNLAHMGPSVDLAIFSLHLAGISSILGAINFITTIINMRWEGMLMERLPLFVWSVFITAILLLLSLPVLAGAITMLLTDRNFNTTFFDP
>PLO356 COI
GTSLSLMIRTELGQPGSLLNDDQLYNVIVTAHAFVMIFFLVMPVMIGGFGNWLVPLMLGAPDMAFPRMNNMSFWLLPPSLTLLLSSAAVESGVGTGWTVYPPL
SSNLAHMGPSVDLAIFSLHLAGISSILGAINFITTIINMRWEGMLMERLPLFVWSVFITAILLLLSLPVLAGAITMLLTDRNFNTTFFDP
>PLI450 COI
GTSLSLMIRTELGQPGSLLNDDQLYNVIVTAHAFVMIFFLVMPVMIGGFGNWLVPLMLGAPDMAFPRMNNMSFWLLPPSLTLLLSSAAVESGAGTGWTVYPPL
SSNLAHMGPSVDLAIFSLHLAGISSILGAINFITTIINMRWEGMLMERLPLFVWSVFITAILLLLSLPVLAGAITMLLTDRNFNTTFFDP
>PLI449 COI
GTSLSLMIRTELGQPGSLLNDDQLYNVIVTAHAFVMIFFLVMPVMIGGFGNWLVPLMLGAPDMAFPRMNNMSFWLLPPSLTLLLSSAAVESGAGTGWTVYPPL
SSNLAHMGPSVDLAIFSLHLAGISSILGAINFITTIINMRWEGMLMERLPLFVWSVFITAILLLLSLPVLAGAITMLLTDRNFNTTFFDP
>PLI448 COI
GTSLSLMIRTELGQPGSLLNDDQLYNVIVTAHAFVMIFFLVMPVMIGGFGNWLVPLMLGAPDMAFPRMNNMSFWLLPPSLTLLLSSAAVESGAGTGWTVYPPL
SSNLAHMGPSVDLAIFSLHLAGISSILGAINFITTIINMRWEGMLMERLPLFVWSVFITAILLLLSLPVLAGAITMLLTDRNFNTTFFDP
>PLI447 COI
GTSLSLMIRTELGQPGSLLNDDQLYNVIVTAHAFVMIFFLVMPVMIGGFGNWLVPLMLGAPDMAFPRMNNMSFWLLPPSLTLLLSSAAVESGAGTGWTVYPPL
SSNLAHMGPSVDLAIFSLHLAGISSILGAINFITTIINMRWEGMLMERLPLFVWSVFITAILLLLSLPVLAGAITMLLTDRNFNTTFFDP
>PLI446 COI
GTSLSLMIRTELGQPGSLLNDDQLYNVIVTAHAFVMIFFLVMPVMIGGFGNWLVPLMLGAPDMAFPRMNNMSFWLLPPSLTLLLSSAAVESGAGTGWTVYPPL
SSNLAHMGPSVDLAIFSLHLAGISSILGAINFITTIINMRWEGMLMERLPLFVWSVFITAILLLLSLPVLAGAITMLLTDRNFNTTFFDP
>PLI444 COI
GTSLSLMIRTELGQPGSLLNDDQLYNVIVTAHAFVMIFFLVMPVMIGGFGNWLVPLMLGAPDMAFPRMNNMSFWLLPPSLTLLLSSAAVESGAGTGWTVYPPL
SSNLAHMGPSVDLAIFSLHLAGISSILGAINFITTIINMRWEGMLMERLPLFVWSVFITAILLLLSLPVLAGAITMLLTDRNFNTTFFDP
>PLI443 COI
GTSLSLMIRTELGQPGSLLNDDQLYNVIVTAHAFVMIFFLVMPVMIGGFGNWLVPLMLGAPDMAFPRMNNMSFWLLPPSLTLLLSSAAVESGAGTGWTVYPPL
SSNLAHMGPSVDLAIFSLHLAGISSILGAINFITTIINMRWEGMLMERLPLFVWSVFITAILLLLSLPVLAGAITMLLTDRNFNTTFFDP
>MAL126 COI
GTSLSLMIRTELGQPGSLLNDDQLYNVIVTAHAFVMIFFLVMPVMIGGFGNWLVPLMLGAPDMAFPRMNNMSFWLLPPSLTLLLSSAAVESGVGTGWTVYPPL
SSNLAHMGPSVDLAIFSLHLAGISSILGAINFITTIINMRWEGMLMERLPLFVWSVFITAILLLLSLPVLAGAITMLLTDRNFNTTFFDP
>MAL125 COI
GTSLSLMIRTELGQPGSLLNDDQLYNVIVTAHAFVMIFFLVMPVMIGGFGNWLVPLMLGAPDMAFPRMNNMSFWLLPPSLTLLLSSAAVESGVGTGWTVYPPL
SSNLAHMGPSVDLAIFSLHLAGISSILGAINFITTIINMRWEGMLMERLPLFVWSVFITAILLLLSLPVLAGAITMLLTDRNFNTTFFDP
>MAL124 COI
GTSLSLMIRTELGQPGSLLNDDQLYNVIVTAHAFVMIFFLVMPVMIGGFGNWLVPLMLGAPDMAFPRMNNMSFWLLPPSLTLLLSSAAVESGVGTGWTVYPPL
SSNLAHMGPSVDLAIFSLHLAGISSILGAINFITTIINMRWEGMLMERLPLFVWSVFITAILLLLSLPVLAGAITMLLTDRNFNTTFFDP
>MAL123 COI
GTSLSLMIRTELGQPGSLLNDDQLYNVIVTAHAFVMIFFLVMPVMIGGFGNWLVPLMLGAPDMAFPRMNNMSFWLLPPSLTLLLSSAAVESGVGTGWTVYPPL
SSNLAHMGPSVDLAIFSLHLAGISSILGAINFITTIINMRWEGMLMERLPLFVWSVFITAILLLLSLPVLAGAITMLLTDRNFNTTFFDP
>MAL121 COI
GTSLSLMIRTELGQPGSLLNDDQLYNVIVTAHAFVMIFFLVMPVMIGGFGNWLVPLMLGAPDMAFPRMNNMSFWLLPPSLTLLLSSAAVESGVGTGWTVYPPL
SSNLAHMGPSVDLAIFSLHLAGISSILGAINFITTIINMRWEGMLMERLPLFVWSVFITAILLLLSLPVLAGAITMLLTDRNFNTTFFDP
>MAL120 COI
GTSLSLMIRTELGQPGSLLNDDQLYNVIVTAHAFVMIFFLVMPVMIGGFGNWLVPLMLGAPDMAFPRMNNMSFWLLPPSLTLLLSSAAVESGVGTGWTVYPPL
SSNLAHMGPSVDLAIFSLHLAGISSILGAINFITTIINMRWEGMLMERLPLFVWSVFITAILLLLSLPVLAGAITMLLTDRNFNTTFFDP
>MAL119 COI
GTSLSLMIRTELGQPGSLLNDDQLYNVIVTAHAFVMIFFLVMPVMIGGFGNWLVPLMLGAPDMAFPRMNNMSFWLLPPSLTLLLSSAAVESGVGTGWTVYPPL
SSNLAHMGPSVDLAIFSLHLAGISSILGAINFITTIINMRWEGMLMERLPLFVWSVFITAILLLLSLPVLAGAITMLLTDRNFNTTFFDP
>ISLG490 COI
GTSLSLMIRTELGQPGSLLNDDQLYNVIVTAHAFVMIFFLVMPVMIGGFGNWLVPLMLGAPDMAFPRMNNMSFWLLPPSLTLLLSSAAVESGVGTGWTVYPPL
SSNLAHMGPSVDLAIFSLHLAGISSILGAINFITTIINMRWEGMLMERLPLFVWSVFITAILLLLSLPVLAGAITMLLTDRNFNTTFFDP
>ISLG489 COI
GTSLSLMIRTELGQPGSLLNDDQLYNVIVTAHAFVMIFFLVMPVMIGGFGNWLVPLMLGAPDMAFPRMNNMSFWLLPPSLTLLLSSAAVESGVGTGWTVYPPL
SSNLAHMGPSVDLAIFSLHLAGISSILGAINFITTIINMRWEGMLMERLPLFVWSVFITAILLLLSLPVLAGAITMLLTDRNFNTTFFDP
>ISLG487 CO
GTSLSLMIRTELGQPGSLLNDDQLYNVIVTAHAFVMIFFLVMPVMIGGFGNWLVPLMLGAPDMAFPRMNNMSFWLLPPSLTLLLSSAAVESGVGTGWTVYPPL
SSNLAHMGPSVDLAIFSLHLAGISSILGAINFITTIINMRWEGMLMERLPLFVWSVFITAILLLLSLPVLAGAITMLLTDRNFNTTFFDP
>ISLG486 COI
GTSLSLMIRTELGQPGSLLNDDQLYNVIVTAHAFVMIFFLVMPVMIGGFGNWLVPLMLGAPDMAFPRMNNMSFWLLPPSLTLLLSSAAVESGVGTGWTVYPPL
SSNLAHMGPSVDLAIFSLHLAGISSILGAINFITTIINMRWEGMLMERLPLFVWSVFITAILLLLSLPVLAGAITMLLTDRNFNTTFFDP
>ISLG485 COI
GTSLSLMIRTELGQPGSLLNDDQLYNVIVTAHAFVMIFFLVMPVMIGGFGNWLVPLMLGAPDMAFPRMNNMSFWLLPPSLTLLLSSAAVESGVGTGWTVYPPL
SSNLAHMGPSVDLAIFSLHLAGISSILGAINFITTIINMRWEGMLMERLPLFVWSVFITAILLLLSLPVLAGAITMLLTDRNFNTTFFDP
>ISLG484 COI
GTSLSLMIRTELGQPGSLLNDDQLYNVIVTAHAFVMIFFLVMPVMIGGFGNWLVPLMLGAPDMAFPRMNNMSFWLLPPSLTLLLSSAAVESGVGTGWTVYPPL

SSNLAHMGPSVDLAIFSLHLAGISSILGAINFITTIINMRWEGMLMERLPLFVWSVFITAILLLLSLPVLAGAITMLLTDRNFNTTFFDP
>ISLG483 COI
GTSLSLMIRTELGQPGSLLNDDQLYNVIVTAHAFVMIFFLVMPVMIGGFGNWLVPLMLGAPDMAFPRMNNMSFWLLPPSLTLLLSSAAVESGVGTGWTVYPPL
SSNLAHMGPSVDLAIFSLHLAGISSILGAINFITTIINMRWEGMLMERLPLFVWSVFITAILLLLSLPVLAGAITMLLTDRNFNTTFFDP
>ISL427 COI
GTSLSLMIRTELGQPGSLLNDDQLYNVIVTAHAFVMIFFLVMPVMIGGFGNWLVPLMLGAPDMAFPRMNNMSFWLLPPSLTLLLSSAAVESGAGTGWTVYPPL
SSNLAHMGPSVDLAIFSLHLAGISSILGAINFITTIINMRWEGMLMERLPLFVWSVFITAILLLLSLPVLAGAITMLLTDRNFNTTFFDP
>ISL426 COI
GTSLSLMIRTELGQPGSLLNDDQLYNVIVTAHAFVMIFFLVMPVMIGGFGNWLVPLMLGAPDMAFPRMNNMSFWLLPPSLTLLLSSAAVESGAGTGWTVYPPL
SSNLAHMGPSVDLAIFSLHLAGISSILGAINFITTIINMRWEGMLMERLPLFVWSVFITAILLLLSLPVLAGAITMLLTDRNFNTTFFDP
>ISL425 COI
GTSLSLMIRTELGQPGSLLNDDQLYNVIVTAHAFVMIFFLVMPVMIGGFGNWLVPLMLGAPDMAFPRMNNMSFWLLPPSLTLLLSSAAVESGAGTGWTVYPPL
SSNLAHMGPSVDLAIFSLHLAGISSILGAINFITTIINMRWEGMLMERLPLFVWSVFITAILLLLSLPVLAGAITMLLTDRNFNTTFFDP
>ISL424 COI
GTSLSLMIRTELGQPGSLLNDDQLYNVIVTAHAFVMIFFLVMPVMIGGFGNWLVPLMLGAPDMAFPRMNNMSFWLLPPSLTLLLSSAAVESGAGTGWTVYPPL
SSNLAHMGPSVDLAIFSLHLAGISSILGAINFITTIINMRWEGMLMERLPLFVWSVFITAILLLLSLPVLAGAITMLLTDRNFNTTFFDP
>ISL423 COI
GTSLSLMIRTELGQPGSLLNDDQLYNVIVTAHAFVMIFFLVMPVMIGGFGNWLVPLMLGAPDMAFPRMNNMSFWLLPPSLTLLLSSAAVESGAGTGWTVYPPL
SSNLAHMGPSVDLAIFSLHLAGISSILGAINFITTIINMRWEGMLMERLPLFVWSVFITAILLLLSLPVLAGAITMLLTDRNFNTTFFDP
>ISL422 COI
GTSLSLMIRTELGQPGSLLNDDQLYNVIVTAHAFVMIFFLVMPVMIGGFGNWLVPLMLGAPDMAFPRMNNMSFWLLPPSLTLLLSSAAVESGAGTGWTVYPPL
SSNLAHMGPSVDLAIFSLHLAGISSILGAINFITTIINMRWEGMLMERLPLFVWSVFITAILLLLSLPVLAGAITMLLTDRNFNTTFFDP
>ISL421 COI
GTSLSLMIRTELGQPGSLLNDDQLYNVIVTAHAFVMIFFLVMPVMIGGFGNWLVPLMLGAPDMAFPRMNNMSFWLLPPSLTLLLSSAAVESGAGTGWTVYPPL
SSNLAHMGPSVDLAIFSLHLAGISSILGAINFITTIINMRWEGMLMERLPLFVWSVFITAILLLLSLPVLAGAITMLLTDRNFNTTFFDP
>ISL420 COI
GTSLSLMIRTELGQPGSLLNDDQLYNVIVTAHAFVMIFFLVMPVMIGGFGNWLVPLMLGAPDMAFPRMNNMSFWLLPPSLTLLLSSAAVESGAGTGWTVYPPL
SSNLAHMGPSVDLAIFSLHLAGISSILGAINFITTIINMRWEGMLMERLPLFVWSVFITAILLLLSLPVLAGAITMLLTDRNFNTTFFDP
>EE82 COI
GTSLSLMIRTELGQPGSLLNDDQLYNVIVTAHAFVMIFFLVMPVMIGGFGNWLVPLMLGAPDMAFPRMNNMSFWLLPPSLTLLLSSAAVESGVGTGWTVYPPL
SSNLAHMGPSVDLAIFSLHLAGISSILGAINFITTIINMRWEGMLMERLPLFVWSVFITAILLLLSLPVLAGAITMLLTDRNFNTTFFDP
>EE81 COI
GTSLSLMIRTELGQPGSLLNDDQLYNVIVTAHAFVMIFFLVMPVMIGGFGNWLVPLMLGAPDMAFPRMNNMSFWLLPPSLTLLLSSAAVESGVGTGWTVYPPL
SSNLAHMGPSVDLAIFSLHLAGISSILGAINFITTIINMRWEGMLMERLPLFVWSVFITAILLLLSLPVLAGAITMLLTDRNFNTTFFDP
>EE80 COI
GTSLSLMIRTELGQPGSLLNDDQLYNVIVTAHAFVMIFFLVMPVMIGGFGNWLVPLMLGAPDMAFPRMNNMSFWLLPPSLTLLLSSAAVESGVGTGWTVYPPL
SSNLAHMGPSVDLAIFSLHLAGISSILGAINFITTIINMRWEGMLMERLPLFVWSVFITAILLLLSLPVLAGAITMLLTDRNFNTTFFDP
>EE79 COI
GTSLSLMIRTELGQPGSLLNDDQLYNVIVTAHAFVMIFFLVMPVMIGGFGNWLVPLMLGAPDMAFPRMNNMSFWLLPPSLTLLLSSAAVESGVGTGWTVYPPL
SSNLAHMGPSVDLAIFSLHLAGISSILGAINFITTIINMRWEGMLMERLPLFVWSVFITAILLLLSLPVLAGAITMLLTDRNFNTTFFDP
>EE77 COI
GTSLSLMIRTELGQPGSLLNDDQLYNVIVTAHAFVMIFFLVMPVMIGGFGNWLVPLMLGAPDMAFPRMNNMSFWLLPPSLTLLLSSAAVESGVGTGWTVYPPL
SSNLAHMGPSVDLAIFSLHLAGISSILGAINFITTIINMRWEGMLMERLPLFVWSVFITAILLLLSLPVLAGAITMLLTDRNFNTTFFDP
>EE76 COI
GTSLSLMIRTELGQPGSLLNDDQLYNVIVTAHAFVMIFFLVMPVMIGGFGNWLVPLMLGAPDMAFPRMNNMSFWLLPPSLTLLLSSAAVESGVGTGWTVYPPL
SSNLAHMGPSVDLAIFSLHLAGISSILGAINFITTIINMRWEGMLMERLPLFVWSVFITAILLLLSLPVLAGAITMLLTDRNFNTTFFDP
>EE75 COI
GTSLSLMIRTELGQPGSLLNDDQLYNVIVTAHAFVMIFFLVMPVMIGGFGNWLVPLMLGAPDMAFPRMNNMSFWLLPPSLTLLLSSAAVESGVGTGWTVYPPL
SSNLAHMGPSVDLAIFSLHLAGISSILGAINFITTIINMRWEGMLMERLPLFVWSVFITAILLLLSLPVLAGAITMLLTDRNFNTTFFDP
>BM23 COI
GTSLSLMIRTELGQPGSLLNDDQLYNVIVTAHAFVMIFFLVMPVMIGGFGNWLVPLMLGAPDMAFPRMNNMSFWLLPPSLTLLLSSAAVESGVGTGWTVYPPL
SSNLAHMGPSVDLAIFSLHLAGISSILGAINFITTIINMRWEGMLMERLPLFVWSVFITAILLLLSLPVLAGAITMLLTDRNFNTTFFDP
>BM22 COI
GTSLSLMIRTELGQPGSLLNDDQLYNVIVTAHAFVMIFFLVMPVMIGGFGNWLVPLMLGAPDMAFPRMNNMSFWLLPPSLTLLLSSAAVESGVGTGWTVYPPL
SSNLAHMGPSVDLAIFSLHLAGISSILGAINFITTIINMRWEGMLMERLPLFVWSVFITAILLLLSLPVLAGAITMLLTDRNFNTTFFDP
>BM21 COI
GTSLSLMIRTELGQPGSLLNDDQLYNVIVTAHAFVMIFFLVMPVMIGGFGNWLVPLMLGAPDMAFPRMNNMSFWLLPPSLTLLLSSAAVESGVGTGWTVYPPL
SSNLAHMGPSVDLAIFSLHLAGISSILGAINFITTIINMRWEGMLMERLPLFVWSVFITAILLLLSLPVLAGAITMLLTDRNFNTTFFDP
>BM20 COI
GTSLSLMIRTELGQPGSLLNDDQLYNVIVTAHAFVMIFFLVMPVMIGGFGNWLVPLMLGAPDMAFPRMNNMSFWLLPPSLTLLLSSAAVESGVGTGWTVYPPL
SSNLAHMGPSVDLAIFSLHLAGISSILGAINFITTIINMRWEGMLMERLPLFVWSVFITAILLLLSLPVLAGAITMLLTDRNFNTTFFDP
>BM19 COI
GTSLSLMIRTELGQPGSLLNDDQLYNVIVTAHAFVMIFFLVMPVMIGGFGNWLVPLMLGAPDMAFPRMNNMSFWLLPPSLTLLLSSAAVESGVGTGWTVYPPL
SSNLAHMGPSVDLAIFSLHLAGISSILGAINFITTIINMRWEGMLMERLPLFVWSVFITAILLLLSLPVLAGAITMLLTDRNFNTTFFDP
>BM18 COI
GTSLSLMIRTELGQPGSLLNDDQLYNVIVTAHAFVMIFFLVMPVMIGGFGNWLVPLMLGAPDMAFPRMNNMSFWLLPPSLTLLLSSAAVESGVGTGWTVYPPL
SSNLAHMGPSVDLAIFSLHLAGISSILGAINFITTIINMRWEGMLMERLPLFVWSVFITAILLLLSLPVLAGAITMLLTDRNFNTTFFDP
>BM17 COI
GTSLSLMIRTELGQPGSLLNDDQLYNVIVTAHAFVMIFFLVMPVMIGGFGNWLVPLMLGAPDMAFPRMNNMSFWLLPPSLTLLLSSAAVESGVGTGWTVYPPL
SSNLAHMGPSVDLAIFSLHLAGISSILGAINFITTIINMRWEGMLMERLPLFVWSVFITAILLLLSLPVLAGAITMLLTDRNFNTTFFDP
>BM16 COI
GTSLSLMIRTELGQPGSLLNDDQLYNVIVTAHAFVMIFFLVMPVMIGGFGNWLVPLMLGAPDMAFPRMNNMSFWLLPPSLTLLLSSAAVESGVGTGWTVYPPL
SSNLAHMGPSVDLAIFSLHLAGISSILGAINFITTIINMRWEGMLMERLPLFVWSVFITAILLLLSLPVLAGAITMLLTDRNFNTTFFDP
>BLA290 COI
GTSLSLMIRTELGQPGSLLNDDQLYNVIVTAHAFVMIFFLVMPVMIGGFGNWLVPLMLGAPDMAFPRMNNMSFWLLPPSLTLLLSSAAVESGVGTGWTVYPPL
SSNLAHMGPSVDLAIFSLHLAGISSILGAINFITTIINMRWEGMLMERLPLFVWSVFITAILLLLSLPVLAGAITMLLTDRNFNTTFFDP
>BLA289 COI
GTSLSLMIRTELGQPGSLLNDDQLYNVIVTAHAFVMIFFLVMPVMIGGFGNWLVPLMLGAPDMAFPRMNNMSFWLLPPSLTLLLSSAAVESGVGTGWTVYPPL
SSNLAHMGPSVDLAIFSLHLAGISSILGAINFITTIINMRWEGMLMERLPLFVWSVFITAILLLLSLPVLAGAITMLLTDRNFNTTFFDP

>BLA288 COI
GTSLSLMIRTELGQPGSLLNDDQLYNVIVTAHAFVMIFFLVMPVMIGGFGNWLVPLMLGAPDMAFPRMNNMSFWLLPPSLTLLLSSAAVESGVGTGWTVYPPL
SSNLAHMGPSVDLAIFSLHLAGISSILGAINFITTIINMRWEGMLMERLPLFVWSVFITAILLLLSLPVLAGAITMLLTDRNFNTTFFDP
>BLA287 COI
GTSLSLMIRTELGQPGSLLNDDQLYNVIVTAHAFVMIFFLVMPVMIGGFGNWLVPLMLGAPDMAFPRMNNMSFWLLPPSLTLLLSSAAVESGVGTGWTVYPPL
SSNLAHMGPSVDLAIFSLHLAGISSILGAINFITTIINMRWEGMLMERLPLFVWSVFITAILLLLSLPVLAGAITMLLTDRNFNTTFFDP
>BLA286 COI
GTSLSLMIRTELGQPGSLLNDDQLYNVIVTAHAFVMIFFLVMPVMIGGFGNWLVPLMLGAPDMAFPRMNNMSFWLLPPSLTLLLSSAAVESGVGTGWTVYPPL
SSNLAHMGPSVDLAIFSLHLAGISSILGAINFITTIINMRWEGMLMERLPLFVWSVFITAILLLLSLPVLAGAITMLLTDRNFNTTFFDP
>BLA285 COI
GTSLSLMIRTELGQPGSLLNDDQLYNVIVTAHAFVMIFFLVMPVMIGGFGNWLVPLMLGAPDMAFPRMNNMSFWLLPPSLTLLLSSAAVESGVGTGWTVYPPL
SSNLAHMGPSVDLAIFSLHLAGISSILGAINFITTIINMRWEGMLMERLPLFVWSVFITAILLLLSLPVLAGAITMLLTDRNFNTTFFDP
>BLA284 COI
GTSLSLMIRTELGQPGSLLNDDQLYNVIVTAHAFVMIFFLVMPVMIGGFGNWLVPLMLGAPDMAFPRMNNMSFWLLPPSLTLLLSSAAVESGVGTGWTVYPPL
SSNLAHMGPSVDLAIFSLHLAGISSILGAINFITTIINMRWEGMLMERLPLFVWSVFITAILLLLSLPVLAGAITMLLTDRNFNTTFFDP
>BLA283 COI
GTSLSLMIRTELGQPGSLLNDDQLYNVIVTAHAFVMIFFLVMPVMIGGFGNWLVPLMLGAPDMAFPRMNNMSFWLLPPSLTLLLSSAAVESGVGTGWTVYPPL
SSNLAHMGPSVDLAIFSLHLAGISSILGAINFITTIINMRWEGMLMERLPLFVWSVFITAILLLLSLPVLAGAITMLLTDRNFNTTFFDP
>BKI395 COI
GTSLSLMIRTELGQPGSLLNDDQLYNVIVTAHAFVMIFFLVMPVMIGGFGNWLVPLMLGAPDMAFPRMNNMSFWLLPPSLTLLLSSAAVESGAGTGWTVYPPL
SSNLAHMGPSVDLAIFSLHLAGISSILGAINFITTIINMRWEGMLMERLPLFVWSVFITAILLLLSLPVLAGAITMLLTDRNFNTTFFDP
>BKI394 COI
GTSLSLMIRTELGQPGSLLNDDQLYNVIVTAHAFVMIFFLVMPVMIGGFGNWLVPLMLGAPDMAFPRMNNMSFWLLPPSLTLLLSSAAVESGAGTGWTVYPPL
SSNLAHMGPSVDLAIFSLHLAGISSILGAINFITTIINMRWEGMLMERLPLFVWSVFITAILLLLSLPVLAGAITMLLTDRNFNTTFFDP
>BKI393 COI
GTSLSLMIRTELGQPGSLLNDDQLYNVIVTAHAFVMIFFLVMPVMIGGFGNWLVPLMLGAPDMAFPRMNNMSFWLLPPSLTLLLSSAAVESGAGTGWTVYPPL
SSNLAHMGPSVDLAIFSLHLAGISSILGAINFITTIINMRWEGMLMERLPLFVWSVFITAILLLLSLPVLAGAITMLLTDRNFNTTFFDP
>BKI392 COI
GTSLSLMIRTELGQPGSLLNDDQLYNVIVTAHAFVMIFFLVMPVMIGGFGNWLVPLMLGAPDMAFPRMNNMSFWLLPPSLTLLLSSAAVESGAGTGWTVYPPL
SSNLAHMGPSVDLAIFSLHLAGISSILGAINFITTIINMRWEGMLMERLPLFVWSVFITAILLLLSLPVLAGAITMLLTDRNFNTTFFDP
>BKI391 COI
GTSLSLMIRTELGQPGSLLNDDQLYNVIVTAHAFVMIFFLVMPVMIGGFGNWLVPLMLGAPDMAFPRMNNMSFWLLPPSLTLLLSSAAVESGAGTGWTVYPPL
SSNLAHMGPSVDLAIFSLHLAGISSILGAINFITTIINMRWEGMLMERLPLFVWSVFITAILLLLSLPVLAGAITMLLTDRNFNTTFFDP
>BKI390 COI
GTSLSLMIRTELGQPGSLLNDDQLYNVIVTAHAFVMIFFLVMPVMIGGFGNWLVPLMLGAPDMAFPRMNNMSFWLLPPSLTLLLSSAAVESGAGTGWTVYPPL
SSNLAHMGPSVDLAIFSLHLAGISSILGAINFITTIINMRWEGMLMERLPLFVWSVFITAILLLLSLPVLAGAITMLLTDRNFNTTFFDP
>BKI389 COI
GTSLSLMIRTELGQPGSLLNDDQLYNVIVTAHAFVMIFFLVMPVMIGGFGNWLVPLMLGAPDMAFPRMNNMSFWLLPPSLTLLLSSAAVESGAGTGWTVYPPL
SSNLAHMGPSVDLAIFSLHLAGISSILGAINFITTIINMRWEGMLMERLPLFVWSVFITAILLLLSLPVLAGAITMLLTDRNFNTTFFDP
>BKI388 COI
GTSLSLMIRTELGQPGSLLNDDQLYNVIVTAHAFVMIFFLVMPVMIGGFGNWLVPLMLGAPDMAFPRMNNMSFWLLPPSLTLLLSSAAVESGAGTGWTVYPPL
SSNLAHMGPSVDLAIFSLHLAGISSILGAINFITTIINMRWEGMLMERLPLFVWSVFITAILLLLSLPVLAGAITMLLTDRNFNTTFFDP
