# Supplementary material for: Life histories predict genetic diversity and population structure within three species of octopus targeted by small-scale fisheries in Northwest Mexico
Source: PeerJ. 2018 Feb 15;6:e4295. doi: 10.7717/peerj.4295 (PMC5816968; doi:10.7717/peerj.4295)
Supplement: Supplemental Information 3 [file peerj-06-4295-s010.pdf]

>SQ9616s
CCAAAAACATGTCTCTTTGAGTTTTTTTAATAAAGAGTTGGGCCTGCTCGGTGATTAATATTTAACAGCTGCGGTATTATAACTGTACTAAGGTAGCATAGTA
ATTTGCTCTATAAATTGGGGCTAGAATGAATGGTTTGACGAAAATTTAACTGTCTCTATTTTATTTATTAGAAATTAATTTTTATAGTGAAAAAGCTTAAATT
ATTTAAAGGGACGAAAAGACCCTATTGAGCTTATATTATTTATTAAATTATATAGTTATAGTTTATTATAAATTAATTTTGATTGGGGTGATCAAGGAATAAA
TTTTATTATTATATAACTTCCTTAGTTAAAATAGTTTTTGGAAAAATAAACCAAGTTTTTTGCTTAGAAGATAAGTTACCATAGGGATAACAGCGTAATTTTT
TTTGAGAGTTCATATTGAAAAAGGAGATTGCGACCTCGATGTTGGATTAAAATTAACCTTA
>SQ9516s
CCAAAAACATGTCTCTTTGAGTTTTTTTAATAAAGAGTTGGGCCTGCTCGGTGATTAATATTTAACAGCTGCGGTATTATAACTGTACTAAGGTAGCATAGTA
ATTTGCTCTATAAATTGGGGCTAGAATGAATGGTTTGACGAAAATTTAACTGTCTCTATTTTATTTATTAGAAATTAATTTTTATAGTGAAAAAGCTTAAATT
ATTTAAAGGGACGAAAAGACCCTATTGAGCTTATATTATTTATTAAATTATATAGTTATAGTTTATTATAAATTAATTTTGATTGGGGTGATCAAGGAATAAA
TTTTATTATTATATAACTTCCTTAGTTAAAATAGTTTTTGGAAAAATAAACCAAGTTTTTTGCTTAGAAGATAAGTTACCATAGGGATAACAGCGTAATTTTT
TTTGAGAGTTCATATTGAAAAAGGAGATTGCGACCTCGATGTTGGATTAAAATTAACCTTA
>SQ9416s
CCAAAAACATGTCTCTTTGAGTTTTTTTAATAAAGAGTTGGGCCTGCTCGGTGATTAATATTTAACAGCTGCGGTATTATAACTGTACTAAGGTAGCATAGTA
ATTTGCTCTATAAATTGGGGCTAGAATGAATGGTTTGACGAAAATTTAACTGTCTCTATTTTATTTATTAGAAATTAATTTTTATAGTGAAAAAGCTTAAATT
ATTTAAAGGGACGAAAAGACCCTATTGAGCTTATATTATTTATTAAATTATATAGTTATAGTTTATTATAAATTAATTTTGATTGGGGTGATCAAGGAATAAA
TTTTATTATTATATAACTTCCTTAGTTAAAATAGTTTTTGGAAAAATAAACCAAGTTTTTTGCTTAGAAGATAAGTTACCATAGGGATAACAGCGTAATTTTT
TTTGAGAGTTCATATTGAAAAAGGAGATTGCGACCTCGATGTTGGATTAAAATTAACCTTA
>SQ9316s
CCAAAAACATGTCTCTTTGAGTTTTTTTAATAAAGAGTTGGGCCTGCTCGGTGATTAATATTTAACAGCTGCGGTATTATAACTGTACTAAGGTAGCATAGTA
ATTTGCTCTATAAATTGGGGCTAGAATGAATGGTTTGACGAAAATTTAACTGTCTCTATTTTATTTATTAGAAATTAATTTTTATAGTGAAAAAGCTTAAATT
ATTTAAAGGGACGAAAAGACCCTATTGAGCTTATATTATTTATTAAATTATATAGTTATAGTTTATTATAAATTAATTTTGATTGGGGTGATCAAGGAATAAA
TTTTATTATTATATAACTTCCTTAGTTAAAATAGTTTTTGGAAAAATAAACCAAGTTTTTTGCTTAGAAGATAAGTTACCATAGGGATAACAGCGTAATTTTT
TTTGAGAGTTCATATTGAAAAAGGAGATTGCGACCTCGATGTTGGATTAAAATTAACCTTA
>SQ9216s
CCAAAAACATGTCTCTTTGAGTTTTTTTAATAAAGAGTTGGGCCTGCTCGGTGATTAATATTTAACAGCTGCGGTATTATAACTGTACTAAGGTAGCATAGTA
ATTTGCTCTATAAATTGGGGCTAGAATGAATGGTTTGACGAAAATTTAACTGTCTCTATTTTATTTATTAGAAATTAATTTTTATAGTGAAAAAGCTTAAATT
ATTTAAAGGGACGAAAAGACCCTATTGAGCTTATATTATTTATTAAATTATATAGTTATAGTTTATTATAAATTAATTTTGATTGGGGTGATCAAGGAATAAA
TTTTATTATTATATAACTTCCTTAGTTAAAATAGTTTTTGGAAAAATAAACCAAGTTTTTTGCTTAGAAGATAAGTTACCATAGGGATAACAGCGTAATTTTT
TTTGAGAGTTCATATTGAAAAAGGAGATTGCGACCTCGATGTTGGATTAAAATTAACCTTA
>SQ9116s
CCAAAAACATGTCTCTTTGAGTTTTTTTAATAAAGAGTTGGGCCTGCTCGGTGATTAATATTTAACAGCTGCGGTATTATAACTGTACTAAGGTAGCATAGTA
ATTTGCTCTATAAATTGGGGCTAGAATGAATGGTTTGACGAAAATTTAACTGTCTCTATTTTATTTATTAGAAATTAATTTTTATAGTGAAAAAGCTTAAATT
ATTTAAAGGGACGAAAAGACCCTATTGAGCTTATATTATTTATTAAATTATATAGTTATAGTTTATTATAAATTAATTTTGATTGGGGTGATCAAGGAATAAA
TTTTATTATTATATAACTTCCTTAGTTAAAATAGTTTTTGGAAAAATAAACCAAGTTTTTTGCTTAGAAGATAAGTTACCATAGGGATAACAGCGTAATTTTT
TTTGAGAGTTCATATTGAAAAAGGAGATTGCGACCTCGATGTTGGATTAAAATTAACCTTA
>SQ9016s
CCAAAAACATGTCTCTTTGAGTTTTTTTAATAAAGAGTTGGGCCTGCTCGGTGATTAATATTTAACAGCTGCGGTATTATAACTGTACTAAGGTAGCATAGTA
ATTTGCTCTATAAATTGGGGCTAGAATGAATGGTTTGACGAAAATTTAACTGTCTCTATTTTATTTATTAGAAATTAATTTTTATAGTGAAAAAGCTTAAATT
ATTTAAAGGGACGAAAAGACCCTATTGAGCTTATATTATTTATTAAATTATATAGTTATAGTTTATTATAAATTAATTTTGATTGGGGTGATCAAGGAATAAA
TTTTATTATTATATAACTTCCTTAGTTAAAATAGTTTTTGGAAAAATAAACCAAGTTTTTTGCTTAGAAGATAAGTTACCATAGGGATAACAGCGTAATTTTT
TTTGAGAGTTCATATTGAAAAAGGAGATTGCGACCTCGATGTTGGATTAAAATTAACCTTA
>SQ8916s
CCAAAAACATGTCTCTTTGAGTTTTTTTAATAAAGAGTTGGGCCTGCTCGGTGATTAATATTTAACAGCTGCGGTATTATAACTGTACTAAGGTAGCATAGTA
ATTTGCTCTATAAATTGGGGCTAGAATGAATGGTTTGACGAAAATTTAACTGTCTCTATTTTATTTATTAGAAATTAATTTTTATAGTGAAAAAGCTTAAATT
ATTTAAAGGGACGAAAAGACCCTATTGAGCTTATATTATTTATTAAATTATATAGTTATAGTTTATTATAAATTAATTTTGATTGGGGTGATCAAGGAATAAA
TTTTATTATTATATAACTTCCTTAGTTAAAATAGTTTTTGGAAAAATAAACCAAGTTTTTTGCTTAGAAGATAAGTTACCATAGGGATAACAGCGTAATTTTT
TTTGAGAGTTCATATTGAAAAAGGAGATTGCGACCTCGATGTTGGATTAAAATTAACCTTA
>BM1616s
CCAAAAACATGTCTCTTTGAGTTTTTTTAATAAAGAGTTGGGCCTGCTCGGTGATTAATATTTAACAGCTGCGGTATTATAACTGTACTAAGGTAGCATAGTA
ATTTGCTCTATAAATTGGGGCTAGAATGAATGGTTTGACGAAAATTTAACTGTCTCTATTTTATTTATTAGAAATTAATTTTTATAGTGAAAAAGCTTAAATT
ATTTAAAGGGACGAAAAGACCCTATTGAGCTTATATTATTTATTAAATTATATAGTTATAGTTTATTATAAATTAATTTTGATTGGGGTGATCAAGGAATAAA
TTTTATTATTATATAACTTCCTTAGTTAAAATAGTTTTTGGAAAAATAAACCAAGTTTTTTGCTTAGAAGATAAGTTACCATAGGGATAACAGCGTAATTTTT
TTTGAGAGTTCATATTGAAAAAGGAGATTGCGACCTCGATGTTGGATTAAAATTAACCTTA
>BM1716s
CCAAAAACATGTCTCTTTGAGTTTTTTTAATAAAGAGTTGGGCCTGCTCGGTGATTAATATTTAACAGCTGCGGTATTATAACTGTACTAAGGTAGCATAGTA
ATTTGCTCTATAAATTGGGGCTAGAATGAATGGTTTGACGAAAATTTAACTGTCTCTATTTTATTTATTAGAAATTAATTTTTATAGTGAAAAAGCTTAAATT
ATTTAAAGGGACGAAAAGACCCTATTGAGCTTATATTATTTATTAAATTATATAGTTATAGTTTATTATAAATTAATTTTGATTGGGGTGATCAAGGAATAAA
TTTTATTATTATATAACTTCCTTAGTTAAAATAGTTTTTGGAAAAATAAACCAAGTTTTTTGCTTAGAAGATAAGTTACCATAGGGATAACAGCGTAATTTTT
TTTGAGAGTTCATATTGAAAAAGGAGATTGCGACCTCGATGTTGGATTAAAATTAACCTTA
>BM1816s
CCAAAAACATGTCTCTTTGAGTTTTTTTAATAAAGAGTTGGGCCTGCTCGGTGATTAATATTTAACAGCTGCGGTATTATAACTGTACTAAGGTAGCATAGTA
ATTTGCTCTATAAATTGGGGCTAGAATGAATGGTTTGACGAAAATTTAACTGTCTCTATTTTATTTATTAGAAATTAATTTTTATAGTGAAAAAGCTTAAATT
ATTTAAAGGGACGAAAAGACCCTATTGAGCTTATATTATTTATTAAATTATATAGTTATAGTTTATTATAAATTAATTTTGATTGGGGTGATCAAGGAATAAA
TTTTATTATTATATAACTTCCTTAGTTAAAATAGTTTTTGGAAAAATAAACCAAGTTTTTTGCTTAGAAGATAAGTTACCATAGGGATAACAGCGTAATTTTT
TTTGAGAGTTCATATTGAAAAAGGAGATTGCGACCTCGATGTTGGATTAAAATTAACCTTA
>BM1916s
CCAAAAACATGTCTCTTTGAGTTTTTTTAATAAAGAGTTGGGCCTGCTCGGTGATTAATATTTAACAGCTGCGGTATTATAACTGTACTAAGGTAGCATAGTA
ATTTGCTCTATAAATTGGGGCTAGAATGAATGGTTTGACGAAAATTTAACTGTCTCTATTTTATTTATTAGAAATTAATTTTTATAGTGAAAAAGCTTAAATT
ATTTAAAGGGACGAAAAGACCCTATTGAGCTTATATTATTTATTAAATTATATAGTTATAGTTTATTATAAATTAATTTTGATTGGGGTGATCAAGGAATAAA
TTTTATTATTATATAACTTCCTTAGTTAAAATAGTTTTTGGAAAAATAAACCAAGTTTTTTGCTTAGAAGATAAGTTACCATAGGGATAACAGCGTAATTTTT
TTTGAGAGTTCATATTGAAAAAGGAGATTGCGACCTCGATGTTGGATTAAAATTAACCTTA
>BM2016s
CCAAAAACATGTCTCTTTGAGTTTTTTTAATAAAGAGTTGGGCCTGCTCGGTGATTAATATTTAACAGCTGCGGTATTATAACTGTACTAAGGTAGCATAGTA
ATTTGCTCTATAAATTGGGGCTAGAATGAATGGTTTGACGAAAATTTAACTGTCTCTATTTTATTTATTAGAAATTAATTTTTATAGTGAAAAAGCTTAAATT
ATTTAAAGGGACGAAAAGACCCTATTGAGCTTATATTATTTATTAAATTATATAGTTATAGTTTATTATAAATTAATTTTGATTGGGGTGATCAAGGAATAAA
TTTTATTATTATATAACTTCCTTAGTTAAAATAGTTTTTGGAAAAATAAACCAAGTTTTTTGCTTAGAAGATAAGTTACCATAGGGATAACAGCGTAATTTTT
TTTGAGAGTTCATATTGAAAAAGGAGATTGCGACCTCGATGTTGGATTAAAATTAACCTTA
>BM2116s

CCAAAAACATGTCTCTTTGAGTTTTTTTAATAAAGAGTTGGGCCTGCTCGGTGATTAATATTTAACAGCTGCGGTATTATAACTGTACTAAGGTAGCATAGTA
ATTTGCTCTATAAATTGGGGCTAGAATGAATGGTTTGACGAAAATTTAACTGTCTCTATTTTATTTATTAGAAATTAATTTTTATAGTGAAAAAGCTTAAATT
ATTTAAAGGGACGAAAAGACCCTATTGAGCTTATATTATTTATTAAATTATATAGTTATAGTTTATTATAAATTAATTTTGATTGGGGTGATCAAGGAATAAA
TTTTATTATTATATAACTTCCTTAGTTAAAATAGTTTTTGGAAAAATAAACCAAGTTTTTTGCTTAGAAGATAAGTTACCATAGGGATAACAGCGTAATTTTT
TTTGAGAGTTCATATTGAAAAAGGAGATTGCGACCTCGATGTTGGATTAAAATTAACCTTA
>BM2216s
CCAAAAACATGTCTCTTTGAGTTTTTTTAATAAAGAGTTGGGCCTGCTCGGTGATTAATATTTAACAGCTGCGGTATTATAACTGTACTAAGGTAGCATAGTA
ATTTGCTCTATAAATTGGGGCTAGAATGAATGGTTTGACGAAAATTTAACTGTCTCTATTTTATTTATTAGAAATTAATTTTTATAGTGAAAAAGCTTAAATT
ATTTAAAGGGACGAAAAGACCCTATTGAGCTTATATTATTTATTAAATTATATAGTTATAGTTTATTATAAATTAATTTTGATTGGGGTGATCAAGGAATAAA
TTTTATTATTATATAACTTCCTTAGTTAAAATAGTTTTTGGAAAAATAAACCAAGTTTTTTGCTTAGAAGATAAGTTACCATAGGGATAACAGCGTAATTTTT
TTTGAGAGTTCATATTGAAAAAGGAGATTGCGACCTCGATGTTGGATTAAAATTAACCTTA
>BM2316s
CCAAAAACATGTCTCTTTGAGTTTTTTTAATAAAGAGTTGGGCCTGCTCGGTGATTAATATTTAACAGCTGCGGTATTATAACTGTACTAAGGTAGCATAGTA
ATTTGCTCTATAAATTGGGGCTAGAATGAATGGTTTGACGAAAATTTAACTGTCTCTATTTTATTTATTAGAAATTAATTTTTATAGTGAAAAAGCTTAAATT
ATTTAAAGGGACGAAAAGACCCTATTGAGCTTATATTATTTATTAAATTATATAGTTATAGTTTATTATAAATTAATTTTGATTGGGGTGATCAAGGAATAAA
TTTTATTATTATATAACTTCCTTAGTTAAAATAGTTTTTGGAAAAATAAACCAAGTTTTTTGCTTAGAAGATAAGTTACCATAGGGATAACAGCGTAATTTTT
TTTGAGAGTTCATATTGAAAAAGGAGATTGCGACCTCGATGTTGGATTAAAATTAACCTTA
>EE7516s
CCAAAAACATGTCTCTTTGAGTTTTTTTAATAAAGAGTTGGGCCTGCTCGGTGATTAATATTTAACAGCTGCGGTATTATAACTGTACTAAGGTAGCATAGTA
ATTTGCTCTATAAATTGGGGCTAGAATGAATGGTTTGACGAAAATTTAACTGTCTCTATTTTATTTATTAGAAATTAATTTTTATAGTGAAAAAGCTTAAATT
ATTTAAAGGGACGAAAAGACCCTATTGAGCTTATATTATTTATTAAATTATATAGTTATAGTTTATTATAAATTAATTTTGATTGGGGTGATCAAGGAATAAA
TTTTATTATTATATAACTTCCTTAGTTAAAATAGTTTTTGGAAAAATAAACCAAGTTTTTTGCTTAGAAGATAAGTTACCATAGGGATAACAGCGTAATTTTT
TTTGAGAGTTCATATTGAAAAAGGAGATTGCGACCTCGATGTTGGATTAAAATTAACCTTA
>EE7616s
CCAAAAACATGTCTCTTTGAGTTTTTTTAATAAAGAGTTGGGCCTGCTCGGTGATTAATATTTAACAGCTGCGGTATTATAACTGTACTAAGGTAGCATAGTA
ATTTGCTCTATAAATTGGGGCTAGAATGAATGGTTTGACGAAAATTTAACTGTCTCTATTTTATTTATTAGAAATTAATTTTTATAGTGAAAAAGCTTAAATT
ATTTAAAGGGACGAAAAGACCCTATTGAGCTTATATTATTTATTAAATTATATAGTTATAGTTTATTATAAATTAATTTTGATTGGGGTGATCAAGGAATAAA
TTTTATTATTATATAACTTCCTTAGTTAAAATAGTTTTTGGAAAAATAAACCAAGTTTTTTGCTTAGAAGATAAGTTACCATAGGGATAACAGCGTAATTTTT
TTTGAGAGTTCATATTGAAAAAGGAGATTGCGACCTCGATGTTGGATTAAAATTAACCTTA
>EE7716s
CCAAAAACATGTCTCTTTGAGTTTTTTTAATAAAGAGTTGGGCCTGCTCGGTGATTAATATTTAACAGCTGCGGTATTATAACTGTACTAAGGTAGCATAGTA
ATTTGCTCTATAAATTGGGGCTAGAATGAATGGTTTGACGAAAATTTAACTGTCTCTATTTTATTTATTAGAAATTAATTTTTATAGTGAAAAAGCTTAAATT
ATTTAAAGGGACGAAAAGACCCTATTGAGCTTATATTATTTATTAAATTATATAGTTATAGTTTATTATAAATTAATTTTGATTGGGGTGATCAAGGAATAAA
TTTTATTATTATATAACTTCCTTAGTTAAAATAGTTTTTGGAAAAATAAACCAAGTTTTTTGCTTAGAAGATAAGTTACCATAGGGATAACAGCGTAATTTTT
TTTGAGAGTTCATATTGAAAAAGGAGATTGCGACCTCGATGTTGGATTAAAATTAACCTTA
>EE7816s
CCAAAAACATGTCTCTTTGAGTTTTTTTAATAAAGAGTTGGGCCTGCTCGGTGATTAATATTTAACAGCTGCGGTATTATAACTGTACTAAGGTAGCATAGTA
ATTTGCTCTATAAATTGGGGCTAGAATGAATGGTTTGACGAAAATTTAACTGTCTCTATTTTATTTATTAGAAATTAATTTTTATAGTGAAAAAGCTTAAATT
ATTTAAAGGGACGAAAAGACCCTATTGAGCTTATATTATTTATTAAATTATATAGTTATAGTTTATTATAAATTAATTTTGATTGGGGTGATCAAGGAATAAA
TTTTATTATTATATAACTTCCTTAGTTAAAATAGTTTTTGGAAAAATAAACCAAGTTTTTTGCTTAGAAGATAAGTTACCATAGGGATAACAGCGTAATTTTT
TTTGAGAGTTCATATTGAAAAAGGAGATTGCGACCTCGATGTTGGATTAAAATTAACCTTA
>EE7916s
CCAAAAACATGTCTCTTTGAGTTTTTTTAATAAAGAGTTGGGCCTGCTCGGTGATTAATATTTAACAGCTGCGGTATTATAACTGTACTAAGGTAGCATAGTA
ATTTGCTCTATAAATTGGGGCTAGAATGAATGGTTTGACGAAAATTTAACTGTCTCTATTTTATTTATTAGAAATTAATTTTTATAGTGAAAAAGCTTAAATT
ATTTAAAGGGACGAAAAGACCCTATTGAGCTTATATTATTTATTAAATTATATAGTTATAGTTTATTATAAATTAATTTTGATTGGGGTGATCAAGGAATAAA
TTTTATTATTATATAACTTCCTTAGTTAAAATAGTTTTTGGAAAAATAAACCAAGTTTTTTGCTTAGAAGATAAGTTACCATAGGGATAACAGCGTAATTTTT
TTTGAGAGTTCATATTGAAAAAGGAGATTGCGACCTCGATGTTGGATTAAAATTAACCTTA
>EE8016s
CCAAAAACATGTCTCTTTGAGTTTTTTTAATAAAGAGTTGGGCCTGCTCGGTGATTAATATTTAACAGCTGCGGTATTATAACTGTACTAAGGTAGCATAGTA
ATTTGCTCTATAAATTGGGGCTAGAATGAATGGTTTGACGAAAATTTAACTGTCTCTATTTTATTTATTAGAAATTAATTTTTATAGTGAAAAAGCTTAAATT
ATTTAAAGGGACGAAAAGACCCTATTGAGCTTATATTATTTATTAAATTATATAGTTATAGTTTATTATAAATTAATTTTGATTGGGGTGATCAAGGAATAAA
TTTTATTATTATATAACTTCCTTAGTTAAAATAGTTTTTGGAAAAATAAACCAAGTTTTTTGCTTAGAAGATAAGTTACCATAGGGATAACAGCGTAATTTTT
TTTGAGAGTTCATATTGAAAAAGGAGATTGCGACCTCGATGTTGGATTAAAATTAACCTTA
>EE8116s
CCAAAAACATGTCTCTTTGAGTTTTTTTAATAAAGAGTTGGGCCTGCTCGGTGATTAATATTTAACAGCTGCGGTATTATAACTGTACTAAGGTAGCATAGTA
ATTTGCTCTATAAATTGGGGCTAGAATGAATGGTTTGACGAAAATTTAACTGTCTCTATTTTATTTATTAGAAATTAATTTTTATAGTGAAAAAGCTTAAATT
ATTTAAAGGGACGAAAAGACCCTATTGAGCTTATATTATTTATTAAATTATATAGTTATAGTTTATTATAAATTAATTTTGATTGGGGTGATCAAGGAATAAA
TTTTATTATTATATAACTTCCTTAGTTAAAATAGTTTTTGGAAAAATAAACCAAGTTTTTTGCTTAGAAGATAAGTTACCATAGGGATAACAGCGTAATTTTT
TTTGAGAGTTCATATTGAAAAAGGAGATTGCGACCTCGATGTTGGATTAAAATTAACCTTA
>EE8216s
CCAAAAACATGTCTCTTTGAGTTTTTTTAATAAAGAGTTGGGCCTGCTCGGTGATTAATATTTAACAGCTGCGGTATTATAACTGTACTAAGGTAGCATAGTA
ATTTGCTCTATAAATTGGGGCTAGAATGAATGGTTTGACGAAAATTTAACTGTCTCTATTTTATTTATTAGAAATTAATTTTTATAGTGAAAAAGCTTAAATT
ATTTAAAGGGACGAAAAGACCCTATTGAGCTTATATTATTTATTAAATTATATAGTTATAGTTTATTATAAATTAATTTTGATTGGGGTGATCAAGGAATAAA
TTTTATTATTATATAACTTCCTTAGTTAAAATAGTTTTTGGAAAAATAAACCAAGTTTTTTGCTTAGAAGATAAGTTACCATAGGGATAACAGCGTAATTTTT
TTTGAGAGTTCATATTGAAAAAGGAGATTGCGACCTCGATGTTGGATTAAAATTAACCTTA
>BLA28316s
CCAAAAACATGTCTCTTTGAGTTTTTTTAATAAAGAGTTGGGCCTGCTCGGTGATTAATATTTAACAGCTGCGGTATTATAACTGTACTAAGGTAGCATAATA
ATTTGCTCTATAAATTGGGGCTAGAATGAATGGTTTGACGAAAATTTAACTGTCTCTATTTTATTTATTAGAAATTAATTTTTATAGTGAGAAAGCTTAAATT
GTTTAAAGGGACGAAAAGACCCTATTGAGCTTATATTATTTATTAAATTATAGAGTTATAATTTATTATAAATTAATTTTGGTTGGGGTGATCAAGGAATAAG
TTTAATGATTATATAACTTCCTTAACTGAACTATTTTTTGGAAAAATAAACCAAGTTTTTTGCTTAGAAGATAAGTTACCATAGGGATAACAGCGTAATTTTT
TTTGAGAGTTCGTATTGAAAAAAGAGATTGCGACCTCGATGTTGGATTAAAATTAACCTTA
>BLA28516s
CCAAAAACATGTCTCTTTGAGTTTTTTTAATAAAGAGTTGGGCCTGCTCGGTGATTAATATTTAACAGCTGCGGTATTATAACTGTACTAAGGTAGCATAATA
ATTTGCTCTATAAATTGGGGCTAGAATGAATGGTTTGACGAAAATTTAACTGTCTCTATTTTATTTATTAGAAATTAATTTTTATAGTGAGAAAGCTTAAATT
GTTTAAAGGGACGAAAAGACCCTATTGAGCTTATATTATTTATTAAATTATAGAGTTATAATTTATTATAAATTAATTTTGGTTGGGGTGATCAAGGAATAAG
TTTAATGATTATATAACTTCCTTAACTGAACTATTTTTTGGAAAAATAAACCAAGTTTTTTGCTTAGAAGATAAGTTACCATAGGGATAACAGCGTAATTTTT
TTTGAGAGTTCGTATTGAAAAAAGAGATTGCGACCTCGATGTTGGATTAAAATTAACCTTA
>BLA28616s
CCAAAAACATGTCTCTTTGAGTTTTTTTAATAAAGAGTTGGGCCTGCTCGGTGATTAATATTTAACAGCTGCGGTATTATAACTGTACTAAGGTAGCATAATA

ATTTGCTCTATAAATTGGGGCTAGAATGAATGGTTTGACGAAAATTTAACTGTCTCTATTTTATTTATTAGAAATTAATTTTTATAGTGAGAAAGCTTAAATT
GTTTAAAGGGACGAAAAGACCCTATTGAGCTTATATTATTTATTAAATTATAGAGTTATAATTTATTATAAATTAATTTTGGTTGGGGTGATCAAGGAATAAG
TTTAATGATTATATAACTTCCTTAACTGAACTATTTTTTGGAAAAATAAACCAAGTTTTTTGCTTAGAAGATAAGTTACCATAGGGATAACAGCGTAATTTTT
TTTGAGAGTTCGTATTGAAAAAAGAGATTGCGACCTCGATGTTGGATTAAAATTAACCTTA
>BLA28716s
CCAAAAACATGTCTCTTTGAGTTTTTTTAATAAAGAGTTGGGCCTGCTCGGTGATTAATATTTAACAGCTGCGGTATTATAACTGTACTAAGGTAGCATAATA
ATTTGCTCTATAAATTGGGGCTAGAATGAATGGTTTGACGAAAATTTAACTGTCTCTATTTTATTTATTAGAAATTAATTTTTATAGTGAGAAAGCTTAAATT
GTTTAAAGGGACGAAAAGACCCTATTGAGCTTATATTATTTATTAAATTATAGAGTTATAATTTATTGTAAATTAATTTTGGTTGGGGTGATCAAGGAATAAG
TTTAATGATTATATAACTTCCTTAACTGAACTATTTTTTGGAAAAATAAACCAAGTTTTTTGCTTAGAAGATAAGTTACCATAGGGATAACAGCGTAATTTTT
TTTGAGAGTTCGTATTGAAAAAAGAGATTGCGACCTCGATGTTGGATTAAAATTAACCTTA
>BLA28816s
CCAAAAACATGTCTCTTTGAGTTTTTTTAATAAAGAGTTGGGCCTGCTCGGTGATTAATATTTAACAGCTGCGGTATTATAACTGTACTAAGGTAGCATAATA
ATTTGCTCTATAAATTGGGGCTAGAATGAATGGTTTGACGAAAATTTAACTGTCTCTATTTTATTTATTAGAAATTAATTTTTATAGTGAGAAAGCTTAAATT
GTTTAAAGGGACGAAAAGACCCTATTGAGCTTATATTATTTATTAAATTATAGAGTTATAATTTATTATAAATTAATTTTGGTTGGGGTGATCAAGGAATAAG
TTTAATGATTATATAACTTCCTTAACTGAACTATTTTTTGGAAAAATAAACCAAGTTTTTTGCTTAGAAGATAAGTTACCATAGGGATAACAGCGTAATTTTT
TTTGAGAGTTCGTATTGAAAAAAGAGATTGCGACCTCGATGTTGGATTAAAATTAACCTTA
>BLA28916s
CCAAAAACATGTCTCTTTGAGTTTTTTTAATAAAGAGTTGGGCCTGCTCGGTGATTAATATTTAACAGCTGCGGTATTATAACTGTACTAAGGTAGCATAATA
ATTTGCTCTATAAATTGGGGCTAGAATGAATGGTTTGACGAAAATTTAACTGTCTCTATTTTATTTATTAGAAATTAATTTTTATAGTGAGAAAGCTTAAATT
GTTTAAAGGGACGAAAAGACCCTATTGAGCTTATATTATTTATTAAATTATAGAGTTATAATTTATTATAAATTAATTTTGGTTGGGGTGATCAAGGAATAAG
TTTAATGATTATATAACTTCCTTAACTGAACTATTTTTTGGAAAAATAAACCAAGTTTTTTGCTTAGAAGATAAGTTACCATAGGGATAACAGCGTAATTTTT
TTTGAGAGTTCGTATTGAAAAAAGAGATTGCGACCTCGATGTTGGATTAAAATTAACCTTA
>BLA28416s
CCAAAAACATGTCTCTTTGAGTTTTTTTAATAAAGAGTTGGGCCTGCTCGGTGATTAATATTTAACAGCTGCGGTATTATAACTGTACTAAGGTAGCATAATA
ATTTGCTCTATAAATTGGGGCTAGAATGAATGGTTTGACGAAAATTTAACTGTCTCTATTTTATTTATTAGAAATTAATTTTTATAGTGAGAAAGCTTAAATT
GTTTAAAGGGACGAAAAGACCCTATTGAGCTTATATTATTTAGTAAATTATAGAGTTATAATTTATTGTAAATTAATTTTGGTTGGGGTGATCAAGGAATAAG
TTTAATGATTATATAACTTCCTTAACTGAACTATTTTTTGGAAAAATAAACCAAGTTTTTTGCTTAGAAGATAAGTTACCATAGGGATAACAGCGTAATTTTT
TTTGAGAGTTCGTATTGAAAAAAGAGATTGCGACCTCGATGTTGGATTAAAATTAACCTTA
>BLA29016s
CCAAAAACATGTCTCTTTGAGTTTTTTTAATAAAGAGTTGGGCCTGCTCGGTGATTAATATTTAACAGCTGCGGTATTATAACTGTACTAAGGTAGCATAATA
ATTTGCTCTATAAATTGGGGCTAGAATGAATGGTTTGACGAAAATTTAACTGTCTCTATTTTATTTATTAGAAATTAATTTTTATAGTGAGAAAGCTTAAATT
GTTTAAAGGGACGAAAAGACCCTATTGAGCTTATATTATTTATTAAATTATAGAGTTATAATTTATTATAAATTAATTTTGGTTGGGGTGATCAAGGAATAAG
TTTAATGATTATATAACTTCCTTAACTGAACTATTTTTTGGAAAAATAAACCAAGTTTTTTGCTTAGAAGATAAGTTACCATAGGGATAACAGCGTAATTTTT
TTTGAGAGTTCGTATTGAAAAAAGAGATTGCGACCTCGATGTTGGATTAAAATTAACCTTA
>ISLG48316s
CCAAAAACATGTCTCTTTGAGTTTTTTTAATAAAGAGTTGGGCCTGCTCGGTGATTAATATTTAACAGCTGCGGTATTATAACTGTACTAAGGTAGCATAATA
ATTTGCTCTATAAATTGGGGCTAGAATGAATGGTTTGACGAAAATTTAACTGTCTCTATTTTATTTATTAGAAATTAATTTTTATAGTGAGAAAGCTTAAATT
GTTTAAAGGGACGAAAAGACCCTATTGAGCTTATATTATTTATTAAATTATAGAGTTATAATTTATTATAAATTAATTTTGGTTGGGGTGATCAAGGAATAAG
TTTAATGATTATATAACTTCCTTAACTGAACTATTTTTTGGAAAAATAAACCAAGTTTTTTGCTTAGAAGATAAGTTACCATAGGGATAACAGCGTAATTTTT
TTTGAGAGTTCGTATTGAAAAAAGAGATTGCGACCTCGATGTTGGATTAAAATTAACCTTA
>ISLG48416s
CCAAAAACATGTCTCTTTGAGTTTTTTTAATAAAGAGTTGGGCCTGCTCGGTGATTAATATTTAACAGCTGCGGTATTATAACTGTACTAAGGTAGCATAATA
ATTTGCTCTATAAATTGGGGCTAGAATGAATGGTTTGACGAAAATTTAACTGTCTCTATTTTATTTATTAGAAATTAATTTTTATAGTGAGAAAGCTTAAATT
GTTTAAAGGGACGAAAAGACCCTATTGAGCTTATATTATTTATTAAATTATAGAGTTATAATTTATTATAAATTAATTTTGGTTGGGGTGATCAAGGAATAAG
TTTAATGATTATATAACTTCCTTAACTGAACTATTTTTTGGAAAAATAAACCAAGTTTTTTGCTTAGAAGATAAGTTACCATAGGGATAACAGCGTAATTTTT
TTTGAGAGTTCGTATTGAAAAAAGAGATTGCGACCTCGATGTTGGATTAAAATTAACCTTA
>ISLG48516s
CCAAAAACATGTCTCTTTGAGTTTTTTTAATAAAGAGTTGGGCCTGCTCGGTGATTAATATTTAACAGCTGCGGTATTATAACTGTACTAAGGTAGCATAATA
ATTTGCTCTATAAATTGGGGCTAGAATGAATGGTTTGACGAAAATTTAACTGTCTCTATTTTATTTATTAGAAATTAATTTTTATAGTGAGAAAGCTTAAATT
GTTTAAAGGGACGAAAAGACCCTATTGAGCTTATATTATTTATTAAATTATAGAGTTATAATTTATTGTAAATTAATTTTGGTTGGGGTGATCAAGGAATAAG
TTTAATGATTATATAACTTCCTTAACTGAACTATTTTTTGGAAAAATAAACCAAGTTTTTTGCTTAGAAGATAAGTTACCATAGGGATAACAGCGTAATTTTT
TTTGAGAGTTCGTATTGAAAAAAGAGATTGCGACCTCGATGTTGGATTAAAATTAACCTTA
>ISLG48616s
CCAAAAACATGTCTCTTTGAGTTTTTTTAATAAAGAGTTGGGCCTGCTCGGTGATTAATATTTAACAGCTGCGGTATTATAACTGTACTAAGGTAGCATAATA
ATTTGCTCTATAAATTGGGGCTAGAATGAATGGTTTGACGAAAATTTAACTGTCTCTATTTTATTTATTAGAAATTAATTTTTATAGTGAGAAAGCTTAAATT
GTTTAAAGGGACGAAAAGACCCTATTGAGCTTATATTATTTATTAAATTATAGAGTTATAATTTATTATAAATTAATTTTGGTTGGGGTGATCAAGGAATAAG
TTTAATGATTATATAACTTCCTTAACTGAACTATTTTTTGGAAAAATAAACCAAGTTTTTTGCTTAGAAGATAAGTTACCATAGGGATAACAGCGTAATTTTT
TTTGAGAGTTCGTATTGAAAAAAGAGATTGCGACCTCGATGTTGGATTAAAATTAACCTTA
>ISLG48716s
CCAAAAACATGTCTCTTTGAGTTTTTTTAATAAAGAGTTGGGCCTGCTCGGTGATTAATATTTAACAGCTGCGGTATTATAACTGTACTAAGGTAGCATAATA
ATTTGCTCTATAAATTGGGGCTAGAATGAATGGTTTGACGAAAATTTAACTGTCTCTATTTTATTTATTAGAAATTAATTTTTATAGTGAGAAAGCTTAAATT
GTTTAAAGGGACGAAAAGACCCTATTGAGCTTATATTATTTATTAAATTATAGAGTTATAATTTATTATAAATTAATTTTGGTTGGGGTGATCAAGGAATAAG
TTTAATGATTATATAACTTCCTTAACTGAACTATTTTTTGGAAAAATAAACCAAGTTTTTTGCTTAGAAGATAAGTTACCATAGGGATAACAGCGTAATTTTT
TTTGAGAGTTCGTATTGAAAAAAGAGATTGCGACCTCGATGTTGGATTAAAATTAACCTTA
>ISLG48816s
CCAAAAACATGTCTCTTTGAGTTTTTTTAATAAAGAGTTGGGCCTGCTCGGTGATTAATATTTAACAGCTGCGGTATTATAACTGTACTAAGGTAGCATAATA
ATTTGCTCTATAAATTGGGGCTAGAATGAATGGTTTGACGAAAATTTAACTGTCTCTATTTTATTTATTAGAAATTAATTTTTATAGTGAGAAAGCTTAAATT
GTTTAAAGGGACGAAAAGACCCTATTGAGCTTATATTATTTATTAAATTATAGAGTTATAATTTATTATAAATTAATTTTGGTTGGGGTGATCAAGGAATAAG
TTTAATGATTATATAACTTCCTTAACTGAACTATTTTTTGGAAAAATAAACCAAGTTTTTTGCTTAGAAGATAAGTTACCATAGGGATAACAGCGTAATTTTT
TTTGAGAGTTCGTATTGAAAAAAGAGATTGCGACCTCGATGTTGGATTAAAATTAACCTTA
>ISLG48916s
CCAAAAACATGTCTCTTTGAGTTTTTTTAATAAAGAGTTGGGCCTGCTCGGTGATTAATATTTAACAGCTGCGGTATTATAACTGTACTAAGGTAGCATAATA
ATTTGCTCTATAAATTGGGGCTAGAATGAATGGTTTGACGAAAATTTAACTGTCTCTATTTTATTTATTAGAAATTAATTTTTATAGTGAGAAAGCTTAAATT
GTTTAAAGGGACGAAAAGACCCTATTGAGCTTATATTATTTATTAAATTATAGAGTTATAATTTATTATAAATTAATTTTGGTTGGGGTGATCAAGGAATAAG
TTTAATGATTATATAACTTCCTTAACTGAACTATTTTTTGGAAAAATAAACCAAGTTTTTTGCTTAGAAGATAAGTTACCATAGGGATAACAGCGTAATTTTT
TTTGAGAGTTCGTATTGAAAAAAGAGATTGCGACCTCGATGTTGGATTAAAATTAACCTTA
>ISLG49016s
CCAAAAACATGTCTCTTTGAGTTTTTTTAATAAAGAGTTGGGCCTGCTCGGTGATTAATATTTAACAGCTGCGGTATTATAACTGTACTAAGGTAGCATAATA
ATTTGCTCTATAAATTGGGGCTAGAATGAATGGTTTGACGAAAATTTAACTGTCTCTATTTTATTTATTAGAAATTAATTTTTATAGTGAGAAAGCTTAAATT

GTTTAAAGGGACGAAAAGACCCTATTGAGCTTATATTATTTATTAAATTATAGAGTTATAATTTATTATATAAATTAATTTTGGTTGGGGTGATCAAGGAATAAG
TTTAATGATTATATAACTTCCTTAACTGAACTATTTTTTGGAAAAATAAACCAAGTTTTTTGCTTAGAAGATAAGTTACCATAGGGATAACAGCGTAATTTTT
TTTGAGAGTTCGTATTGAAAAAAGAGATTGCGACCTCGATGTTGGATTAAAATTAACCTTA
>MAL11916s
CCAAAAACATGTCTCTTTGAGTTTTTTTAATAAAGAGTTGGGCCTGCTCGGTGATTAATATTTAACAGCTGCGGTATTATAACTGTACTAAGGTAGCATAATA
ATTTGCTCTATAAATTGGGGCTAGAATGAATGGTTTGACGAAAATTTAACTGTCTCTATTTTATTTATTAGAAATTAATTTTTATAGTGAGAAAGCTTAAATT
GTTTAAAGGGACGAAAAGACCCTATTGAGCTTATATTATTTATTAAATTATAGAGTTATAATTTATTGTAAATTAATTTTGGTTGGGGTGATCAAGGAATAAG
TTTAATGATTATATAACTTCCTTAACTGAACTATTTTTTGGAAAAATAAACCAAGTTTTTTGCTTAGAAGATAAGTTACCATAGGGATAACAGCGTAATTTTT
TTTGAGAGTTCGTATTGAAAAAAGAGATTGCGACCTCGATGTTGGATTAAAATTAACCTTA
>MAL12016s
CCAAAAACATGTCTCTTTGAGTTTTTTTAATAAAGAGTTGGGCCTGCTCGGTGATTAATATTTAACAGCTGCGGTATTATAACTGTACTAAGGTAGCATAATA
ATTTGCTCTATAAATTGGGGCTAGAATGAATGGTTTGACGAAAATTTAACTGTCTCTATTTTATTTATTAGAAATTAATTTTTATAGTGAGAAAGCTTAAATT
GTTTAAAGGGACGAAAAGACCCTATTGAGCTTATATTATTTATTAAATTATAGAGTTATAATTTATTGTAAATTAATTTTGGTTGGGGTGATCAAGGAATAAG
TTTAATGATTATATAACTTCCTTAACTGAACTATTTTTTGGAAAAATAAACCAAGTTTTTTGCTTAGAAGATAAGTTACCATAGGGATAACAGCGTAATTTTT
TTTGAGAGTTCGTATTGAAAAAAGAGATTGCGACCTCGATGTTGGATTAAAATTAACCTTA
>MAL12116s
CCAAAAACATGTCTCTTTGAGTTTTTTTAATAAAGAGTTGGGCCTGCTCGGTGATTAATATTTAACAGCTGCGGTATTATAACTGTACTAAGGTAGCATAATA
ATTTGCTCTATAAATTGGGGCTAGAATGAATGGTTTGACGAAAATTTAACTGTCTCTATTTTATTTATTAGAAATTAATTTTTATAGTGAGAAAGCTTAAATT
GTTTAAAGGGACGAAAAGACCCTATTGAGCTTATATTATTTATTAAATTATAGAGTTATAATTTATTGTAAATTAATTTTGGTTGGGGTGATCAAGGAATAAG
TTTAATGATTATATAACTTCCTTAACTGAACTATTTTTTGGAAAAATAAACCAAGTTTTTTGCTTAGAAGATAAGTTACCATAGGGATAACAGCGTAATTTTT
TTTGAGAGTTCGTATTGAAAAAAGAGATTGCGACCTCGATGTTGGATTAAAATTAACCTTA
>MAL12216s
CCAAAAACATGTCTCTTTGAGTTTTTTTAATAAAGAGTTGGGCCTGCTCGGTGATTAATATTTAACAGCTGCGGTATTATAACTGTACTAAGGTAGCATAATA
ATTTGCTCTATAAATTGGGGCTAGAATGAATGGTTTGACGAAAATTTAACTGTCTCTATTTTATTTATTAGAAATTAATTTTTATAGTGAGAAAGCTTAAATT
GTTTAAAGGGACGAAAAGACCCTATTGAGCTTATATTATTTATTAAATTATAGAGTTATAATTTATTGTAAATTAATTTTGGTTGGGGTGATCAAGGAATAAG
TTTAATGATTATATAACTTCCTTAACTGAACTATTTTTTGGAAAAATAAACCAAGTTTTTTGCTTAGAAGATAAGTTACCATAGGGATAACAGCGTAATTTTT
TTTGAGAGTTCGTATTGAAAAAAGAGATTGCGACCTCGATGTTGGATTAAAATTAACCTTA
>MAL12316s
CCAAAAACATGTCTCTTTGAGTTTTTTTAATAAAGAGTTGGGCCTGCTCGGTGATTAATATTTAACAGCTGCGGTATTATAACTGTACTAAGGTAGCATAATA
ATTTGCTCTATAAATTGGGGCTAGAATGAATGGTTTGACGAAAATTTAACTGTCTCTATTTTATTTATTAGAAATTAATTTTTATAGTGAGAAAGCTTAAATT
GTTTAAAGGGACGAAAAGACCCTATTGAGCTTATATTATTTATTAAATTATAGAGTTATAATTTATTGTAAATTAATTTTGGTTGGGGTGATCAAGGAATAAG
TTTAATGATTATATAACTTCCTTAACTGAACTATTTTTTGGAAAAATAAACCAAGTTTTTTGCTTAGAAGATAAGTTACCATAGGGATAACAGCGTAATTTTT
TTTGAGAGTTCGTATTGAAAAAAGAGATTGCGACCTCGATGTTGGATTAAAATTAACCTTA
>MAL12416s
CCAAAAACATGTCTCTTTGAGTTTTTTTAATAAAGAGTTGGGCCTGCTCGGTGATTAATATTTAACAGCTGCGGTATTATAACTGTACTAAGGTAGCATAATA
ATTTGCTCTATAAATTGGGGCTAGAATGAATGGTTTGACGAAAATTTAACTGTCTCTATTTTATTTATTAGAAATTAATTTTTATAGTGAGAAAGCTTAAATT
GTTTAAAGGGACGAAAAGACCCTATTGAGCTTATATTATTTATTAAATTATAGAGTTATAATTTATTGTAAATTAATTTTGGTTGGGGTGATCAAGGAATAAG
TTTAATGATTATATAACTTCCTTAACTGAACTATTTTTTGGAAAAATAAACCAAGTTTTTTGCTTAGAAGATAAGTTACCATAGGGATAACAGCGTAATTTTT
TTTGAGAGTTCGTATTGAAAAAAGAGATTGCGACCTCGATGTTGGATTAAAATTAACCTTA
>MAL12516s
CCAAAAACATGTCTCTTTGAGTTTTTTTAATAAAGAGTTGGGCCTGCTCGGTGATTAATATTTAACAGCTGCGGTATTATAACTGTACTAAGGTAGCATAATA
ATTTGCTCTATAAATTGGGGCTAGAATGAATGGTTTGACGAAAATTTAACTGTCTCTATTTTATTTATTAGAAATTAATTTTTATAGTGAGAAAGCTTAAATT
GTTTAAAGGGACGAAAAGACCCTATTGAGCTTATATTATTTATTAAATTATAGAGTTATAATTTATTGTAAATTAATTTTGGTTGGGGTGATCAAGGAATAAG
TTTAATGATTATATAACTTCCTTAACTGAACTATTTTTTGGAAAAATAAACCAAGTTTTTTGCTTAGAAGATAAGTTACCATAGGGATAACAGCGTAATTTTT
TTTGAGAGTTCGTATTGAAAAAAGAGATTGCGACCTCGATGTTGGATTAAAATTAACCTTA
>MAL12616s
CCAAAAACATGTCTCTTTGAGTTTTTTTAATAAAGAGTTGGGCCTGCTCGGTGATTAATATTTAACAGCTGCGGTATTATAACTGTACTAAGGTAGCATAATA
ATTTGCTCTATAAATTGGGGCTAGAATGAATGGTTTGACGAAAATTTAACTGTCTCTATTTTATTTATTAGAAATTAATTTTTATAGTGAGAAAGCTTAAATT
GTTTAAAGGGACGAAAAGACCCTATTGAGCTTATATTATTTATTAAATTATAGAGTTATAATTTATTGTAAATTAATTTTGGTTGGGGTGATCAAGGAATAAG
TTTAATGATTATATAACTTCCTTAACTGAACTATTTTTTGGAAAAATAAACCAAGTTTTTTGCTTAGAAGATAAGTTACCATAGGGATAACAGCGTAATTTTT
TTTGAGAGTTCGTATTGAAAAAAGAGATTGCGACCTCGATGTTGGATTAAAATTAACCTTA
>PLO35616s
CCAAAAACATGTCTCTTTGAGTTTTTTTAATAAAGAGTTGGGCCTGCTCGGTGATTAATATTTAACAGCTGCGGTATTATAACTGTACTAAGGTAGCATAATA
ATTTGCTCTATAAATTGGGGCTAGAATGAATGGTTTGACGAAAATTTAACTGTCTCTATTTTATTTATTAGAAATTAATTTTTATAGTGAGAAAGCTTAAATT
GTTTAAAGGGACGAAAAGACCCTATTGAGCTTATATTATTTATTAAATTATAGAGTTATAATTTATTATATAAATTAATTTTGGTTGGGGTGATCAAGGAATAAG
TTTAATGATTATATAACTTCCTTAACTGAACTATTTTTTGGAAAAATAAACCAAGTTTTTTGCTTAGAAGATAAGTTACCATAGGGATAACAGCGTAATTTTT
TTTGAGAGTTCGTATTGAAAAAAGAGATTGCGACCTCGATGTTGGATTAAAATTAACCTTA
>PLO35716s
CCAAAAACATGTCTCTTTGAGTTTTTTTAATAAAGAGTTGGGCCTGCTCGGTGATTAATATTTAACAGCTGCGGTATTATAACTGTACTAAGGTAGCATAATA
ATTTGCTCTATAAATTGGGGCTAGAATGAATGGTTTGACGAAAATTTAACTGTCTCTATTTTATTTATTAGAAATTAATTTTTATAGTGAGAAAGCTTAAATT
GTTTAAAGGGACGAAAAGACCCTATTGAGCTTATATTATTTATTAAATTATAGAGTTATAATTTATTATATAAATTAATTTTGGTTGGGGTGATCAAGGAATAAG
TTTAATGATTATATAACTTCCTTAACTGAACTATTTTTTGGAAAAATAAACCAAGTTTTTTGCTTAGAAGATAAGTTACCATAGGGATAACAGCGTAATTTTT
TTTGAGAGTTCGTATTGAAAAAAGAGATTGCGACCTCGATGTTGGATTAAAATTAACCTTA
>PLO35816s
CCAAAAACATGTCTCTTTGAGTTTTTTTAATAAAGAGTTGGGCCTGCTCGGTGATTAATATTTAACAGCTGCGGTATTATAACTGTACTAAGGTAGCATAATA
ATTTGCTCTATAAATTGGGGCTAGAATGAATGGTTTGACGAAAATTTAACTGTCTCTATTTTATTTATTAGAAATTAATTTTTATAGTGAGAAAGCTTAAATT
GTTTAAAGGGACGAAAAGACCCTATTGAGCTTATATTATTTATTAAATTATAGAGTTATAATTTATTGTAAATTAATTTTGGTTGGGGTGATCAAGGAATAAG
TTTAATGATTATATAACTTCCTTAACTGAACTATTTTTTGGAAAAATAAACCAAGTTTTTTGCTTAGAAGATAAGTTACCATAGGGATAACAGCGTAATTTTT
TTTGAGAGTTCGTATTGAAAAAAGAGATTGCGACCTCGATGTTGGATTAAAATTAACCTTA
>PLO35916s
CCAAAAACATGTCTCTTTGAGTTTTTTTAATAAAGAGTTGGGCCTGCTCGGTGATTAATATTTAACAGCTGCGGTATTATAACTGTACTAAGGTAGCATAATA
ATTTGCTCTATAAATTGGGGCTAGAATGAATGGTTTGACGAAAATTTAACTGTCTCTATTTTATTTATTAGAAATTAATTTTTATAGTGAGAAAGCTTAAATT
GTTTAAAGGGACGAAAAGACCCTATTGAGCTTATATTATTTATTAAATTATAGAGTTATAATTTATTATATAAATTAATTTTGGTTGGGGTGATCAAGGAATAAG
TTTAATGATTATATAACTTCCTTAACTGAACTATTTTTTGGAAAAATAAACCAAGTTTTTTGCTTAGAAGATAAGTTACCATAGGGATAACAGCGTAATTTTT
TTTGAGAGTTCGTATTGAAAAAAGAGATTGCGACCTCGATGTTGGATTAAAATTAACCTTA
>PLO36016s
CCAAAAACATGTCTCTTTGAGTTTTTTTAATAAAGAGTTGGGCCTGCTCGGTGATTAATATTTAACAGCTGCGGTATTATAACTGTACTAAGGTAGCATAATA
ATTTGCTCTATAAATTGGGGCTAGAATGAATGGTTTGACGAAAATTTAACTGTCTCTATTTTATTTATTAGAAATTAATTTTTATAGTGAGAAAGCTTAAATT
GTTTAAAGGGACGAAAAGACCCTATTGAGCTTATATTATTTATTAAATTATAGAGTTATAATTTATTATATAAATTAATTTTGGTTGGGGTGATCAAGGAATAAG

TTTAATGATTATATAACTTCCTTAACTGAACTATTTTTTGGAAAAATAAACCAAGTTTTTTGCTTAGAAGATAAGTTACCATAGGGATAACAGCGTAATTTTT
TTTGAGAGTTCGTATTGAAAAAAGAGATTGCGACCTCGATGTTGGATTAAAATTAACCTTA
>PLO36116s
CCAAAAACATGTCTCTTTGAGTTTTTTTAATAAAGAGTTGGGCCTGCTCGGTGATTAATATTTAACAGCTGCGGTATTATAACTGTACTAAGGTAGCATAATA
ATTTGCTCTATAAATTGGGGCTAGAATGAATGGTTTGACGAAAATTTAACTGTCTCTATTTTATTTATTAGAAATTAATTTTTATAGTGAGAAAGCTTAAATT
GTTTAAAGGGACGAAAAGACCCTATTGAGCTTATATTATTTATTAAATTATAGAGTTATAATTTATTATAAATTAATTTTGGTTGGGGTGATCAAGGAATAAG
TTTAATGATTATATAACTTCCTTAACTGAACTATTTTTTGGAAAAATAAACCAAGTTTTTTGCTTAGAAGATAAGTTACCATAGGGATAACAGCGTAATTTTT
TTTGAGAGTTCGTATTGAAAAAAGAGATTGCGACCTCGATGTTGGATTAAAATTAACCTTA
>PLO36216s
CCAAAAACATGTCTCTTTGAGTTTTTTTAATAAAGAGTTGGGCCTGCTCGGTGATTAATATTTAACAGCTGCGGTATTATAACTGTACTAAGGTAGCATAATA
ATTTGCTCTATAAATTGGGGCTAGAATGAATGGTTTGACGAAAATTTAACTGTCTCTATTTTATTTATTAGAAATTAATTTTTATAGTGAGAAAGCTTAAATT
GTTTAAAGGGACGAAAAGACCCTATTGAGCTTATATTATTTATTAAATTATAGAGTTATAATTTATTATAAATTAATTTTGGTTGGGGTGATCAAGGAATAAG
TTTAATGATTATATAACTTCCTTAACTGAACTATTTTTTGGAAAAATAAACCAAGTTTTTTGCTTAGAAGATAAGTTACCATAGGGATAACAGCGTAATTTTT
TTTGAGAGTTCGTATTGAAAAAAGAGATTGCGACCTCGATGTTGGATTAAAATTAACCTTA
>PLO36316s
CCAAAAACATGTCTCTTTGAGTTTTTTTAATAAAGAGTTGGGCCTGCTCGGTGATTAATATTTAACAGCTGCGGTATTATAACTGTACTAAGGTAGCATAATA
ATTTGCTCTATAAATTGGGGCTAGAATGAATGGTTTGACGAAAATTTAACTGTCTCTATTTTATTTATTAGAAATTAATTTTTATAGTGAGAAAGCTTAAATT
GTTTAAAGGGACGAAAAGACCCTATTGAGCTTATATTATTTATTAAATTATAGAGTTATAATTTATTATAAATTAATTTTGGTTGGGGTGATCAAGGAATAAG
TTTAATGATTATATAACTTCCTTAACTGAACTATTTTTTGGAAAAATAAACCAAGTTTTTTGCTTAGAAGATAAGTTACCATAGGGATAACAGCGTAATTTTT
TTTGAGAGTTCGTATTGAAAAAAGAGATTGCGACCTCGATGTTGGATTAAAATTAACCTTA
>PPE29716s
CCAAAAACATGTCTCTTTGAGTTTTTTTAATAAAGAGTTGGGCCTGCTCGGTGATTAATATTTAACAGCTGCGGTATTATAACTGTACTAAGGTAGCATAATA
ATTTGCTCTATAAATTGGGGCTAGAATGAATGGTTTGACGAAAATTTAACTGTCTCTATTTTATTTATTAGAAATTAATTTTTATAGTGAGAAAGCTTAAATT
GTTTAAAGGGACGAAAAGACCCTATTGAGCTTATATTATTTATTAAATTATAGAGTTATAATTTATTATAAATTAATTTTGGTTGGGGTGATCAAGGAATAAG
TTTAATGATTATATAACTTCCTTAACTGAACTATTTTTTGGAAAAATAAACCAAGTTTTTTGCTTAGAAGATAAGTTACCATAGGGATAACAGCGTAATTTTT
TTTGAGAGTTCGTATTGAAAAAAGAGATTGCGACCTCGATGTTGGATTAAAATTAACCTTA
>PPE29816s
CCAAAAACATGTCTCTTTGAGTTTTTTTAATAAAGAGTTGGGCCTGCTCGGTGATTAATATTTAACAGCTGCGGTATTATAACTGTACTAAGGTAGCATAATA
ATTTGCTCTATAAATTGGGGCTAGAATGAATGGTTTGACGAAAATTTAACTGTCTCTATTTTATTTATTAGAAATTAATTTTTATAGTGAGAAAGCTTAAATT
GTTTAAAGGGACGAAAAGACCCTATTGAGCTTATATTATTTATTAAATTATAGAGTTATAATTTATTATAAATTAATTTTGGTTGGGGTGATCAAGGAATAAG
TTTAATGATTATATAACTTCCTTAACTGAACTATTTTTTGGAAAAATAAACCAAGTTTTTTGCTTAGAAGATAAGTTACCATAGGGATAACAGCGTAATTTTT
TTTGAGAGTTCGTATTGAAAAAAGAGATTGCGACCTCGATGTTGGATTAAAATTAACCTTA
>PPE29916s
CCAAAAACATGTCTCTTTGAGTTTTTTTAATAAAGAGTTGGGCCTGCTCGGTGATTAATATTTAACAGCTGCGGTATTATAACTGTACTAAGGTAGCATAATA
ATTTGCTCTATAAATTGGGGCTAGAATGAATGGTTTGACGAAAATTTAACTGTCTCTATTTTATTTATTAGAAATTAATTTTTATAGTGAGAAAGCTTAAATT
GTTTAAAGGGACGAAAAGACCCTATTGAGCTTATATTATTTATTAAATTATAGAGTTATAATTTATTATAAATTAATTTTGGTTGGGGTGATCAAGGAATAAG
TTTAATGATTATATAACTTCCTTAACTGAACTATTTTTTGGAAAAATAAACCAAGTTTTTTGCTTAGAAGATAAGTTACCATAGGGATAACAGCGTAATTTTT
TTTGAGAGTTCGTATTGAAAAAAGAGATTGCGACCTCGATGTTGGATTAAAATTAACCTTA
>PPE30016s
CCAAAAACATGTCTCTTTGAGTTTTTTTAATAAAGAGTTGGGCCTGCTCGGTGATTAATATTTAACAGCTGCGGTATTATAACTGTACTAAGGTAGCATAATA
ATTTGCTCTATAAATTGGGGCTAGAATGAATGGTTTGACGAAAATTTAACTGTCTCTATTTTATTTATTAGAAATTAATTTTTATAGTGAGAAAGCTTAAATT
GTTTAAAGGGACGAAAAGACCCTATTGAGCTTATATTATTTATTAAATTATAGAGTTATAATTTATTATAAATTAATTTTGGTTGGGGTGATCAAGGAATAAG
TTTAATGATTATATAACTTCCTTAACTGAACTATTTTTTGGAAAAATAAACCAAGTTTTTTGCTTAGAAGATAAGTTACCATAGGGATAACAGCGTAATTTTT
TTTGAGAGTTCGTATTGAAAAAAGAGATTGCGACCTCGATGTTGGATTAAAATTAACCTTA
>PPE30116s
CCAAAAACATGTCTCTTTGAGTTTTTTTAATAAAGAGTTGGGCCTGCTCGGTGATTAATATTTAACAGCTGCGGTATTATAACTGTACTAAGGTAGCATAATA
ATTTGCTCTATAAATTGGGGCTAGAATGAATGGTTTGACGAAAATTTAACTGTCTCTATTTTATTTATTAGAAATTAATTTTTATAGTGAGAAAGCTTAAATT
GTTTAAAGGGACGAAAAGACCCTATTGAGCTTATATTATTTATTAAATTATAGAGTTATAATTTATTGTAAATTAATTTTGGTTGGGGTGATCAAGGAATAAG
TTTAATGATTATATAACTTCCTTAACTGAACTATTTTTTGGAAAAATAAACCAAGTTTTTTGCTTAGAAGATAAGTTACCATAGGGATAACAGCGTAATTTTT
TTTGAGAGTTCGTATTGAAAAAAGAGATTGCGACCTCGATGTTGGATTAAAATTAACCTTA
>PPE30216s
CCAAAAACATGTCTCTTTGAGTTTTTTTAATAAAGAGTTGGGCCTGCTCGGTGATTAATATTTAACAGCTGCGGTATTATAACTGTACTAAGGTAGCATAATA
ATTTGCTCTATAAATTGGGGCTAGAATGAATGGTTTGACGAAAATTTAACTGTCTCTATTTTATTTATTAGAAATTAATTTTTATAGTGAGAAAGCTTAAATT
GTTTAAAGGGACGAAAAGACCCTATTGAGCTTATATTATTTATTAAATTATAGAGTTATAATTTATTGTAAATTAATTTTGGTTGGGGTGATCAAGGAATAAG
TTTAATGATTATATAACTTCCTTAACTGAACTATTTTTTGGAAAAATAAACCAAGTTTTTTGCTTAGAAGATAAGTTACCATAGGGATAACAGCGTAATTTTT
TTTGAGAGTTCGTATTGAAAAAAGAGATTGCGACCTCGATGTTGGATTAAAATTAACCTTA
>PPE30316s
CCAAAAACATGTCTCTTTGAGTTTTTTTAATAAAGAGTTGGGCCTGCTCGGTGATTAATATTTAACAGCTGCGGTATTATAACTGTACTAAGGTAGCATAATA
ATTTGCTCTATAAATTGGGGCTAGAATGAATGGTTTGACGAAAATTTAACTGTCTCTATTTTATTTATTAGAAATTAATTTTTATAGTGAGAAAGCTTAAATT
GTTTAAAGGGACGAAAAGACCCTATTGAGCTTATATTATTTATTAAATTATAGAGTTATAATTTATTATAAATTAATTTTGGTTGGGGTGATCAAGGAATAAG
TTTAATGATTATATAACTTCCTTAACTGAACTATTTTTTGGAAAAATAAACCAAGTTTTTTGCTTAGAAGATAAGTTACCATAGGGATAACAGCGTAATTTTT
TTTGAGAGTTCGTATTGAAAAAAGAGATTGCGACCTCGATGTTGGATTAAAATTAACCTTA
>PPE30416s
CCAAAAACATGTCTCTTTGAGTTTTTTTAATAAAGAGTTGGGCCTGCTCGGTGATTAATATTTAACAGCTGCGGTATTATAACTGTACTAAGGTAGCATAATA
ATTTGCTCTATAAATTGGGGCTAGAATGAATGGTTTGACGAAAATTTAACTGTCTCTATTTTATTTATTAGAAATTAATTTTTATAGTGAGAAAGCTTAAATT
GTTTAAAGGGACGAAAAGACCCTATTGAGCTTATATTATTTATTAAATTATAGAGTTATAATTTATTATAAATTAATTTTGGTTGGGGTGATCAAGGAATAAG
TTTAATGATTATATAACTTCCTTAACTGAACTATTTTTTGGAAAAATAAACCAAGTTTTTTGCTTAGAAGATAAGTTACCATAGGGATAACAGCGTAATTTTT
TTTGAGAGTTCGTATTGAAAAAAGAGATTGCGACCTCGATGTTGGATTAAAATTAACCTTA
>PRE26816s
CCAAAAACATGTCTCTTTGAGTTTTTTTAATAAAGAGTTGGGCCTGCTCGGTGATTAATATTTAACAGCTGCGGTATTATAACTGTACTAAGGTAGCATAATA
ATTTGCTCTATAAATTGGGGCTAGAATGAATGGTTTGACGAAAATTTAACTGTCTCTATTTTATTTATTAGAAATTAATTTTTATAGTGAGAAAGCTTAAATT
GTTTAAAGGGACGAAAAGACCCTATTGAGCTTATATTATTTATTAAATTATAGAGTTATAATTTATTATAAATTAATTTTGGTTGGGGTGATCAAGGAATAAG
TTTAATGATTATATAACTTCCTTAACTGAACTATTTTTTGGAAAAATAAACCAAGTTTTTTGCTTAGAAGATAAGTTACCATAGGGATAACAGCGTAATTTTT
TTTGAGAGTTCGTATTGAAAAAAGAGATTGCGACCTCGATGTTGGATTAAAATTAACCTTA
>PLI44116s
CCAAAAACATGTCTCTTTGAGTTTTTTTAATAAAGAGTTGGGCCTGCTCGGTGATTAATATTTAACAGCTGCGGTATTATAACTGTACTAAGGTAGCATAATA
ATTTGCTCTATAAATTGGGGCTAGAATGAATGGTTTGACGAAAATTTAACTGTCTCTATTTTATTTATTAGAAATTAATTTTTATAGTGAGAAAGCTTAAATT
GTTTAAAGGGACGAAAAGACCCTATTGAGCTTATATTATTTATTAAATTATAGAGTTATAATTTATTGTAAATTAATTTTGGTTGGGGTGATCAAGGAATAAG
TTTAATGATTATATAACTTCCTTAACTGAACTATTTTTTGGAAAAATAAACCAAGTTTTTTGCTTAGAAGATAAGTTACCATAGGGATAACAGCGTAATTTTT

TTTGAGAGTTCGTATTGAAAAAAGAGATTGCGACCTCGATGTTGGATTAAAATTAACCTTA
>PLI44316s
CCAAAAACATGTCTCTTTGAGATTTTTTAATAAAGAGTTGGGCCTGCTCGGTGATTAATATTTAACAGCTGCGGTATTATAACTGTACTAAGGTAGCATAATA
ATTTGCTCTATAAATTGGGGCTAGAATGAATGGTTTGACGAAAATTTGACTGTCTCTATCTTATTTATTAGAAATTAATTTTTGTAGTGAGAAAGCTTAAATT
ATTTAAAGGGACGAAAAGACCCTATTGAGCTTGTATTATTTAACAAATTATATGATTGTTATTTGTTTGAATTAATTTTGATTGGGGTGATCAAGGAATAAAT
TTTATTTAGTTTAACTTCCTTAATTAATTGTTTTATAGAAGAATAAACCAAAGTTTTTGCTTAGAAGATAAGTTACCATAGGGATAACAGCGTAATTTTTTTT
GAGAGTTCATATTGAAAAAAGAGATTGCGACCTCGATGTTGGATTAAAATTAACCTTA
>PLI44416s
CCAAAAACATGTCTCTTTGAGATTTTTTAATAAAGAGTTGGGCCTGCTCGGTGATTAATATTTAACAGCTGCGGTATTATAACTGTACTAAGGTAGCATAATA
ATTTGCTCTATAAATTGGGGCTAGAATGAATGGTTTGACGAAAATTTGACTGTCTCTATCTTATTTATTAGAAATTAATTTTTGTAGTGAGAAAGCTTAAATT
ATTTAAAGGGACGAAAAGACCCTATTGAGCTTGTATTATTTAACAAATTATATGATTGTTATTTGTTTGAATTAATTTTGATTGGGGTGATCAAGGAATAAAT
TTTATTTAGTTTAACTTCCTTAGTTAATTGTTTTATAGAAGAATAAACCAAAGTTTTTGCTTAGAAGATAAGTTACCATAGGGATAACAGCGTAATTTTTTTT
GAGAGTTCATATTGAAAAAAGAGATTGCGACCTCGATGTTGGATTAAAATTAACCTTA
>PLI44616s
CCAAAAACATGTCTCTTTGAGATTTTTTAATAAAGAGTTGGGCCTGCTCGGTGATTAATATTTAACAGCTGCGGTATTATAACTGTACTAAGGTAGCATAATA
ATTTGCTCTATAAATTGGGGCTAGAATGAATGGTTTGACGAAAATTTGACTGTCTCTATCTTATTTATTAGAAATTAATTTTTGTAGTGAGAAAGCTTAAATT
ATTTAAAGGGACGAAAAGACCCTATTGAGCTTGTATTATTTAACAAATTATATAATTGTTATTTGTTTGAATTAATTTTGATTGGGGTGATCAAGGAATAAAT
TTTATTTAGTTTAACTTCCTTAGTTAATTGTTTTATAGAAGAATAAACCAAAGTTTTTGCTTAGAAGATAAGTTACCATAGGGATAACAGCGTAATTTTTTTT
GAGAGTTCATATTGAAAAAAGAGATTGCGACCTCGATGTTGGATTAAAATTAACCTTA
>PLI44716s
CCAAAAACATGTCTCTTTGAGATTTTTTAATAAAGAGTTGGGCCTGCTCGGTGATTAATATTTAACAGCTGCGGTATTATAACTGTACTAAGGTAGCATAATA
ATTTGCTCTATAAATTGGGGCTAGAATGAATGGTTTGACGAAAATTTGACTGTCTCTATCTTATTTATTAGAAATTAATTTTTGTAGTGAGAAAGCTTAAATT
ATTTAAAGGGACGAAAAGACCCTATTGAGCTTGTATTATTTAATAAATTATATAATTGTTATTTGTTTGAATTAATTTTGATTGGGGTGATCAAGGAATAAAT
TTTATTTAGTTTAACTTCCTTAGTTAGTTGTTTTATAGAAGAATAAACCAAAGTTTTTGCTTAGAAGATAAGTTACCATAGGGATAACAGCGTAATTTTTTTT
GAGAGTTCATATTGAAAAAAGAGATTGCGACCTCGATGTTGGATTAAAATTAACCTTA
>PLI44816s
CCAAAAACATGTCTCTTTGAGATTTTTTAATAAAGAGTTGGGCCTGCTCGGTGATTAATATTTAACAGCTGCGGTATTATAACTGTACTAAGGTAGCATAATA
ATTTGCTCTATAAATTGGGGCTAGAATGAATGGTTTGACGAAAATTTGACTGTCTCTATCTTATTTATTAGAAATTAATTTTTGTAGTGAGAAAGCTTAAATT
ATTTAAAGGGACGAAAAGACCCTATTGAGCTTGTATTATTTAACAAATTATATGATTGTTATTTGTTTGAATTAATTTTGATTGGGGTGATCAAGGAATAAAT
TTTATTTAGTTTAACTTCCTTAATTAATTGTTTTATAGAAGAATAAACCAAAGTTTTTGCTTAGAAGATAAGTTACCATAGGGATAACAGCGTAATTTTTTTT
GAGAGTTCATATTGAAAAAAGAGATTGCGACCTCGATGTTGGATTAAAATTAACCTTA
>PLI44916s
CCAAAAACATGTCTCTTTGAGATTTTTTAATAAAGAGTTGGGCCTGCTCGGTGATTAATATTTAACAGCTGCGGTATTATAACTGTACTAAGGTAGCATAATA
ATTTGCTCTATAAATTGGGGCTAGAATGAATGGTTTGACGAAAATTTGACTGTCTCTATCTTATTTATTAGAAATTAATTTTTGTAGTGAGAAAGCTTAAATT
ATTTAAAGGGACGAAAAGACCCTATTGAGCTTGTATTATTTAACAAATTATATGATTGTTATTTGTTTGAATTAATTTTGATTGGGGTGATCAAGGAATAAAT
TTTATTTAGTTTAACTTCCTTAGTTAATTGTTTTATAGAAGAATAAACCAAAGTTTTTGCTTAGAAGATAAGTTACCATAGGGATAACAGCGTAATTTTTTTT
GAGAGTTCATATTGAAAAAAGAGATTGCGACCTCGATGTTGGATTAAAATTAACCTTA
>PLI45016s
CCAAAAACATGTCTCTTTGAGATTTTTTAATAAAGAGTTGGGCCTGCTCGGTGATTAATATTTAACAGCTGCGGTATTATAACTGTACTAAGGTAGCATAATA
ATTTGCTCTATAAATTGGGGCTAGAATGAATGGTTTGACGAAAATTTGACTGTCTCTATCTTATTTATTAGAAATTAATTTTTGTAGTGAGAAAGCTTAAATT
ATTTAAAGGGACGAAAAGACCCTATTGAGCTTGTATTATTTAACAAATTATATGATTGTTATTTGTTTGAATTAATTTTGATTGGGGTGATCAAGGAATAAAT
TTTATTTAGTTTAACTTCCTTAGTTAATTGTTTTATAGAAGAATAAACCAAAGTTTTTGCTTAGAAGATAAGTTACCATAGGGATAACAGCGTAATTTTTTTT
GAGAGTTCATATTGAAAAAAGAGATTGCGACCTCGATGTTGGATTAAAATTAACCTTA
>STR17716s
CCAAAAACATGTCTCTTTGAGATTTTTTAATAAAGAGTTGGGCCTGCTCGGTGATTAATATTTAACAGCTGCGGTATTATAACTGTACTAAGGTAGCATAATA
ATTTGCTCTATAAATTGGGGCTAGAATGAATGGTTTGACGAAAATTTGACTGTCTCTATCTTATTTATTAGAAATTAATTTTTGTAGTGAGAAAGCTTAAATT
ATTTAAAGGGACGAAAAGACCCTATTGAGCTTGTATTATTTAACAAATTATATGATTGTTATTTGTTTGAATTAATTTTGATTGGGGTGATCAAGGAATAAAT
TTTATTTAGTTTAACTTCCTTAATTAATTGTTTTATAGAAGAATAAACCAAAGTTTTTGCTTAGAAGATAAGTTACCATAGGGATAACAGCGTAATTTTTTTT
GAGAGTTCATATTGAAAAAAGAGATTGCGACCTCGATGTTGGATTAAAATTAACCTTA
>STR17816s
CCAAAAACATGTCTCTTTGAGATTTTTTAATAAAGAGTTGGGCCTGCTCGGTGATTAATATTTAACAGCTGCGGTATTATAACTGTACTAAGGTAGCATAATA
ATTTGCTCTATAAATTGGGGCTAGAATGAATGGTTTGACGAAAATTTGACTGTCTCTATCTTATTTATTAGAAATTAATTTTTGTAGTGAGAAAGCTTAAATT
ATTTAAAGGGACGAAAAGACCCTATTGAGCTTGTATTATTTAACAAATTATATGATTGTTATTTGTTTGAATTAATTTTGATTGGGGTGATCAAGGAATAAAT
TTTATTTAGTTTAACTTCCTTAGTTAATTGTTTTATAGAAGAATAAACCAAAGTTTTTGCTTAGAAGATAAGTTACCATAGGGATAACAGCGTAATTTTTTTT
GAGAGTTCATATTGAAAAAAGAGATTGCGACCTCGATGTTGGATTAAAATTAACCTTA
>STR17916s
CCAAAAACATGTCTCTTTGAGATTTTTTAATAAAGAGTTGGGCCTGCTCGGTGATTAATATTTAACAGCTGCGGTATTATAACTGTACTAAGGTAGCATAATA
ATTTGCTCTATAAATTGGGGCTAGAATGAATGGTTTGACGAAAATTTGACTGTCTCTATCTTATTTATTAGAAATTAATTTTTGTAGTGAGAAAGCTTAAATT
ATTTAAAGGGACGAAAAGACCCTATTGAGCTTGTATTATTTAACAAATTATATGATTGTTATTTGTTTGAATTAATTTTGATTGGGGTGATCAAGGAATAAAT
TTTATTTAGTTTAACTTCCTTAGTTAATTGTTTTATAGAAGAATAAACCAAAGTTTTTGCTTAGAAGATAAGTTACCATAGGGATAACAGCGTAATTTTTTTT
GAGAGTTCATATTGAAAAAAGAGATTGCGACCTCGATGTTGGATTAAAATTAACCTTA
>STR18016s
CCAAAAACATGTCTCTTTGAGATTTTTTAATAAAGAGTTGGGCCTGCTCGGTGATTAATATTTAACAGCTGCGGTATTATAACTGTACTAAGGTAGCATAATA
ATTTGCTCTATAAATTGGGGCTAGAATGAATGGTTTGACGAAAATTTGACTGTCTCTATCTTATTTATTAGAAATTAATTTTTGTAGTGAGAAAGCTTAAATT
ATTTAAAGGGACGAAAAGACCCTATTGAGCTTGTATTATTTAACAAATTATATGATTGTTATTTGTTTGAATTAATTTTGATTGGGGTGATCAAGGAATAAAT
TTTATTTAGTTTAACTTCCTTAGTTAATTGTTTTATAGAAGAATAAACCAAAGTTTTTGCTTAGAAGATAAGTTACCATAGGGATAACAGCGTAATTTTTTTT
GAGAGTTCATATTGAAAAAAGAGATTGCGACCTCGATGTTGGATTAAAATTAACCTTA
>STR18116s
CCAAAAACATGTCTCTTTGAGATTTTTTAATAAAGAGTTGGGCCTGCTCGGTGATTAATATTTAACAGCTGCGGTATTATAACTGTACTAAGGTAGCATAATA
ATTTGCTCTATAAATTGGGGCTAGAATGAATGGTTTGACGAAAATTTGACTGTCTCTATCTTATTTATTAGAAATTAATTTTTGTAGTGAGAAAGCTTAAATT
ATTTAAAGGGACGAAAAGACCCTATTGAGCTTGTATTATTTAACAAATTATATGATTGTTATTTGTTTGAATTAATTTTGATTGGGGTGATCAAGGAATAAAT
TTTATTTAGTTTAACTTCCTTAGTTAATTGTTTTATAGAAGAATAAACCAAAGTTTTTGCTTAGAAGATAAGTTACCATAGGGATAACAGCGTAATTTTTTTT
GAGAGTTCATATTGAAAAAAGAGATTGCGACCTCGATGTTGGATTAAAATTAACCTTA
>STR18216s
CCAAAAACATGTCTCTTTGAGATTTTTTAATAAAGAGTTGGGCCTGCTCGGTGATTAATATTTAACAGCTGCGGTATTATAACTGTACTAAGGTAGCATAATA
ATTTGCTCTATAAATTGGGGCTAGAATGAATGGTTTGACGAAAATTTGACTGTCTCTATCTTATTTATTAGAAATTAATTTTTGTAGTGAGAAAGCTTAAATT
ATTTAAAGGGACGAAAAGACCCTATTGAGCTTGTATTATTTAACAAATTATATGATTGTTATTTGTTTGAATTAATTTTGATTGGGGTGATCAAGGAATAAAT
TTTATTTAGTTTAACTTCCTTAGTTAATTGTTTTATAGAAGAATAAACCAAAGTTTTTGCTTAGAAGATAAGTTACCATAGGGATAACAGCGTAATTTTTTTT
GAGAGTTCATATTGAAAAAAGAGATTGCGACCTCGATGTTGGATTAAAATTAACCTTA

>STR18316s
CCAAAAACATGTCTCTTTGAGATTTTTTAATAAAGAGTTGGGCCTGCTCGGTGATTAATATTTAACAGCTGCGGTATTATAACTGTACTAAGGTAGCATAATA
ATTTGCTCTATAAATTGGGGCTAGAATGAATGGTTTGACGAAAATTTGACTGTCTCTATCTTATTTATTAGAAATTAATTTTTGTAGTGAGAAAGCTTAAATT
ATTTAAAGGGACGAAAAGACCCTATTGAGCTTGTATTATTTAACAAATTATATGATTGTTATTTGTTTGAATTAATTTTGATTGGGGTGATCAAGGAATAAAT
TTTATTTAGTTTAACTTCCTTAGTTAATTGTTTTATAGAAGAATAAACCAAAGTTTTTGCTTAGAAGATAAGTTACCATAGGGATAACAGCGTAATTTTTTTT
GAGAGTTCATATTGAAAAAAGAGATTGCGACCTCGATGTTGGATTAAAATTAACCTTA
>STR18416s
CCAAAAACATGTCTCTTTGAGATTTTTTAATAAAGAGTTGGGCCTGCTCGGTGATTAATATTTAACAGCTGCGGTATTATAACTGTACTAAGGTAGCATAATA
ATTTGCTCTATAAATTGGGGCTAGAATGAATGGTTTGACGAAAATTTGACTGTCTCTATCTTATTTATTAGAAATTAATTTTTGTAGTGAGAAAGCTTAAATT
ATTTAAAGGGACGAAAAGACCCTATTGAGCTTGTATTATTTAACAAATTATATGATTGTTATTTGTTTGAATTAATTTTGATTGGGGTGATCAAGGAATAAAT
TTTATTTAGTTTAACTTCCTTAATTAATTGTTTTATAGAAGAATAAACCAAAGTTTTTGCTTAGAAGATAAGTTACCATAGGGATAACAGCGTAATTTTTTTT
GAGAGTTCATATTGAAAAAAGAGATTGCGACCTCGATGTTGGATTAAAATTAACCTTA
>BKI39516s
CCAAAAACATGTCTCTTTGAGATTTTTTAATAAAGAGTTGGGCCTGCTCGGTGATTAATATTTAACAGCTGCGGTATTATAACTGTACTAAGGTAGCATAATA
ATTTGCTCTATAAATTGGGGCTAGAATGAATGGTTTGACGAAAATTTGACTGTCTCTATCTTATTTATTAGAAATTAATTTTTGTAGTGAGAAAGCTTAAATT
ATTTAAAGGGACGAAAAGACCCTATTGAGCTTGTATTATTTAACAAATTATATGATTGTTATTTGTTTGAATTAATTTTGATTGGGGTGATCAAGGAATAAAT
TTTATTTAGTTTAACTTCCTTAGTTAATTGTTTTATAGAAGAATAAACCAAAGTTTTTGCTTAGAAGATAAGTTACCATAGGGATAACAGCGTAATTTTTTTT
GAGAGTTCATATTGAAAAAAGAGATTGCGACCTCGATGTTGGATTAAAATTAACCTTA
>BKI39416s
CCAAAAACATGTCTCTTTGAGATTTTTTAATAAAGAGTTGGGCCTGCTCGGTGATTAATATTTAACAGCTGCGGTATTATAACTGTACTAAGGTAGCATAATA
ATTTGCTCTATAAATTGGGGCTAGAATGAATGGTTTGACGAAAATTTGACTGTCTCTATCTTATTTATTAGAAATTAATTTTTGTAGTGAGAAAGCTTAAATT
ATTTAAAGGGACGAAAAGACCCTATTGAGCTTGTATTATTTAACAAATTATATGATTGTTATTTGTTTGAATTAATTTTGATTGGGGTGATCAAGGAATAAAT
TTTATTTAGTTTAACTTCCTTAATTAATTGTTTTATAGAAGAATAAACCAAAGTTTTTGCTTAGAAGATAAGTTACCATAGGGATAACAGCGTAATTTTTTTT
GAGAGTTCATATTGAAAAAAGAGATTGCGACCTCGATGTTGGATTAAAATTAACCTTA
>BKI39316s
CCAAAAACATGTCTCTTTGAGATTTTTTAATAAAGAGTTGGGCCTGCTCGGTGATTAATATTTAACAGCTGCGGTATTATAACTGTACTAAGGTAGCATAATA
ATTTGCTCTATAAATTGGGGCTAGAATGAATGGTTTGACGAAAATTTGACTGTCTCTATCTTATTTATTAGAAATTAATTTTTGTAGTGAGAAAGCTTAAATT
ATTTAAAGGGACGAAAAGACCCTATTGAGCTTGTATTATTTAACAAATTATATGATTGTTATTTGTTTGAATTAATTTTGATTGGGGTGATCAAGGAATAAAT
TTTATTTAGTTTAACTTCCTTAATTAATTGTTTTATAGAAGAATAAACCAAAGTTTTTGCTTAGAAGATAAGTTACCATAGGGATAACAGCGTAATTTTTTTT
GAGAGTTCATATTGAAAAAAGAGATTGCGACCTCGATGTTGGATTAAAATTAACCTTA
>BKI39216s
CCAAAAACATGTCTCTTTGAGATTTTTTAATAAAGAGTTGGGCCTGCTCGGTGATTAATATTTAACAGCTGCGGTATTATAACTGTACTAAGGTAGCATAATA
ATTTGCTCTATAAATTGGGGCTAGAATGAATGGTTTGACGAAAATTTGACTGTCTCTATCTTATTTATTAGAAATTAATTTTTGTAGTGAGAAAGCTTAAATT
ATTTAAAGGGACGAAAAGACCCTATTGAGCTTGTATTATTTAACAAATTATATGATTGTTATTTGTTTGAATTAATTTTGATTGGGGTGATCAAGGAATAAAT
TTTATTTAGTTTAACTTCCTTAGTTAATTGTTTTATAGAAGAATAAACCAAAGTTTTTGCTTAGAAGATAAGTTACCATAGGGATAACAGCGTAATTTTTTTT
GAGAGTTCATATTGAAAAAAGAGATTGCGACCTCGATGTTGGATTAAAATTAACCTTA
>BKI39116s
CCAAAAACATGTCTCTTTGAGATTTTTTAATAAAGAGTTGGGCCTGCTCGGTGATTAATATTTAACAGCTGCGGTATTATAACTGTACTAAGGTAGCATAATA
ATTTGCTCTATAAATTGGGGCTAGAATGAATGGTTTGACGAAAATTTGACTGTCTCTATCTTATTTATTAGAAATTAATTTTTGTAGTGAGAAAGCTTAAATT
ATTTAAAGGGACGAAAAGACCCTATTGAGCTTGTATTATTTAACAAATTATATGATTGTTATTTGTTTGAATTAATTTTGATTGGGGTGATCAAGGAATAAAT
TTTATTTAGTTTAACTTCCTTAGTTAATTGTTTTATAGAAGAATAAACCAAAGTTTTTGCTTAGAAGATAAGTTACCATAGGGATAACAGCGTAATTTTTTTT
GAGAGTTCATATTGAAAAAAGAGATTGCGACCTCGATGTTGGATTAAAATTAACCTTA
>BKI39016s
CCAAAAACATGTCTCTTTGAGATTTTTTAATAAAGAGTTGGGCCTGCTCGGTGATTAATATTTAACAGCTGCGGTATTATAACTGTACTAAGGTAGCATAATA
ATTTGCTCTATAAATTGGGGCTAGAATGAATGGTTTGACGAAAATTTGACTGTCTCTATCTTATTTATTAGAAATTAATTTTTGTAGTGAGAAAGCTTAAATT
ATTTAAAGGGACGAAAAGACCCTATTGAGCTTGTATTATTTAACAAATTATATGATTGTTATTTGTTTGAATTAATTTTGATTGGGGTGATCAAGGAATAAAT
TTTATTTAGTTTAACTTCCTTAATTAATTGTTTTATAGAAGAATAAACCAAAGTTTTTGCTTAGAAGATAAGTTACCATAGGGATAACAGCGTAATTTTTTTT
GAGAGTTCATATTGAAAAAAGAGATTGCGACCTCGATGTTGGATTAAAATTAACCTTA
>BKI38916s
CCAAAAACATGTCTCTTTGAGATTTTTTAATAAAGAGTTGGGCCTGCTCGGTGATTAATATTTAACAGCTGCGGTATTATAACTGTACTAAGGTAGCATAATA
ATTTGCTCTATAAATTGGGGCTAGAATGAATGGTTTGACGAAAATTTGACTGTCTCTATCTTATTTATTAGAAATTAATTTTTGTAGTGAGAAAGCTTAAATT
ATTTAAAGGGACGAAAAGACCCTATTGAGCTTGTATTATTTAACAAATTATATGATTGTTATTTGTTTGAATTAATTTTGATTGGGGTGATCAAGGAATAAAT
TTTATTTAGTTTAACTTCCTTAATTAATTGTTTTATAGAAGAATAAACCAAAGTTTTTGCTTAGAAGATAAGTTACCATAGGGATAACAGCGTAATTTTTTTT
GAGAGTTCATATTGAAAAAAGAGATTGCGACCTCGATGTTGGATTAAAATTAACCTTA
>BKI38816s
CCAAAAACATGTCTCTTTGAGATTTTTTAATAAAGAGTTGGGCCTGCTCGGTGATTAATATTTAACAGCTGCGGTATTATAACTGTACTAAGGTAGCATAATA
ATTTGCTCTATAAATTGGGGCTAGAATGAATGGTTTGACGAAAATTTGACTGTCTCTATCTTATTTATTAGAAATTAATTTTTGTAGTGAGAAAGCTTAAATT
ATTTAAAGGGACGAAAAGACCCTATTGAGCTTGTATTATTTAATAAATTATATGATTGTTATTTGTTTGAATTAATTTTGATTGGGGTGATCAAGGAATAAAT
TTTATTTAGTTTAACTTCCTTAGTTAATTGTTTTATAGAAGAATAAACCAAAGTTTTTGCTTAGAAGATAAGTTACCATAGGGATAACAGCGTAATTTTTTTT
GAGAGTTCATATTGAAAAAAGAGATTGCGACCTCGATGTTGGATTAAAATTAACCTTA
>ISL42016s
CCAAAAACATGTCTCTTTGAGATTTTTTAATAAAGAGTTGGGCCTGCTCGGTGATTAATATTTAACAGCTGCGGTATTATAACTGTACTAAGGTAGCATAATA
ATTTGCTCTATAAATTGGGGCTAGAATGAATGGTTTGACGAAAATTTGACTGTCTCTATCTTATTTATTAGAAATTAATTTTTGTAGTGAGAAAGCTTAAATT
ATTTAAAGGGACGAAAAGACCCTATTGAGCTTGTATTATTTAACAAATTATATGATTGTTATTTGTTTGAATTAATTTTGATTGGGGTGATCAAGGAATAAAT
TTTATTTAGTTTAACTTCCTTAATTAATTGTTTTATAGAAGAATAAACCAAAGTTTTTGCTTAGAAGATAAGTTACCATAGGGATAACAGCGTAATTTTTTTT
GAGAGTTCATATTGAAAAAAGAGATTGCGACCTCGATGTTGGATTAAAATTAACCTTA
>ISL42116s
CCAAAAACATGTCTCTTTGAGATTTTTTAATAAAGAGTTGGGCCTGCTCGGTGATTAATATTTAACAGCTGCGGTATTATAACTGTACTAAGGTAGCATAATA
ATTTGCTCTATAAATTGGGGCTAGAATGAATGGTTTGACGAAAATTTGACTGTCTCTATCTTATTTATTAGAAATTAATTTTTGTAGTGAGAAAGCTTAAATT
ATTTAAAGGGACGAAAAGACCCTATTGAGCTTGTATTATTTAACAAATTATATGATTGTTATTTGTTTGAATTAATTTTGATTGGGGTGATCAAGGAATAAAT
TTTATTTAGTTTAACTTCCTTAGTTAATTGTTTTATAGAAGAATAAACCAAAGTTTTTGCTTAGAAGATAAGTTACCATAGGGATAACAGCGTAATTTTTTTT
GAGAGTTCATATTGAAAAAAGAGATTGCGACCTCGATGTTGGATTAAAATTAACCTTA
>ISL42216s
CCAAAAACATGTCTCTTTGAGATTTTTTAATAAAGAGTTGGGCCTGCTCGGTGATTAATATTTAACAGCTGCGGTATTATAACTGTACTAAGGTAGCATAATA
ATTTGCTCTATAAATTGGGGCTAGAATGAATGGTTTGACGAAAATTTGACTGTCTCTATCTTATTTATTAGAAATTAATTTTTGTAGTGAGAAAGCTTAAATT
ATTTAAAGGGACGAAAAGACCCTATTGAGCTTGTATTATTTAACAAATTATATGATTGTTATTTGTTTGAATTAATTTTGATTGGGGTGATCAAGGAATAAAT
TTTATTTAGTTTAACTTCCTTAGTTAATTGTTTTATAGAAGAATAAACCAAAGTTTTTGCTTAGAAGATAAGTTACCATAGGGATAACAGCGTAATTTTTTTT
GAGAGTTCATATTGAAAAAAGAGATTGCGACCTCGATGTTGGATTAAAATTAACCTTA
>ISL42316s

CCAAAAACATGTCTCTTTGAGATTTTTTAATAAAGAGTTGGGCCTGCTCGGTGATTAATATTTAACAGCTGCGGTATTATAACTGTACTAAGGTAGCATAATA
ATTTGCTCTATAAATTGGGGCTAGAATGAATGGTTTGACGAAAATTTGACTGTCTCTATCTTATTTATTAGAAATTAATTTTTGTAGTGAGAAAGCTTAAATT
ATTTAAAGGGACGAAAAGACCCTATTGAGCTTGTATTATTTAACAAATTATATGATTGTTATTTGTTTGAATTAATTTTGATTGGGGTGATCAAGGAATAAAT
TTTATTTAGTTTAACTTCCTTAGTTAATTGTTTTATAGAAGAATAAACCAAAGTTTTTGCTTAGAAGATAAGTTACCATAGGGATAACAGCGTAATTTTTTTT
GAGAGTTCATATTGAAAAAAGAGATTGCGACCTCGATGTTGGATTAAAATTAACCTTA
>ISL42416s
CCAAAAACATGTCTCTTTGAGATTTTTTAATAAAGAGTTGGGCCTGCTCGGTGATTAATATTTAACAGCTGCGGTATTATAACTGTACTAAGGTAGCATAATA
ATTTGCTCTATAAATTGGGGCTAGAATGAATGGTTTGACGAAAATTTGACTGTCTCTATCTTATTTATTAGAAATTAATTTTTGTAGTGAGAAAGCTTAAATT
ATTTAAAGGGACGAAAAGACCCTATTGAGCTTGTATTATTTAACAAATTATATGATTGTTATTTGTTTGAATTAATTTTGATTGGGGTGATCAAGGAATAAAT
TTTATTTAGTTTAACTTCCTTAATTAATTGTTTTATAGAAGAATAAACCAAAGTTTTTGCTTAGAAGATAAGTTACCATAGGGATAACAGCGTAATTTTTTTT
GAGAGTTCATATTGAAAAAAGAGATTGCGACCTCGATGTTGGATTAAAATTAACCTTA
>ISL42516s
CCAAAAACATGTCTCTTTGAGATTTTTTAATAAAGAGTTGGGCCTGCTCGGTGATTAATATTTAACAGCTGCGGTATTATAACTGTACTAAGGTAGCATAATA
ATTTGCTCTATAAATTGGGGCTAGAATGAATGGTTTGACGAAAATTTGACTGTCTCTATCTTATTTATTAGAAATTAATTTTTGTAGTGAGAAAGCTTAAATT
ATTTAAAGGGACGAAAAGACCCTATTGAGCTTGTATTATTTAACAAATTATATGATTGTTATTTGTTTGAATTAATTTTGATTGGGGTGATCAAGGAATAAAT
TTTATTTAGTTTAACTTCCTTAGTTAATTGTTTTATAGAAGAATAAACCAAAGTTTTTGCTTAGAAGATAAGTTACCATAGGGATAACAGCGTAATTTTTTTT
GAGAGTTCATATTGAAAAAAGAGATTGCGACCTCGATGTTGGATTAAAATTAACCTTA
>ISL42616s
CCAAAAACATGTCTCTTTGAGATTTTTTAATAAAGAGTTGGGCCTGCTCGGTGATTAATATTTAACAGCTGCGGTATTATAACTGTACTAAGGTAGCATAATA
ATTTGCTCTATAAATTGGGGCTAGAATGAATGGTTTGACGAAAATTTGACTGTCTCTATCTTATTTATTAGAAATTAATTTTTGTAGTGAGAAAGCTTAAATT
ATTTAAAGGGACGAAAAGACCCTATTGAGCTTGTATTATTTAACAAATTATATGATTGTTATTTGTTTGAATTAATTTTGATTGGGGTGATCAAGGAATAAAT
TTTATTTAGTTTAACTTCCTTAGTTAATTGTTTTATAGAAGAATAAACCAAAGTTTTTGCTTAGAAGATAAGTTACCATAGGGATAACAGCGTAATTTTTTTT
GAGAGTTCATATTGAAAAAAGAGATTGCGACCTCGATGTTGGATTAAAATTAACCTTA
>ISL42716s
CCAAAAACATGTCTCTTTGAGATTTTTTAATAAAGAGTTGGGCCTGCTCGGTGATTAATATTTAACAGCTGCGGTATTATAACTGTACTAAGGTAGCATAATA
ATTTGCTCTATAAATTGGGGCTAGAATGAATGGTTTGACGAAAATTTGACTGTCTCTATCTTATTTATTAGAAATTAATTTTTGTAGTGAGAAAGCTTAAATT
ATTTAAAGGGACGAAAAGACCCTATTGAGCTTGTATTATTTAATAAATTATATAATTGTTATTTGTTTGAATTAATTTTGATTGGGGTGATCAAGGAATAAAT
TTTATTTAGTTTAACTTCCTTAGTTAGTTGTTTTATAGAAGAATAAACCAAAGTTTTTGCTTAGAAGATAAGTTACCATAGGGATAACAGCGTAATTTTTTTT
GAGAGTTCATATTGAAAAAAGAGATTGCGACCTCGATGTTGGATTAAAATTAACCTTA
