# Supplementary material for: Life histories predict genetic diversity and population structure within three species of octopus targeted by small-scale fisheries in Northwest Mexico
Source: PeerJ. 2018 Feb 15;6:e4295. doi: 10.7717/peerj.4295 (PMC5816968; doi:10.7717/peerj.4295)
Supplement: Supplemental Information 4 [file peerj-06-4295-s011.pdf]

COI_Octopuses.sqn	STR184	KY985005
COI_Octopuses.sqn	STR183	KY985006
COI_Octopuses.sqn	STR182	KY985007
COI_Octopuses.sqn	STR181	KY985008
COI_Octopuses.sqn	STR180	KY985009
COI_Octopuses.sqn	STR179	KY985010
COI_Octopuses.sqn	STR178	KY985011
COI_Octopuses.sqn	STR177	KY985012
COI_Octopuses.sqn	SQ96	KY985013
COI_Octopuses.sqn	SQ95	KY985014
COI_Octopuses.sqn	SQ94	KY985015
COI_Octopuses.sqn	SQ93	KY985016
COI_Octopuses.sqn	SQ92	KY985017
COI_Octopuses.sqn	SQ91	KY985018
COI_Octopuses.sqn	SQ90	KY985019
COI_Octopuses.sqn	SQ89	KY985020
COI_Octopuses.sqn	PRE268	KY985021
COI_Octopuses.sqn	PPE304	KY985022
COI_Octopuses.sqn	PPE303	KY985023
COI_Octopuses.sqn	PPE302	KY985024
COI_Octopuses.sqn	PPE301	KY985025
COI_Octopuses.sqn	PPE300	KY985026
COI_Octopuses.sqn	PPE299	KY985027
COI_Octopuses.sqn	PPE298	KY985028
COI_Octopuses.sqn	PPE297	KY985029
COI_Octopuses.sqn	PLO363	KY985030
COI_Octopuses.sqn	PLO362	KY985031
COI_Octopuses.sqn	PLO361	KY985032
COI_Octopuses.sqn	PLO360	KY985033
COI_Octopuses.sqn	PLO359	KY985034
COI_Octopuses.sqn	PLO358	KY985035
COI_Octopuses.sqn	PLO357	KY985036
COI_Octopuses.sqn	PLO356	KY985037
COI_Octopuses.sqn	PLI450	KY985038
COI_Octopuses.sqn	PLI449	KY985039
COI_Octopuses.sqn	PLI448	KY985040
COI_Octopuses.sqn	PLI447	KY985041
COI_Octopuses.sqn	PLI446	KY985042
COI_Octopuses.sqn	PLI444	KY985043
COI_Octopuses.sqn	PLI443	KY985044
COI_Octopuses.sqn	MAL126	KY985045
COI_Octopuses.sqn	MAL125	KY985046
COI_Octopuses.sqn	MAL124	KY985047
COI_Octopuses.sqn	MAL123	KY985048
COI_Octopuses.sqn	MAL121	KY985049
COI_Octopuses.sqn	MAL120	KY985050
COI_Octopuses.sqn	MAL119	KY985051
COI_Octopuses.sqn	ISLG490	KY985052
COI_Octopuses.sqn	ISLG489	KY985053
COI_Octopuses.sqn	ISLG487	KY985054
COI_Octopuses.sqn	ISLG486	KY985055
COI_Octopuses.sqn	ISLG485	KY985056
COI_Octopuses.sqn	ISLG484	KY985057
COI_Octopuses.sqn	ISLG483	KY985058
COI_Octopuses.sqn	ISL427	KY985059
COI_Octopuses.sqn	ISL426	KY985060
COI_Octopuses.sqn	ISL425	KY985061
COI_Octopuses.sqn	ISL424	KY985062
COI_Octopuses.sqn	ISL423	KY985063
COI_Octopuses.sqn	ISL422	KY985064
COI_Octopuses.sqn	ISL421	KY985065
COI_Octopuses.sqn	ISL420	KY985066
COI_Octopuses.sqn	EE82	KY985067
COI_Octopuses.sqn	EE81	KY985068
COI_Octopuses.sqn	EE80	KY985069
COI_Octopuses.sqn	EE79	KY985070
COI_Octopuses.sqn	EE77	KY985071
COI_Octopuses.sqn	EE76	KY985072
COI_Octopuses.sqn	EE75	KY985073
COI_Octopuses.sqn	BM23	KY985074
COI_Octopuses.sqn	BM22	KY985075
COI_Octopuses.sqn	BM21	KY985076
COI_Octopuses.sqn	BM20	KY985077
COI_Octopuses.sqn	BM19	KY985078
COI_Octopuses.sqn	BM18	KY985079
COI_Octopuses.sqn	BM17	KY985080
COI_Octopuses.sqn	BM16	KY985081
COI_Octopuses.sqn	BLA290	KY985082
COI_Octopuses.sqn	BLA289	KY985083

COI_Octopuses.sqn	BLA288	KY985084
COI_Octopuses.sqn	BLA287	KY985085
COI_Octopuses.sqn	BLA286	KY985086
COI_Octopuses.sqn	BLA285	KY985087
COI_Octopuses.sqn	BLA284	KY985088
COI_Octopuses.sqn	BLA283	KY985089
COI_Octopuses.sqn	BKI395	KY985090
COI_Octopuses.sqn	BKI394	KY985091
COI_Octopuses.sqn	BKI393	KY985092
COI_Octopuses.sqn	BKI392	KY985093
COI_Octopuses.sqn	BKI391	KY985094
COI_Octopuses.sqn	BKI390	KY985095
COI_Octopuses.sqn	BKI389	KY985096
COI_Octopuses.sqn	BKI388	KY985097
Octopus_16s.sqn	SQ9616s	KY985098
Octopus_16s.sqn	SQ9516s	KY985099
Octopus_16s.sqn	SQ9416s	KY985100
Octopus_16s.sqn	SQ9316s	KY985101
Octopus_16s.sqn	SQ9216s	KY985102
Octopus_16s.sqn	SQ9116s	KY985103
Octopus_16s.sqn	SQ9016s	KY985104
Octopus_16s.sqn	SQ8916s	KY985105
Octopus_16s.sqn	BM1616s	KY985106
Octopus_16s.sqn	BM1716s	KY985107
Octopus_16s.sqn	BM1816s	KY985108
Octopus_16s.sqn	BM1916s	KY985109
Octopus_16s.sqn	BM2016s	KY985110
Octopus_16s.sqn	BM2116s	KY985111
Octopus_16s.sqn	BM2216s	KY985112
Octopus_16s.sqn	BM2316s	KY985113
Octopus_16s.sqn	EE7516s	KY985114
Octopus_16s.sqn	EE7616s	KY985115
Octopus_16s.sqn	EE7716s	KY985116
Octopus_16s.sqn	EE7816s	KY985117
Octopus_16s.sqn	EE7916s	KY985118
Octopus_16s.sqn	EE8016s	KY985119
Octopus_16s.sqn	EE8116s	KY985120
Octopus_16s.sqn	EE8216s	KY985121
Octopus_16s.sqn	BLA28316s	KY985122
Octopus_16s.sqn	BLA28516s	KY985123
Octopus_16s.sqn	BLA28616s	KY985124
Octopus_16s.sqn	BLA28716s	KY985125
Octopus_16s.sqn	BLA28816s	KY985126
Octopus_16s.sqn	BLA28916s	KY985127
Octopus_16s.sqn	BLA28416s	KY985128
Octopus_16s.sqn	BLA29016s	KY985129
Octopus_16s.sqn	ISLG48316s	KY985130
Octopus_16s.sqn	ISLG48416s	KY985131
Octopus_16s.sqn	ISLG48516s	KY985132
Octopus_16s.sqn	ISLG48616s	KY985133
Octopus_16s.sqn	ISLG48716s	KY985134
Octopus_16s.sqn	ISLG48816s	KY985135
Octopus_16s.sqn	ISLG48916s	KY985136
Octopus_16s.sqn	ISLG49016s	KY985137
Octopus_16s.sqn	MAL11916s	KY985138
Octopus_16s.sqn	MAL12016s	KY985139
Octopus_16s.sqn	MAL12116s	KY985140
Octopus_16s.sqn	MAL12216s	KY985141
Octopus_16s.sqn	MAL12316s	KY985142
Octopus_16s.sqn	MAL12416s	KY985143
Octopus_16s.sqn	MAL12516s	KY985144
Octopus_16s.sqn	MAL12616s	KY985145
Octopus_16s.sqn	PLO35616s	KY985146
Octopus_16s.sqn	PLO35716s	KY985147
Octopus_16s.sqn	PLO35816s	KY985148
Octopus_16s.sqn	PLO35916s	KY985149
Octopus_16s.sqn	PLO36016s	KY985150
Octopus_16s.sqn	PLO36116s	KY985151
Octopus_16s.sqn	PLO36216s	KY985152
Octopus_16s.sqn	PLO36316s	KY985153
Octopus_16s.sqn	PPE29716s	KY985154
Octopus_16s.sqn	PPE29816s	KY985155
Octopus_16s.sqn	PPE29916s	KY985156
Octopus_16s.sqn	PPE30016s	KY985157
Octopus_16s.sqn	PPE30116s	KY985158
Octopus_16s.sqn	PPE30216s	KY985159
Octopus_16s.sqn	PPE30316s	KY985160
Octopus_16s.sqn	PPE30416s	KY985161
Octopus_16s.sqn	PRE26816s	KY985162

Octopus_16s.sqn	PLI44116s	KY985163
Octopus_16s.sqn	PLI44316s	KY985164
Octopus_16s.sqn	PLI44416s	KY985165
Octopus_16s.sqn	PLI44616s	KY985166
Octopus_16s.sqn	PLI44716s	KY985167
Octopus_16s.sqn	PLI44816s	KY985168
Octopus_16s.sqn	PLI44916s	KY985169
Octopus_16s.sqn	PLI45016s	KY985170
Octopus_16s.sqn	STR17716s	KY985171
Octopus_16s.sqn	STR17816s	KY985172
Octopus_16s.sqn	STR17916s	KY985173
Octopus_16s.sqn	STR18016s	KY985174
Octopus_16s.sqn	STR18116s	KY985175
Octopus_16s.sqn	STR18216s	KY985176
Octopus_16s.sqn	STR18316s	KY985177
Octopus_16s.sqn	STR18416s	KY985178
Octopus_16s.sqn	BKI39516s	KY985179
Octopus_16s.sqn	BKI39416s	KY985180
Octopus_16s.sqn	BKI39316s	KY985181
Octopus_16s.sqn	BKI39216s	KY985182
Octopus_16s.sqn	BKI39116s	KY985183
Octopus_16s.sqn	BKI39016s	KY985184
Octopus_16s.sqn	BKI38916s	KY985185
Octopus_16s.sqn	BKI38816s	KY985186
Octopus_16s.sqn	ISL42016s	KY985187
Octopus_16s.sqn	ISL42116s	KY985188
Octopus_16s.sqn	ISL42216s	KY985189
Octopus_16s.sqn	ISL42316s	KY985190
Octopus_16s.sqn	ISL42416s	KY985191
Octopus_16s.sqn	ISL42516s	KY985192
Octopus_16s.sqn	ISL42616s	KY985193
Octopus_16s.sqn	ISL42716s	KY985194
